# The influence of the cationic carbenes on the initiation kinetics of ruthenium-based metathesis catalysts; a DFT study

**DOI:** 10.3762/bjoc.14.266

**Published:** 2018-11-20

**Authors:** Magdalena Jawiczuk, Angelika Janaszkiewicz, Bartosz Trzaskowski

**Affiliations:** 1 Centre of New Technologies, University of Warsaw, Banacha 2c, 02-097 Warszawa, Poland

**Keywords:** catalysts, cationic carbenes, DFT, initiation, metathesis

## Abstract

Cationic carbenes are a relatively new and rare group of ancillary ligands, which have shown their superior activity in a number of challenging catalytic reactions. In ruthenium-based metathesis catalysis they are often used as ammonium tags, to provide water-soluble, environment-friendly catalysts. In this work we performed computational studies on three cationic carbenes with the formal positive charge located at different distances from the carbene carbon. We show that the predicted initiation rates of Grubbs, indenylidene, and Hoveyda–Grubbs-like complexes incorporating these carbenes show little variance and are similar to initiation rates of standard Grubbs, indenylidene, and Hoveyda–Grubbs catalysts. In all investigated cases the partial charge of the carbene carbon atom is similar, resulting in comparable C_carbene_–Ru bond strengths and Ru–P/O dissociation Gibbs free energies.

## Introduction

The isolation of the first stable N-heterocyclic carbene (NHC) by Arduengo [1] was a milestone in organic chemistry which allowed for thorough and systematic studies on all aspects of NHC chemistry in the past 25 years [2–7]. It was soon realized that NHCs are a very useful class of ligands for transition metal catalysis as both their steric and electronic properties can be easily controlled and tuned to obtain very efficient and specific catalysts. One of the most successful uses of NHCs in catalysis is the olefin metathesis, which nowadays became one of the most commonly used tool in modern synthesis [8–10]. The vast popularity of metathesis results from the high stabilities and efficiencies of Ruthenium catalysts stabilised by NHC moieties. In this class of compounds NHC ligands, with the poor π-acceptor and strong σ-donor properties, stabilize the 14-electron ruthenium active species during the catalytic cycle [11–12]. Today there are hundreds of examples of second generation Grubbs and Hoveyda–Grubbs catalyst derivatives bearing different NHCs to form specialized catalysts for metathesis [13–14].

An interesting attempt to further modify the electronic properties of NHCs is to introduce a charged moiety to form either anionic or cationic carbenes [15–18]. Cationic ligands with a positive charge close to the coordinating atom are relatively rare, as their coordination ability of transition metals, bearing also a formal positive charge, is weakened. Nevertheless, stable metal complexes with cationic ligands have been synthesized and used in catalysis [19–21]. With respect to olefin metathesis cationic carbenes have been introduced as early as in 2007, where Grubbs described the first ammonium-tagged Hoveyda-type catalyst [22]. The goal of that study was to develop systems that are active and stable in water and, therefore, environmentally-friendly. The idea of incorporating a quaternary ammonium moiety into the imidazole part of the carbene was later expanded by several other groups, including a number of new water-soluble catalysts synthesized by Skowerski et al. [23–24]. In the meantime Schanz and co-workers synthesized also Hoveyda-like complexes with ammonium groups introduced into the aryl rings of the NHC ligands [25]. Most of these complexes showed good efficiency in selected metathesis reactions.

Interestingly in all reported cases of ammonium tagged Ru–alkylidene metathesis catalysts the ammonium tag is relatively far from the carbene carbon atom chelating the ruthenium core. The reason behind such design was likely the low probability of the ammonium tag influencing the ruthenium core and therefore, having a potential negative effect on the efficiency and reaction rate of the catalyst as well as the ease of synthesis. In 2013 Kośnik and Grela performed a study to check the influence of the length of the spacer between the NHC ligand and the onium tag, by synthesizing the tag with an eight –(CH_2_)– linker [26]. The authors concluded that the extension of the linker does not affect the efficiency of the catalyst in model metathesis reactions in comparison to Skowerski’s complexes with only one –CH_2_– unit. Curiously, carbenes with the cationic group even closer to the imidazole moiety (with no spacer) or incorporated into the imidazole core have been synthesized only very recently and examples of their transition metal complexes are scarce. In 2013 Ganter described a cationic NHC with a fused pyridinium moiety and the formal +1 charge just one bond away from the imidazole core [27]. In 2017 César synthesized a cationic imidazolylidene NHC with an ammonium tag attached directly to the imidazole core [28]. Finally, in the same year Ganter described a triazoliumylidene with the formal +1 charge incorporated into the five-membered ring [29]. Several complexes formed by these carbenes have been also described, however, no ruthenium complexes with such carbenes have been synthesized.

**Scheme 1:**
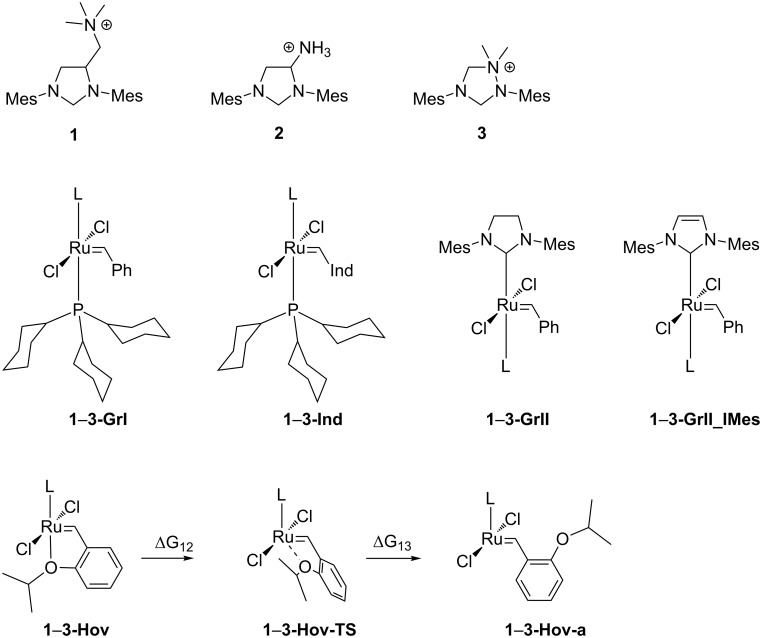
NHC’s and their ruthenium complexes studied in this work; L = carbene **1**, **2** or **3**.

In this work we have performed a systematic study of three cationic carbenes with the formal +1 charge located at different distances from the carbene carbon atom using a computational approach (Scheme 1). We considered the impact of the positive charge on the electronic properties of carbenes, but also on the properties and initiation rates of the most important ruthenium-based metathesis catalysts, including Grubbs, indenylidene, and Hoveyda–Grubbs complexes, as well as carbene dimerization. We also considered two different solvents: dichloromethane, which is a standard solvent for performing metathesis reactions and water, which is commonly used in the case of ammonium-tagged metathesis catalysts.

## Results and Discussion

### Computational benchmarks

The M06 method has become the method of choice for obtaining accurate energies for ruthenium metathesis for a number of groups investigating this class of catalysts [30–44]. Since the M06 functional already includes some medium-range dispersion it is usually used without additional corrections to better describe dispersion interactions. The commonly used D3 semiempirical correction for density functionals has been, however, derived also for the M06 functional and shown to improve results for many organic reactions when calculating the differences in relative energies [45–46]. Others have, however, pointed out that M06-D3 may overestimate the effect of dispersion due to double-counting of these effects [47]. To resolve this issue we performed benchmark calculations for standard metathesis catalyst **GrI**, as well as newly developed catalyst featuring a labile carbodicarbene ligand (as a model of **1–3-GrII**) [48]. In the case of **GrI** we found the Gibbs free energy of initiation in the M06 method equal to 20.4 kcal/mol, in perfect agreement with the experimental value of 19.88 kcal/mol [21]. The addition of the D3 dispersion correction increases this value to 29.2 kcal/mol. For the carbodicarbene catalyst the experimental value is 23.5 kcal/mol [48] and we found the value of 23.9 kcal/mol, using M06-D3 approach. Previously we have shown that the addition of D3 correction gives very good agreement with the experimental data for **Hov** and Hoveyda-like systems as well as for investigations of carbenes dimerization [38,49]. As a result we decided to use the M06-D3 functional in calculations of Gibbs free energies all system apart from 1st generation Grubbs and indenylidene-like complexes, for which we used pure M06. Results for all systems and both M06 and M06-D3 methods are listed in Supporting Information File 1.

### Dimerization

**Scheme 2:**
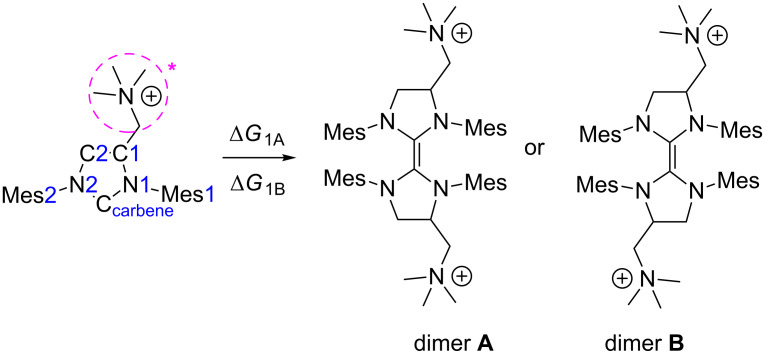
Schematic representation of carbene dimerization and atom numbering scheme used throughout this work.

The tendency of selected NHC to dimerize is a well-known and interesting phenomenon, despite its very limited impact on their propensity to form transition metal complexes (Scheme 2) [50]. Many works have been devoted to the study of carbene dimerization and present evidence that mechanism of monomer–dimer equilibrium depends on the balance between the electronic and steric properties of NHCs [49,51–54]. In general, all unsaturated carbenes have strong preference for the monomeric form due to the electronic effect. On the other hand saturated carbenes prefer the dimeric form if either their side-groups are relatively small (e.g., methyl or ethyl) or if the carbenes are asymmetric [55–56]. Unfortunately the subtle Wanzlick equilibrium between many saturated carbenes may easily shift to either the dimeric or monomeric form with a small structural change and it is not a trivial task to predict the more stable form of the carbene based solely on its structural features.

Since all investigated carbenes are asymmetric we considered the possibility of formation of two different dimers, marked **A** (symmetric) and **B** (asymmetric), respectively (Table 1). Results obtained for carbenes **1** and **2** suggest a strong preference for both systems to dimerize, with a slightly lower Gibbs free energy difference for the symmetric dimer **A**. In the case of carbene **3**, the Gibbs free energy of dimerization could not be estimated due to instability of the monomer during geometry optimization. Thus, the results indicate higher stability of dimers for all examined NHC, which are in agreement with previous literature reports for asymmetrical N-heterocyclic carbenes, as well as accurate DLPNO-CCSD(T) calculations (see Supporting Information File 1) [49,55]. The change of the solvent from CH_2_Cl_2_ to water only slightly altered the calculated dimerization energies and also indicated higher stabilities of dimers on solution (see Supporting Information File 1).

**Table 1:** Calculated dimerization energies (∆*G*_1_) in CH_2_Cl_2_ for carbenes **1** and **2** and the C_carbene_–C’_carbene_ bond lengths of all corresponding dimers.

structure	∆*G*_1_ [kcal/mol]	C_carbene_–C’_carbene_ [Å]

**1A**	−9.4	1.362
**1B**	−9.0	1.361
**2A**	−10.3	1.362
**2B**	−10.0	1.359
**3A**	–	1.365
**3B**	–	1.365

### First generation Grubbs and M1 indenylidene catalyst

**Scheme 3:**
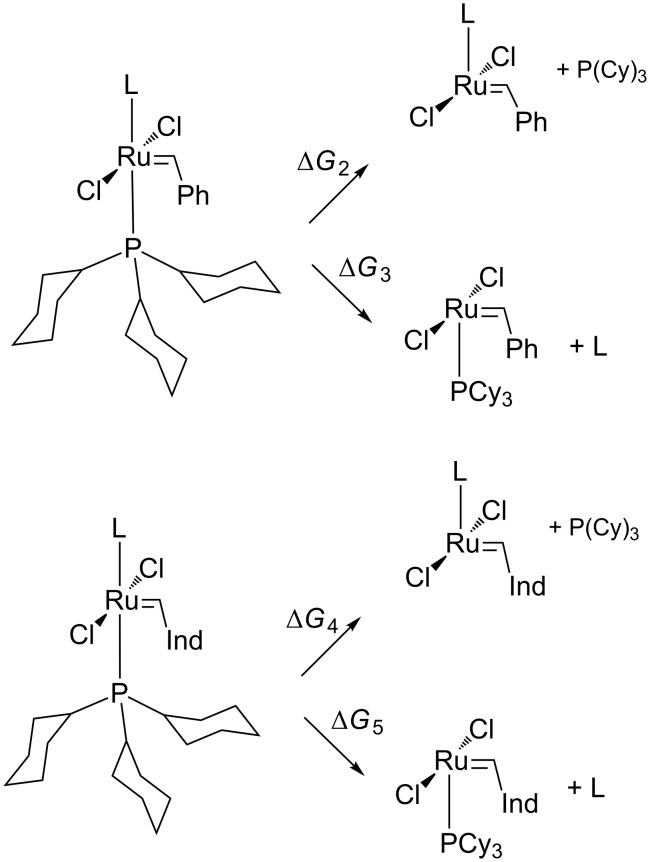
Dissociative mechanism of initiation for Grubbs-like **1**–**3-GrI** and **M1** indenylidene type complexes **1**–**3-Ind**; L = carbene **1**, **2** or **3**.

In the next step of the study we performed a computational investigation of possible pathways of the initiation of cationic ruthenium catalyst based on the commonly used 1st generation Grubbs catalyst (**GrI**) and **M1** indenylidene catalyst (**Ind**). New complexes were formed by replacing one PCy_3_ phosphine ligand with the cationic NHC **1**–**3** (Scheme 1). We considered only the dissociative mechanism of initiation, in agreement with the numerous reports on the initiation of Grubbs catalyst [57], but we also considered the possibility of cationic carbene dissociation as the first step of the metathesis catalytic cycle (Scheme 3).

The results of the computational study are presented in Table 2 and show that in all cases the energy barriers for the dissociation of phosphine ligand (∆*G*_2_) are 0.4–3.1 kcal/mol lower compared to carbene dissociation (∆*G*_3_). We can speculate that the positive charge of carbenes **1**–**3** lowers the Ru–C bond strength, making it easier to dissociate than for neutral carbenes. Interestingly, the estimate of the Gibbs free energy of initiation for complex **3-GrI** suggest faster activation than first generation Grubbs catalysts, for which the experimental value of ∆*G*_2_ was found at 19.88 kcal/mol [58].

**Table 2:** The comparison of dissociation energies ∆*G*_2_–∆*G*_5_ and structural parameters of investigated compounds.

complex	∆*G*_2_ [kcal/mol]	∆*G*_3_ [kcal/mol]	Ru–P [Å]	Ru–C_carbene_ [Å]

**1-GrI**	20.9	24.0	2.464	2.056
**2-GrI**	23.3	23.7	2.478	2.036
**3-GrI**	18.7	–	2.466	2.056
**GrI**^a^ exp.	19.88	–	2.435	2.397
**GrI calculated**	18.9	18.9	2.440	2.434

complex	∆*G*_4_ [kcal/mol]	∆*G*_5_ [kcal/mol]	Ru–P [Å]	Ru–C_carbene_ [Å]

**1-Ind**	18.7	25.6	2.470	2.073
**2-Ind**	16.7	22.5	2.487	2.056
**3-Ind**	21.9	–	2.478	2.084
**Ind**^b^ exp.	21	–	2.410	2.415

^a^See ref. [58]; ^b^See refs. [59–60].

Similarly, in the case of indenylidene complexes (**1**–**3-Ind**) the dissociation of phosphine is also preferred over the loss of the cationic carbene. For this series of complexes **3-Ind** displays the activation Gibbs free energy ∆*G*_4_ (21.9 kcal/mol) very similar to **Ind**, for which it was experimentally determined at 21 kcal/mol [60]. Both **2-Ind** and **1-Ind** show, however, longer Ru–P bonds and lower estimates of activation Gibbs free energies, suggesting their relatively fast activation during the catalytic cycle. The estimates of free energies in water follow exactly the same trends, although are always a few kcal/mol lower, indicating that in this solvent Grubbs-like complexes may initiate faster (see Supporting Information File 1).

It is worth mentioning that for the 1st generation Grubbs complexes the DLPNO-CCSD(T) results give consistently Gibbs free energy value which are 8–12 kcal/mol higher than those obtained using DFT approach. This is also true for **GrI** for which the computational DLPNO-CCSD(T) method gives the 28.1 kcal/mol value, almost 9 kcal/mol higher than the experimental value. Clearly, DLPNO-CCSD(T) overestimates Δ*G* values for this series, though it gives very consistent results with the DFT method for other studied systems, described later. At this point we cannot provide any explanation of this discrepancy.

### Second generation Grubbs catalyst

Second generation Grubbs complexes featuring either SIMes (1,3-bis(2,4,6-trimethylphenyl)-4,5-dihydroimidazol-2-ylidene) or IMes (1,3-bis(2,4,6-trimethylphenyl)imidazol-2-ylidene) ligands are another class of important ruthenium-based metathesis catalysts, where the initiation relies on phosphine dissociation. The experimental values for PCy_3_ dissociation for these catalysts are 23.0 ± 0.4 and 24 ± 1 kcal/mol for SIMes-containing and IMes-containing systems, respectively [57]. Recently Grubbs synthesized and described also a novel metathesis catalyst featuring a labile carbodicarbene ligand replacing PCy_3_ [48]. Inspired by these results we decided to design similar systems with either SIMes or IMes and cationic carbenes.

**Scheme 4:**
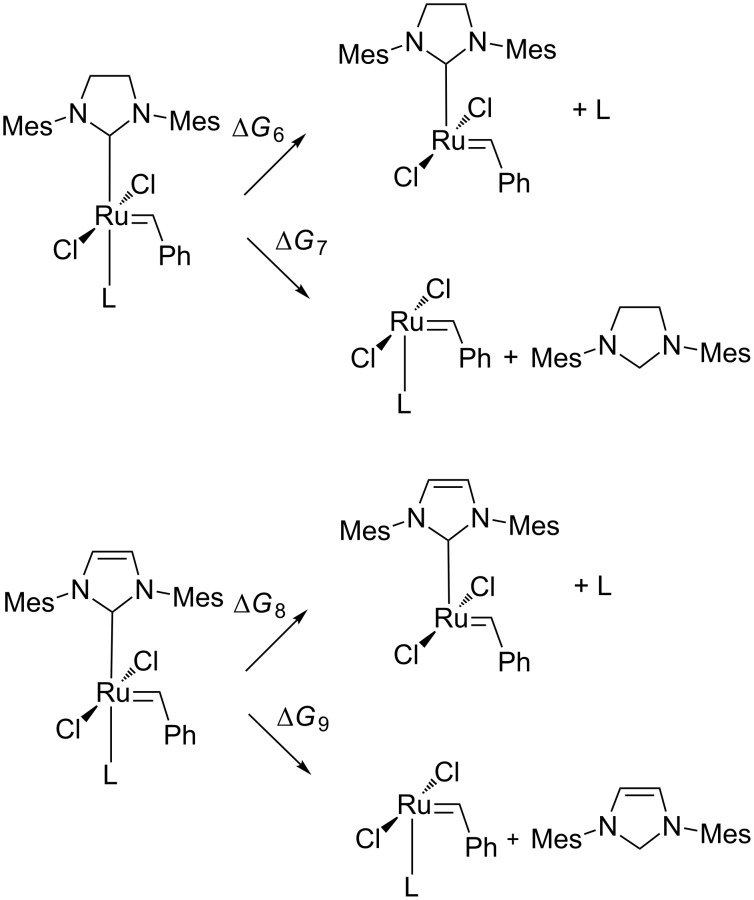
Dissociative mechanism of initiation of 2nd generation Grubbs-like saturated **1**–**3-GrII** and unsaturated **1**–**3-GrII_IMes** complexes; L = carbene **1**, **2** or **3**.

For all systems **1**–**3-GrII** and **1**–**3-GrII_IMes** the energy barriers of initiation are relatively high (30–40 kcal/mol, Scheme 4), indicating that these complexes are completely unsuitable for olefin metathesis. Precatalysts with unsaturated NHC ligands are estimated to have slightly lower Gibbs free energy barriers than saturated ones by ca. 3–5 kcal/mol (Table 3). Interestingly, the free energies in water are 3–12 kcal/mol lower indicating that **1-GrII** and **2-GrII** may act as very slow metathesis catalysts (see Supporting Information File 1).

**Table 3:** The comparison of Gibbs free energies ∆*G*_6_–∆*G*_9_ and structural parameters of investigated compounds.

complex	∆*G*_6_ [kcal/mol]	∆*G*_7_ [kcal/mol]	Ru–C [Å]	Ru–C_carbene_ [Å]

**1-GrII**	38.0	38.5	2.118	2.109
**2-GrII**	35.5	37.0	2.134	2.078
**3-GrII**	–	40.1	2.115	2.098

complex	∆*G*_8_ [kcal/mol]	∆*G*_9_ [kcal/mol]	Ru–C [Å]	Ru–C_carbene_ [Å]

**1-GrII_IMes**	33.3	35.5	2.130	2.102
**2-GrII_IMes**	30.9	34.1	2.141	2.066
**3-GrII_IMes**	–	36.7	2.122	2.079

### Hoveyda–Grubbs catalysts

**Scheme 5:**
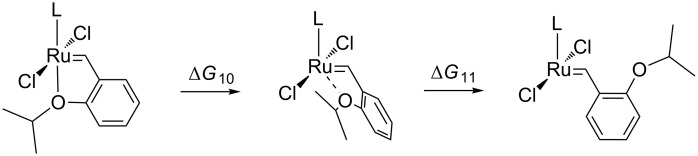
Dissociative mechanism of activation for complexes **1**–**3-Hov**; L = carbene **1**, **2** or **3**.

In the last step of our study we also designed Hoveyda-like precatalyst **1**–**3-Hov** with new cationic carbenes replacing SIMes (see Scheme 1). In our investigation we only considered the dissociative mechanism, which was shown to be the most feasible for medium and large-sized olefins (Scheme 5) [61–62]. Results presented in Table 4 suggest that the incorporation of cationic NHC increases the Gibbs free energy (**∆***G*_10_) barriers by ca. 4–6 kcal/mol with respect to the standard Hoveyda–Grubbs catalyst (**Hov**) [63]. Given the accuracy of our computational methods, estimated at around 1–2 kcal/mol, we can expect that cationic Hoveyda-type catalysts are only slightly slower than the Hoveyda–Grubbs catalyst. This result is in agreement with experimental reports on various onium tag-modified systems [23–24] showing moderate activities of these systems in model CM reactions. For this group of catalysts the results in water are virtually identical to those in CH_2_Cl_2_ (see Supporting Information File 1).

**Table 4:** The comparison of Gibbs free energies ∆*G*_10_ and ∆*G*_11_ and selected structural parameters of **Hov** and **1**–**3-Hov** catalysts.

complex	∆*G*_10_ [kcal/mol]	∆*G*_11_ [kcal/mol]	Ru–C_carbene_ [Å]	Ru–C_Ar_ [Å]	Ru–O1 [Å]

**1-Hov**	24.5	14.2	1.944	1.839	2.299
**2-Hov**	26.3	15.3	1.930	1.842	2.296
**3-Hov**	24.9	16.4	1.925	1.843	2.277
**Hov**^a^ X-ray	19–20	–	1.979(1)	1.829(1)	2.256(1)

^a^See ref. [64].

Surprisingly the differences in Δ*G*s for **1–3-Hov** as well as all other candidates for catalysts are relatively small and close to the computational accuracy of our protocol. To justify the lack of influence of the position of the quaternary amine on the Gibbs free energies of initiation we decided to perform a detailed analysis of partial charges of these systems, as well as complexes **1**–**3-GrI** (Table 5). Interestingly both natural partial charges and Mulliken partial charges (Table S8 in Supporting Information File 1) show no meaningful differences for the C_carbene_ atom. This result has important consequences concerning the strength of the Ru–C_carbene_ bond which is at least partially driven by the electrostatic interaction between Ru and C_carbene_ atoms. As a result the similar partial charge of C_carbene_ in **1**–**3-Hov** translates into similar bond strength of the Ru–C_carbene_ bond. This, in turn, has an impact on the Ru–O1 bond strengths due to the well-known *trans* effect which shows that there is a balance between the strength/bond length of the opposite bonds of the ruthenium center [38–41,65]. As a result the Ru–O1 bond strength in **1**–**3-Hov** is very similar, resulting in similar Gibbs free energies of initiation. The same argument can be made for **1**–**3-GrI** which also shows very similar natural partial charges on C_carbene_ atoms, resulting in very similar rates of initiation. It is interesting to note that the excess positive charge is located mostly on the –CH_2_N(CH_3_)_3_^+^ group in the case of **1-GrI** and **1-Hov**, but in the case of **2**–**3-GrI** and **2**–**3-Hov** it gets distributed over the imidazole core and mesityl groups. A similar feature has been observed by us earlier in carbene dimers formation, where mesityl groups, which usually act as weakly electron-donating moieties, could also accommodate a substantial amount of excess negative or positive charge [49].

**Table 5:** Natural partial charges distribution in carbenes of **1**–**3-Hov** and **1**–**3-GrI**.

atom	**1-Hov**	**2-Hov**	**3-Hov**	**Hov**	**1-GrI**	**2-GrI**	**3-GrI**	**GrII**

C_carbene_	0.49	0.46	0.47	0.49	0.41	0.38	0.40	0.41
N1	−0.51	−0.53	−0.32	−0.49	−0.50	−0.52	−0.31	−0.48
N2	−0.48	−0.48	−0.50	−0.49	−0.47	−0.47	−0.49	−0.48
C1	0.15	0.42	–	0.22	0.15	0.42	–	0.22
C2	0.24	0.25	0.49	0.22	0.24	0.25	0.49	0.22
Mes1	0.24	0.27	0.31	0.23	0.24	0.28	0.32	0.24
Mes2	0.26	0.27	0.28	0.24	0.25	0.27	0.28	0.22
N*	0.94	0.55	0.51	–	0.94	0.54	0.50	–
Ru1	0.31	0.33	0.32	0.31	0.14	0.15	0.13	0.14

## Conclusion

Despite hundreds of examples of ruthenium-based olefin metathesis catalysts synthesized up to date the rational design of new catalysts remains a non-trivial task. To gain general insight into the structure–activity relationship for this class of compounds we computationally investigated three different carbenes bearing a formal +1 charge, in form of quaternary amine, and their impact on the activation rates of olefin metathesis catalysts. We predict that these carbenes are likely to dimerize, similarly to other asymmetric carbenes synthesized earlier. We also demonstrate that most of the examined complexes, derivatives of Grubbs and Hoveyda–Grubbs catalysts **1**–**3-GrI**, **1**–**3-Ind** and **1**–**3-Hov** have initiation Gibbs free energy values in the range of standard metathesis catalysts, like **GrI**, **Ind** and Hoveyda–Grubbs and are likely an interesting alternative for them. On the other hand ruthenium complexes with two carbenes are predicted to have relatively high initiation energies. Our partial charges analysis reveals that the location of the quaternary amine and its distance from the carbene carbon atom has little influence on the electronic features of the crucial parts of the catalyst and, therefore, little influence on the initiation rates of catalyst bearing these moieties. The excess positive charge of the quaternary ammonium is, in most cases, distributed over the imidazole core and mesityl groups and does not affect the ruthenium core nor the ruthenium–C_carbene_ bond.

## Experimental

We used density functional theory (DFT) using a computational protocol similar to our previous studies. We have used all-atom models for all studied catalysts. Starting models for carbenes and precatalyst were prepared on the basis of available CSD crystal structures of a Grubbs and Hoveyda–Grubbs precatalysts (refcodes: ABEJUM01, GUBQUP, ZETLOZ and LOVPAP) [58–59,64,66]. In the geometry optimization step we used the M06 density functional with the 6-31G** basis set for C, N, O, Cl and H atoms, while the Ru atom, which was described by the Los Alamos angular momentum projected effective core potential (ECP) using the double-ζ contraction of valence functions (denoted as LACVP**). The choice of the M06 functional was made due to its very good performance in accurate description of ruthenium-based catalysts, giving accurate energies for a number of Grubbs and Hoveyda systems [31,67]. Since the M06 functional has already medium-range dispersion implemented, M06-D3 may overestimate the effect of dispersion due to double-counting of these effects [47]. On the other hand the addition of D3 correction to M06 was shown to improve the results for many organic reactions when calculating the differences in relative energies, therefore we decided to use it in this investigation [31,67–68]. To assess the need to use the D3 correction we have performed additional benchmark calculations for selected ruthenium catalysts and compared them with the experimental data. Based on these results we decided to use the D3 correction in the estimation of all Gibbs free energies apart from the Grubbs-like systems, where the D3 correction was omitted.

In all calculations we have used the standard energy convergence criterion of 5 × 10^−5^ Hartree. For each structure frequencies were calculated to verify the nature of each stationary point (zero imaginary frequencies for minima and one imaginary frequency for transition states). In the second step we performed solvation energy calculations using the Poisson–Boltzmann self-consistent polarizable continuum method as implemented in Jaguar v.7.9 (Schrodinger, 2013) to represent dichloromethane, using the dielectric constant of 8.93 and the effective radius 2.33 Å. The solvation calculations were performed using the M06-D3/LACVP** level of theory and the gas-phase optimized structures. We also used the same polarizable continuum method to estimate the solvation energies in water (dielectric constant of 80.73 and the effective radius 1.40 Å) and these results are presented in Supporting Information File 1. For all stationary points we have also performed single-point energy calculations with the valence polarized basis set denoted as LACV3P++**. Free energies discussed in this work for stationary points are calculated as the sum of electronic energy (from single-point LACV3P++** calculations), solvation energy, zero-point energy correction, thermal correction to enthalpy, and the negative product of temperature and entropy (at 298 K). All final estimates of Gibbs free energies include the counterpoise correction [69].

To further validate our results we used the very accurate, single-point DLPNO-CCSD(T) calculations using the DFT-optimized geometries and the def2-svp basis set using Orca v4.0.0.1 program [70–71]. Complete DLPNO-CCSD(T) results are presented in Supporting Information File 1.

## Supporting Information

File 1 Mulliken partial charges, energy values and Cartesian coordinates for all investigated systems.
