# Supplementary material for: The influence of the cationic carbenes on the initiation kinetics of ruthenium-based metathesis catalysts; a DFT study
Source: Beilstein J Org Chem. 2018 Nov 20;14:2872–80. doi: 10.3762/bjoc.14.266 (PMC6278762; doi:10.3762/bjoc.14.266)
Supplement: File 1 — Mulliken partial charges, energy values and Cartesian coordinates for all investigated systems. [file Beilstein_J_Org_Chem-14-2872-s001.pdf]

BEILSTEIN JOURNAL OF ORGANIC CHEMISTRY

Supporting Information

for

The influence of the cationic carbenes on the initiation kinetics of ruthenium-based metathesis catalysts; a DFT study

Magdalena Jawiczuk, Angelika Janaszkiewicz and Bartosz Trzaskowski

Beilstein J. Org. Chem. **2018,** *14,* 2872–2880. doi:10.3762/bjoc.14.266

Mulliken partial charges, energy values and Cartesian coordinates for all investigated systems

License and Terms: This is a supporting information file under the terms of the Creative Commons Attribution License (http://creativecommons.org/licenses/by/4.0). Please note that the reuse, redistribution and reproduction in particular requires that the authors and source are credited.
The license is subject to the *Beilstein Journal of Organic Chemistry* terms and conditions: (https://www.beilstein-journals.org/bjoc)

Contents

Contents....S2

Table S1. Total energy values (E) and Gibbs free energy values (G; as defined in the manuscript) for carbene **1** and its complexes. S2

Table S2. Total energy values (E) and Gibbs free energy values (G; as defined in the manuscript) for carbene **2** and its complexes. S3

Table S3. Total energy values (E) and Gibbs free energy values (G; as defined in the manuscript) for carbene **3** and its complexes. S3

Table S4. Calculated Gibbs free energies of dimerization (ΔG_1) for carbenes **1-2** using different computational approaches....S4

Table S5. Calculated Gibbs free energies of activation (ΔG_2-ΔG_5) for complexes **1-3-GrI** and **1-3-Ind** using different computational approaches. S4

Table S6. Calculated Gibbs free energies of activation (ΔG_6-ΔG_9) for complexes **1-3-GrII** and **1-3-GrII_IMES** using different computational approaches. S5

Table S7. Calculated Gibbs free energies of activation (ΔG_{10}-ΔG_{11}) for complexes **1-3-Hov** using different computational approaches....S5

Table S8. Mulliken partial charges of carbenes in **1-3-Hov** and **1-3-GrI** systems....S6

Cartesian coordinates S6

Table S1: Total energy values (E) and Gibbs free energy values (G; as defined in the manuscript) for carbene **1** and its complexes.

structure	M06 E, (H)	DLPNO-CCSD(T) E (H)	Solvatation E in CH_2Cl_2 (H)	Zero-point E (kcal/mol)	Entropy E (cal/mol)	Thermal correction to enthalpy (H)	M06 G (H)	DLPNO-CCSD(T) G (H)	Solvatation E in H_2O (H)	D3 correction (H)
**1**	-1137.763	-1134.209	-0.068	352.339	198.779	0.031	-1137.332	-1133.778	-0.084	-0.016
**1A**	-2275.555	-2268.439	-0.168	708.671	320.706	0.061	-2274.685	-2267.570	-0.196	-0.053
**1B**	-2275.554	-2268.438	-0.168	708.631	320.249	0.061	-2274.684	-2267.568	-0.196	-0.053
**1-GrI**	-3469.236	-3461.247	-0.067	725.605	329.792	0.062	-3468.241	-3460.252	-0.085	-0.053
**1-Ind**	-3776.339	-3767.400	-0.067	784.641	352.794	0.068	-3775.256	-3766.317	-0.085	-0.060
**1-GrII**	-3347.361	-3339.302	-0.069	686.752	345.480	0.066	-3346.434	-3338.375	-0.088	-0.055
**1-GrII_IMes**	-3346.156	-3338.112	-0.070	672.200	350,931	0.067	-3345.255	-3337.212	-0.090	-0.054
**1-Hov**	-2615.468	-2609.709	-0.076	478.132	263.670	0.047	-2614.860	-2609.102	-0.095	-0.031
**1-Hov-TS**	-2615.425	-2609.669	-0.077	477.292	267.828	0.047	-2614.821	-2609.065	-0.098	-0.029
**1-Hov-a**	-2615.441	-2609.675	-0.079	477.856	264.539	0.046	-2614.838	-2609.072	-0.101	-0.030
**P(Cy)$_3$**	-1046.796	-1043.971	0.003	301.675	142.238	0.020	-1046.359	-1043.534	-0.005	-0.010
**1-Gr_I_P(Cy)$_3$**	-2422.341	-2417.178	-0.086	422.246	244.819	0.042	-2421.829	-2416.666	-0.110	-0.028
**1-Ind_P(Cy)$_3$**	-2729.452	-2723.339	-0.079	481.062	271.524	0.048	-2728.846	-2722.733	-0.103	-0.034
**1-GrII_Imes/SIMes**	-2422.347	-2417.188	-0.079	422.208	243.879	0.041	-2421.828	-2416.668	-0.101	-0.026
**GrI_NC**	-2331.367	-2326.935	-0.00796	371.465	198.937	0.032	-2330.846	-2326.414	-0.016	-0.019
**GrII_NC_nas**	-2209.493	-2204.998	-0.003	332.325	216.117	0.035	-2209.034	-2204.539	-0.024	-0.019
**GrII_NC_nnas**	-2208.286	-2203.802	-0.014	317.811	219.853	0.035	-2207.863	-2203.379	-0.024	-0.019
**Ind_NC**	-2638.464	-2633.080	-0.010	430.406	219.452	0.037	-2637.855	-2632.471	-0.019	-0.024
**IMes**	-923.697	-920.815	-0.005	247.943	170.480	0.025	-923.364	-920.482	-0.009	-0.009
**SIMes**	-924.900	-922.008	0.002	262.277	173.288	0.025	-924.537	-921.644	-0.009	-0.010

Table S2: Total energy values (E) and Gibbs free energy values (G; as defined in the manuscript) for carbene **2** and its complexes.

structure	M06 E, (H)	DLPNO-CCSD(T) E (H)	Solvatation E in CH_2Cl_2 (H)	Zero-point E (kcal/mol)	Entropy E (cal/mol)	Thermal correction to enthalpy (H)	M06 G (H)	DLPNO-CCSD(T) G (H)	Solvatation E in H_2O (H)	D3 correction (H)
**2**	-980.606	-977.550	-0.080	281.272	180.295	0.027	-977.259	-977.241	-0.098	-0.011
**2A**	-1961.243	-1955.123	-0.197	567.095	276.630	0.051	-1960.617	-1954.497	-0.232	-0.042
**2B**	-3312.095	-1955.120	-0.200	566.868	270.669	0.050	-1960.617	-1954.495	-0.234	-0.042
**2-GrI**	-3312.095	-3304.602	-0.065	655.235	309.596	0.058	-3311.205	-3303.712	-0.082	-0.048
**2-Ind**	-3619.199	-3610.754	-0.064	714.437	323.197	0.062	-3618.216	-3609.771	-0.082	-0.055
**2-GrII**	-3190.207	-3182.645	-0.078	616.059	321.237	0.061	-3189.395	-3181.834	-0.100	-0.050
**2-GrII_IMes**	-3189.002	-3181.453	-0.079	601.417	328.031	0.062	-3188.217	-3180.667	-0.100	-0.049
**2-Hov**	-2458.324	-2453.062	-0.073	407.764	245.470	0.042	-2457.822	-2452.560	-0.091	-0.026
**2-Hov-TS**	-2458.280	-2453.020	-0.075	407.075	242.972	0.042	-2457.780	-2452.520	-0.097	-0.024
**2-Hov-a**	-2458.297	-2453.023	-0.078	407.597	239.583	0.042	-2457.798	-2452.524	-0.100	-0.026
**2-Gr_I_P(Cy)$_3$**	-2265.200	-2260.535	-0.082	351.664	222.181	0.037	-2264.790	-2260.125	-0.106	-0.023
**2-Ind_P(Cy)$_3$**	-2572.309	-2566.690	-0.079	410.646	247.878	0.043	-2571.809	-2566.190	-0.103	-0.028
**2-GrII_Imes/SIMes**	-2265.201	-2260.538	-0.079	351.750	228.839	0.038	-2264.791	-2260.128	-0.100	-0.021
**P(Cy)$_3$**	-1046.796	-1043.971	0.003	301.675	142.238	0.020	-1046.359	-1043.534	-0.005	-0.010
**GrI_NC**	-2331.367	-2326.935	-0.008	371.465	198.937	0.032	-2330.846	-2326.414	-0.016	-0.019
**GrII_NC_nas**	-2209.493	-980.606	-0.003	332.325	216.117	0.035	-2209.034	-2204.539	-0.024	-0.019
**GrII_NC_nnas**	-2208.286	-980.606	-0.014	317.811	219.853	0.035	-2207.863	-2203.379	-0.024	-0.019
**Ind_NC**	-2638.464	-980.606	-0.010	430.406	219.452	0.037	-2637.855	-2632.471	-0.019	-0.024
**IMes**	-923.697	-980.606	-0.005	247.943	170.480	0.025	-923.364	-920.482	-0.009	-0.009
**SIMes**	-924.900	-980.606	0.002	262.277	173.288	0.025	-924.537	-921.644	-0.009	-0.010

Table S3: Total energy values (E) and Gibbs free energy values (G; as defined in the manuscript) for carbene **3** and its complexes.

structure	M06 Gas Phase, LACV3P **++ (H)	DLPNO-CCSD(T) Gas Phase, def2-SVP (H)	Solvatation E in CH_2Cl_2 (H)	Zero-point E (kcal/mol)	Entropy E (cal/mol)	Thermal correction to enthalpy (H)	M06 G (H)	DLPNO-CCSD(T) G (H)	Solvatation E in H_2O (H)	D3 correction (H)
**3A**	-2039.715	-2033.350	-0.171	601.078	281.659	0.053	-2039.010	-2032.645	-0.197	-0.045
**3B**	-2039.717	-2033.352	-0.171	600.644	285.535	0.053	-2039.013	-2032.648	-0.197	-0.046
**3-GrI**	-3351.326	-3343.709	-0.058	671.738	307.293	0.058	-3350.402	-3342.785	-0.074	-0.049
**3-GrI_P(Cy)$_3$**	-2304.436	-2299.650	-0.069	367.745	231.936	0.038	-2303.991	-2299.204	-0.088	-0.023
**3-Ind**	-3658.437	-3649.864	-0.054	730.091	334.512	0.064	-3657.422	-3648.850	-0.069	-0.057
**3-Ind_P(Cy)$_3$**	-2611.542	-2605.797	-0.068	426.682	255.519	0.045	-2611.006	-2605.262	-0.088	-0.030
**3-GrII**	-3229.457	-3221.770	-0.058	632.294	325.449	0.062	-3228.600	-3220.913	-0.074	-0.051
**3-GrII_IMes/SIMes**	-2304.436	-2299.649	-0.069	367.710	232.139	0.039	-2303.991	-2299.204	-0.088	-0.023
**3-GrIIdim**	-3228.253	-3220.578	-0.058	617.813	331.365	0.063	-3227.421	-3219.747	-0.074	-0.051
**3-Hov**	-2497.560	-2492.172	-0.065	423.666	253.732	0.044	-2497.026	-2491.638	-0.081	-0.028
**3-Hov-TS**	-2497.518	-2492.131	-0.067	422.791	246.817	0.043	-2496.986	-2491.599	-0.087	-0.027
**3-Hov-a**	-2497.531	-2492.135	-0.069	423.545	249.760	0.043	-2497.000	-2491.604	-0.088	-0.027
**P(Cy)$_3$**	-1046.796	-1043.971	0.003	301.675	142.238	0.020	-1046.359	-1043.534	-0.005	-0.010
**GrI_NC**	-2331.367	-2326.935	-0.008	371.465	198.937	0.032	-2330.846	-2326.414	-0.016	-0.019
**GrII_NC_nas**	-2209.493	-2204.998	-0.003	332.325	216.117	0.035	-2209.034	-2204.539	-0.024	-0.019
**GrII_NC_nnas**	-2208.286	-2203.802	-0.014	317.811	219.853	0.035	-2207.863	-2203.379	-0.024	-0.019
**Ind_NC**	-2638.464	-980.606	-0.010	430.406	219.452	0.037	-2637.855	-2632.471	-0.019	-0.024
**IMes**	-923.697	-980.606	-0.005	247.943	170.480	0.025	-923.364	-920.482	-0.009	-0.009
**SIMes**	-924.900	-980.606	0.002	262.277	173.288	0.025	-924.537	-921.644	-0.009	-0.010

Table S4: Calculated Gibbs free energies of dimerization (ΔG_1) for carbenes **1-2** using different computational approaches.

**structure**	**ΔG_1 [kcal/mol]**			
	M06 in CH_2Cl_2	DLPNO-CCSD(T) in CH_2Cl_2	M06 in water	DLPNO-CCSD(T) in water
**1A**	-9.4	-8.7	-6.8	-6.0
**1B**	-9.0	-7.7	-6.6	-5.3
**2A**	-10.3	-9.8	-9.5	-9.0
**2B**	-10.0	-8.7	-9.1	-7.8
**3A**	-	-	-	-
**3B**	-	-	-	-

Table S5: Calculated Gibbs free energies of activation (ΔG_2-ΔG_5) for complexes **1-3-GrI** and **1-3-Ind** using different computational approaches.

**structure**	**ΔG_2 [kcal/mol]**			
	M06 in CH_2Cl_2	DLPNO-CCSD(T) in CH_2Cl_2	M06 in water	DLPNO-CCSD(T) in water
**1-GrI**	20.9	32.5	11.6	23.2
**2-GrI**	23.3	33.1	13.7	23.5
**3-GrI**	18.7	28.8	11.4	21.6
	**ΔG_3 [kcal/mol]**			
	M06 in CH_2Cl_2	DLPNO-CCSD(T) in CH_2Cl_2	M06 in water	DLPNO-CCSD(T) in water
**1-GrI**	24.0	37.7	20.1	33.8
**2-GrI**	23.7	35.7	18.0	30.0
**3-GrI**	-	-	-	-
	**ΔG_4 [kcal/mol]**			
	M06 in CH_2Cl_2	DLPNO-CCSD(T) in CH_2Cl_2	M06 in water	DLPNO-CCSD(T) in water
**1-Ind**	18.7	31.1	9.4	21.8
**2-Ind**	16.7	29.6	7.1	20.0
**3-Ind**	21.9	33.9	13.0	25.0
	**ΔG_5 [kcal/mol]**			
	M06 in CH_2Cl_2	DLPNO-CCSD(T) in CH_2Cl_2	M06 in water	DLPNO-CCSD(T) in water
**1-Ind**	25.6	37.6	20.7	37.7
**2-Ind**	22.5	37.3	16.4	31.2
**3-Ind**	-	-	-	-

Table S6: Calculated Gibbs free energies of activation (ΔG_6-ΔG_9) for complexes **1-3-GrII** and **1-3-GrII_IMES** using different computational approaches.

structure	ΔG_6 [kcal/mol]			
	M06 in CH_2Cl_2	DLPNO-CCSD(T) in CH_2Cl_2	M06 in water	DLPNO-CCSD(T) in water
**1-GrII**	38.0	36.5	26.8	25.3
**2-GrII**	35.5	33.7	24.5	22.7
**3-GrII**	-	-	-	-
	ΔG_7 [kcal/mol]			
	M06 in CH_2Cl_2	DLPNO-CCSD(T) in CH_2Cl_2	M06 in water	DLPNO-CCSD(T) in water
**1-GrII**	38.5	39.2	28.1	29.8
**2-GrII**	37.0	38.5	29.6	31.4
**3-GrII**	40.1	40.7	31.1	31.7
	ΔG_8 [kcal/mol]			
	M06 in CH_2Cl_2	DLPNO-CCSD(T) in CH_2Cl_2	M06 in water	DLPNO-CCSD(T) in water
**1-GrII_IMES**	33.3	34.6	30.1	31.4
**2-GrII_IMES**	30.9	30.3	27.4	26.7
**3-GrII_IMES**	-	-	-	-
	ΔG_9 [kcal/mol]			
	M06 in CH_2Cl_2	DLPNO-CCSD(T) in CH_2Cl_2	M06 in water	DLPNO-CCSD(T) in water
**1-GrII_IMES**	35.5	38.7	31.3	34.5
**2-GrII_IMES**	34.1	36.3	31.8	33.9
**3-GrII_IMES**	36.7	38.4	32.5	34.2

Table S7: Calculated Gibbs free energies of activation (ΔG_{10}-ΔG_{11}) for complexes **1-3-Hov** using different computational approaches.

structure	ΔG_{10} [kcal/mol]			
	M06 in CH_2Cl_2	DLPNO-CCSD(T) in CH_2Cl_2	M06 in water	DLPNO-CCSD(T) in water
**1-GrII**	24.5	22.7	23.3	21.4
**2-GrII**	26.3	24.7	24.0	22.4
**3-GrII**	24.9	24.2	23.1	22.4
	ΔG_{11} [kcal/mol]			
	M06 in CH_2Cl_2	DLPNO-CCSD(T) in CH_2Cl_2	M06 in water	DLPNO-CCSD(T) in water
**1-GrII**	14.2	18.5	12.7	16.9
**2-GrII**	15.3	22.5	13.1	20.4
**3-GrII**	16.4	21.4	14.8	19.7

Table S8: Mulliken partial charges of carbenes in **1-3-Hov** and **1-3-GrI** systems.

Atom No.	1-Hov	2-Hov	3-Hov	Hov	1-GrI	2-GrI	3-GrI	GrII
$C_{carbene}$	0.43	0.38	0.41	0.44	0.44	0.40	0.44	0.45
N1	-0.50	-0.47	-0.35	-0.48	-0.50	-0.48	-0.36	-0.50
N2	-0.49	-0.49	-0.51	-0.50	-0.50	-0.50	-0.51	-0.49
C1	0.15	0.25	-	0.20	0.14	0.26	-	0.20
C2	0.22	0.28	0.45	0.20	0.22	0.28	0.46	0.20
N3	0.86	0.48	0.38	-	0.85	0.47	0.38	-
Mes1	0.31	0.35	0.42	0.27	0.32	0.34	0.40	0.26
Mes2	0.33	0.35	0.37	0.30	0.33	0.34	0.37	0.28

Cartesian coordinates

1	1A
N1 0.862 5.636 5.258<br>N2 1.082 4.866 7.249<br>C1 1.497 4.677 5.984<br>C2 0.128 6.625 6.089<br>C3 -0.007 5.852 7.389<br>C4 1.117 5.945 3.891<br>C5 2.428 6.223 3.460<br>C6 2.616 6.604 2.134<br>C7 1.556 6.714 1.234<br>C8 0.274 6.416 1.689<br>C9 0.033 6.020 3.004<br>C10 3.609 6.092 4.375<br>C11 1.799 7.103 -0.192<br>C12 -1.360 5.653 3.425<br>C13 1.369 3.986 8.337<br>C14 2.157 4.454 9.392<br>C15 2.396 3.603 10.468<br>C16 1.877 2.310 10.503<br>C17 1.097 1.876 9.431<br>C18 0.827 2.695 8.339<br>C19 2.756 5.830 9.357<br>C20 2.169 1.391 11.650<br>C21 -0.001 2.204 7.190<br>H3 3.627 6.819 1.789<br>H4 -0.564 6.469 0.994<br>H5 3.490 6.664 5.307<br>H6 4.521 6.441 3.882<br>H7 3.749 5.052 4.687<br>H8 2.628 7.812 -0.281<br>H9 0.911 7.556 -0.645<br>H10 2.060 6.226 -0.798<br>H11 -1.925 5.250 2.578<br>H12 -1.936 6.517 3.793<br>H13 -1.355 4.895 4.217<br>H14 3.013 3.953 11.295<br>H15 0.685 0.867 9.448<br>H16 3.241 6.023 8.392<br>H17 3.508 5.950 10.143<br>H18 2.003 6.619 9.514<br>H19 1.292 0.791 11.917<br>H20 2.488 1.943 12.540<br>H21 2.973 0.689 11.395	N1 0.964 5.524 4.980<br>N2 0.910 4.680 7.097<br>C1 1.508 4.475 5.798<br>C2 0.507 6.577 5.876<br>C3 -0.042 5.784 7.029<br>C4 1.482 6.103 3.763<br>C5 2.761 6.675 3.669<br>C6 3.120 7.316 2.484<br>C7 2.244 7.439 1.410<br>C8 0.957 6.931 1.560<br>C9 0.556 6.275 2.719<br>C10 3.757 6.612 4.785<br>C11 2.674 8.065 0.120<br>C12 -0.862 5.800 2.834<br>C13 0.645 3.778 8.179<br>C14 0.915 4.258 9.481<br>C15 0.583 3.472 10.582<br>C16 -0.022 2.225 10.447<br>C17 -0.311 1.787 9.158<br>C18 0.003 2.533 8.020<br>C19 1.544 5.595 9.744<br>C20 -0.303 1.371 11.644<br>C21 -0.340 1.954 6.682<br>H3 4.124 7.737 2.402<br>H4 0.242 7.042 0.743<br>H5 3.403 7.115 5.695<br>H6 4.692 7.101 4.493<br>H7 3.979 5.575 5.062<br>H8 3.541 8.719 0.252<br>H9 1.869 8.653 -0.334<br>H10 2.955 7.291 -0.608<br>H11 -1.355 5.810 1.856<br>H12 -1.456 6.453 3.495<br>H13 -0.924 4.784 3.236<br>H14 0.822 3.845 11.580<br>H15 -0.794 0.818 9.024<br>H16 2.087 5.981 8.877<br>H17 2.244 5.530 10.584<br>H18 0.796 6.350 10.031<br>H19 -1.047 0.599 11.428<br>H20 -0.660 1.964 12.493<br>H21 0.612 0.862 11.974

H22 0.594 2.157 6.270	H22 0.549 1.862 6.051
H23 -0.850 2.869 6.980	H23 -1.050 2.564 6.109
H24 -0.402 1.207 7.393	H24 -0.787 0.961 6.796
H46 -0.846 6.846 5.636	H46 -0.232 7.208 5.368
C115 1.001 7.874 6.168	H47 1.329 7.231 6.219
H48 0.123 6.458 8.294	H48 -0.011 6.358 7.967
H49 -0.977 5.337 7.461	N49 3.047 2.579 6.361
N118 0.271 9.198 6.324	N50 2.927 3.169 4.164
H51 1.707 7.803 7.007	C51 2.411 3.513 5.462
H52 1.572 7.978 5.236	C52 4.005 1.766 5.625
C121 -0.549 9.216 7.573	C53 3.419 1.800 4.242
H122 -1.033 10.192 7.664	C54 3.334 2.639 7.763
H123 -1.307 8.432 7.521	C55 3.976 3.729 8.389
H124 0.108 9.044 8.430	C56 4.321 3.622 9.737
C125 1.302 10.281 6.400	C57 4.069 2.478 10.489
H126 0.801 11.244 6.519	C58 3.465 1.404 9.842
H127 1.954 10.091 7.257	C59 3.096 1.458 8.501
H128 1.888 10.275 5.478	C60 4.280 5.017 7.686
C129 -0.603 9.457 5.134	C61 4.379 2.419 11.952
H130 -0.981 10.480 5.195	C62 2.459 0.237 7.903
H131 -0.011 9.323 4.224	C63 2.389 3.421 2.848
H132 -1.442 8.760 5.132	C64 3.299 3.912 1.895
	C65 2.881 4.065 0.577
	C66 1.593 3.729 0.169
	C67 0.732 3.185 1.118
	C68 1.109 3.005 2.448
	C69 4.718 4.243 2.252
	C70 1.146 3.983 -1.236
	C71 0.133 2.387 3.400
	H72 4.800 4.476 10.219
	H73 3.251 0.494 10.405
	H74 5.013 4.915 6.874
	H75 4.686 5.751 8.390
	H76 3.383 5.440 7.225
	H77 5.152 3.140 12.236
	H78 4.711 1.421 12.258
	H79 3.483 2.651 12.542
	H80 1.773 -0.226 8.621
	H81 3.205 -0.535 7.656
	H82 1.899 0.460 6.991
	H83 3.584 4.466 -0.155
	H84 -0.272 2.885 0.815
	H85 4.785 4.800 3.192
	H86 5.185 4.844 1.466
	H87 5.330 3.333 2.362
	H88 0.306 3.341 -1.520
	H89 1.956 3.823 -1.955
	H90 0.816 5.024 -1.350
	H91 -0.080 3.057 4.240
	H92 0.503 1.446 3.830
	H93 -0.809 2.158 2.891
	H94 4.016 0.747 6.037
	C95 5.405 2.394 5.688
	H96 4.142 1.615 3.439
	H97 2.612 1.051 4.148
	N98 6.565 1.417 5.783
	H99 5.586 3.024 4.807
	H100 5.500 3.005 6.589
	C101 6.544 0.432 4.660
	H102 7.419 -0.219 4.742
	H103 5.635 -0.171 4.715
	H104 6.577 0.977 3.712
	C105 7.834 2.208 5.711
	H106 8.686 1.530 5.809
	H107 7.877 2.725 4.749

	H108 7.843 2.936 6.526
	C109 6.528 0.700 7.099
	H110 7.419 0.073 7.181
	H111 6.506 1.439 7.905
	H112 5.635 0.076 7.156
	C113 -1.457 5.254 6.758
	H114 -1.552 4.231 7.135
	H115 -1.668 5.258 5.680
	N116 -2.591 6.002 7.439
	C117 -2.517 5.804 8.923
	H118 -3.392 6.271 9.382
	H119 -2.500 4.732 9.138
	H120 -1.609 6.267 9.313
	C121 -2.562 7.459 7.112
	H122 -3.421 7.945 7.584
	H123 -1.639 7.904 7.490
	H124 -2.619 7.579 6.026
	C125 -3.880 5.420 6.947
	H126 -4.714 5.919 7.448
	H127 -3.949 5.573 5.867
	H128 -3.895 4.351 7.173
**1B**	**1-GrI**
N1 1.013 5.618 5.203	Ru1 3.235 3.277 5.598
N2 0.986 4.781 7.321	Cl1 1.423 1.653 5.340
C1 1.539 4.547 6.011	Cl2 5.049 4.766 6.334
C2 0.196 6.504 6.022	P1 4.845 1.412 5.528
C3 -0.219 5.580 7.134	N1 1.638 5.917 5.564
C4 1.490 6.245 4.008	N2 1.373 4.830 7.441
C5 2.797 6.751 3.861	C1 1.946 4.752 6.222
C6 3.118 7.466 2.707	C2 0.660 6.718 6.310
C7 2.191 7.714 1.698	C3 0.794 6.139 7.710
C8 0.897 7.233 1.881	C4 1.722 6.122 4.149
C9 0.527 6.504 3.009	C5 2.705 6.972 3.624
C10 3.874 6.524 4.879	C6 2.672 7.251 2.253
C11 2.575 8.417 0.434	C7 1.716 6.691 1.411
C12 -0.892 6.025 3.106	C8 0.764 5.830 1.964
C13 0.799 3.853 8.411	C9 0.747 5.529 3.321
C14 1.178 4.314 9.686	C10 3.801 7.569 4.460
C15 0.953 3.503 10.793	C11 1.734 6.940 -0.065
C16 0.336 2.258 10.680	C12 -0.263 4.565 3.869
C17 -0.106 1.866 9.421	C13 1.449 3.884 8.518
C18 0.097 2.645 8.281	C14 2.457 4.002 9.482
C19 1.783 5.671 9.896	C15 2.430 3.112 10.557
C20 0.189 1.369 11.875	C16 1.435 2.149 10.695
C21 -0.434 2.151 6.972	C17 0.418 2.099 9.739
H3 4.140 7.832 2.589	C18 0.391 2.968 8.655
H4 0.150 7.406 1.104	C19 3.498 5.080 9.441
H5 3.660 6.981 5.855	C20 1.444 1.181 11.839
H6 4.824 6.943 4.531	C21 -0.753 2.946 7.688
H7 4.011 5.456 5.071	C22 6.606 1.911 5.190
H8 3.505 8.984 0.548	C23 7.635 0.781 5.295
H9 1.793 9.106 0.096	C24 9.050 1.356 5.262
H10 2.731 7.691 -0.375	C25 9.280 2.195 4.009
H11 -1.254 5.706 2.123	C26 8.232 3.296 3.883
H12 -1.574 6.823 3.439	C27 6.820 2.717 3.905
H13 -1.000 5.184 3.798	C28 4.940 0.649 7.226
H14 1.274 3.851 11.776	C29 5.626 1.523 8.280
H15 -0.633 0.917 9.315	C30 5.811 0.718 9.565
H16 2.560 5.899 9.162	C31 4.484 0.179 10.088
H17 2.224 5.747 10.895	C32 3.721 -0.601 9.022
H18 1.022 6.466 9.826	C33 3.563 0.214 7.742
H19 -0.535 0.567 11.699	C34 4.346 -0.006 4.417
H20 -0.130 1.928 12.761	C35 4.792 -1.414 4.829
H21 1.150 0.898 12.124	C36 4.128 -2.454 3.926

H22 0.368 2.012 6.239	C37 4.419 -2.196 2.451
H23 -1.155 2.846 6.522	C38 4.022 -0.779 2.052
H24 -0.950 1.194 7.102	C39 4.712 0.244 2.949
H46 -0.673 6.851 5.442	C40 3.578 3.851 3.881
C115 1.032 7.691 6.522	C41 3.177 3.450 2.539
H48 -0.485 6.087 8.070	C42 2.151 2.539 2.232
H49 -1.087 4.972 6.820	C43 1.867 2.224 0.912
N118 0.317 9.030 6.587	C44 2.612 2.783 -0.126
H51 1.427 7.489 7.527	C45 3.640 3.677 0.160
H52 1.867 7.873 5.839	C46 3.909 4.017 1.478
C121 -0.919 8.949 7.422	H3 3.441 7.901 1.832
H122 -1.374 9.941 7.480	H4 0.032 5.354 1.309
H123 -1.624 8.250 6.968	H5 3.703 8.664 4.532
H124 -0.645 8.608 8.425	H6 4.775 7.383 3.993
C125 1.257 10.020 7.205	H7 3.854 7.131 5.461
H126 0.775 11.000 7.237	H8 2.330 7.821 -0.323
H127 1.503 9.693 8.218	H9 0.724 7.075 -0.467
H128 2.165 10.071 6.599	H10 2.172 6.076 -0.583
C129 -0.022 9.501 5.205	H11 -0.885 4.160 3.064
H130 -0.451 10.504 5.273	H12 -0.943 5.021 4.605
H131 0.891 9.517 4.602	H13 0.223 3.716 4.373
H132 -0.745 8.823 4.749	H14 3.213 3.185 11.314
N65 3.040 2.587 6.442	H15 -0.387 1.372 9.853
N66 2.824 3.239 4.269	H16 3.701 5.442 8.428
C67 2.380 3.557 5.604	H17 4.450 4.714 9.840
C68 3.889 1.726 5.629	H18 3.194 5.927 10.074
C69 3.224 1.838 4.285	H19 0.449 1.084 12.289
C70 3.418 2.598 7.825	H20 2.147 1.484 12.621
C71 4.153 3.642 8.422	H21 1.740 0.179 11.501
C72 4.587 3.495 9.739	H22 -0.427 2.570 6.710
C73 4.335 2.346 10.485	H23 -1.175 3.950 7.532
C74 3.638 1.315 9.863	H24 -1.556 2.300 8.054
C75 3.172 1.416 8.554	H25 6.782 2.623 6.014
C76 4.463 4.923 7.709	H26 7.504 0.084 4.450
C77 4.744 2.244 11.922	H27 7.492 0.194 6.213
C78 2.422 0.249 7.979	H28 9.196 1.986 6.156
C79 2.228 3.560 2.992	H29 9.788 0.546 5.327
C80 3.110 4.033 2.003	H30 10.291 2.622 4.014
C81 2.625 4.272 0.720	H31 9.222 1.541 3.124
C82 1.298 4.028 0.379	H32 8.387 3.873 2.962
C83 0.466 3.487 1.355	H33 8.334 4.006 4.720
C84 0.905 3.231 2.653	H34 6.086 3.529 3.848
C85 4.569 4.253 2.274	H35 6.674 2.079 3.020
C86 0.779 4.370 -0.983	H36 5.555 -0.258 7.101
C87 -0.053 2.622 3.629	H37 5.010 2.419 8.471
H88 5.134 4.318 10.202	H38 6.601 1.890 7.932
H89 3.421 0.403 10.424	H39 6.498 -0.120 9.360
H90 5.097 4.787 6.821	H40 6.299 1.338 10.330
H91 4.988 5.615 8.375	H41 3.864 1.032 10.403
H92 3.547 5.406 7.354	H42 4.644 -0.442 10.981
H93 5.606 2.881 12.147	H43 2.732 -0.901 9.397
H94 4.995 1.216 12.202	H44 4.260 -1.535 8.788
H95 3.923 2.565 12.578	H45 3.006 -0.356 6.987
H96 1.789 -0.211 8.746	H46 2.952 1.111 7.943
H97 3.101 -0.544 7.628	H47 3.245 0.033 4.489
H98 1.784 0.537 7.139	H48 5.888 -1.500 4.749
H99 3.307 4.663 -0.036	H49 4.533 -1.628 5.874
H100 -0.568 3.248 1.098	H50 4.455 -3.461 4.213
H101 4.744 4.794 3.208	H51 3.039 -2.420 4.093
H102 5.032 4.820 1.460	H52 5.497 -2.329 2.267
H103 5.115 3.298 2.343	H53 3.903 -2.935 1.824
H104 -0.079 3.749 -1.260	H54 2.929 -0.657 2.147
H105 1.549 4.251 -1.753	H55 4.268 -0.583 1.000
H106 0.448 5.417 -1.017	H56 4.444 1.261 2.638
H107 -0.171 3.247 4.520	H57 5.803 0.148 2.822

H108 0.274 1.634 3.977<br>H109 -1.038 2.489 3.169<br>H110 3.852 0.696 6.012<br>C111 5.330 2.256 5.616<br>H112 3.883 1.626 3.434<br>H113 2.363 1.149 4.226<br>N114 6.424 1.201 5.595<br>H115 5.493 2.906 4.746<br>H116 5.528 2.824 6.530<br>C117 6.251 0.254 4.452<br>H118 7.083 -0.455 4.451<br>H119 5.310 -0.288 4.561<br>H120 6.250 0.824 3.518<br>C122 7.736 1.906 5.444<br>H125 8.544 1.169 5.458<br>H129 7.742 2.447 4.494<br>H133 7.857 2.607 6.272<br>C126 6.435 0.448 6.891<br>H134 7.282 -0.242 6.888<br>H135 6.526 1.162 7.715<br>H136 5.505 -0.113 7.001	H58 4.310 4.676 3.849<br>H59 1.597 2.077 3.046<br>H60 1.071 1.517 0.689<br>H61 2.395 2.515 -1.158<br>H62 4.234 4.106 -0.645<br>H63 4.708 4.722 1.708<br>H114 -0.350 6.499 5.918<br>C115 0.941 8.217 6.253<br>H116 1.483 6.735 8.333<br>H117 -0.161 6.049 8.243<br>N118 -0.110 9.064 5.551<br>H119 1.011 8.642 7.262<br>H120 1.887 8.412 5.740<br>C121 -1.417 8.972 6.274<br>H122 -2.135 9.640 5.791<br>H123 -1.790 7.947 6.231<br>H124 -1.269 9.273 7.315<br>C125 0.364 10.483 5.588<br>H126 -0.385 11.120 5.109<br>H127 0.503 10.790 6.627<br>H128 1.311 10.552 5.047<br>C129 -0.298 8.672 4.114<br>H130 -1.022 9.361 3.669<br>H131 0.663 8.732 3.594<br>H132 -0.670 7.648 4.052
**1-Ind**	**1-GrII**
Ru1 6.489 8.792 11.240<br>Cl1 6.389 6.350 11.423<br>Cl2 7.227 11.142 11.289<br>P1 7.610 8.429 9.070<br>N1 5.126 9.820 13.840<br>N2 7.097 8.962 14.220<br>C1 6.170 9.147 13.257<br>C2 5.280 9.886 15.302<br>C3 6.776 9.673 15.451<br>C4 3.796 9.959 13.326<br>C5 3.332 11.222 12.920<br>C6 1.979 11.357 12.603<br>C7 1.093 10.282 12.667<br>C8 1.592 9.036 13.047<br>C9 2.933 8.849 13.377<br>C10 4.224 12.424 12.798<br>C11 -0.351 10.466 12.305<br>C12 3.424 7.484 13.762<br>C13 8.332 8.233 14.160<br>C14 9.540 8.916 13.986<br>C15 10.719 8.171 14.040<br>C16 10.715 6.798 14.266<br>C17 9.492 6.164 14.492<br>C18 8.291 6.862 14.469<br>C19 9.605 10.403 13.803<br>C20 11.986 6.005 14.263<br>C21 7.006 6.169 14.801<br>C22 7.665 9.956 7.991<br>C23 8.908 10.159 7.117<br>C24 8.862 11.539 6.458<br>C25 7.587 11.744 5.648<br>C26 6.350 11.495 6.505<br>C27 6.402 10.107 7.134<br>C28 9.402 8.040 9.404<br>C29 10.050 9.084 10.317<br>C30 11.562 8.875 10.353<br>C31 11.919 7.458 10.794<br>C32 11.188 6.397 9.977	N1 5.675 0.685 1.585<br>N2 5.167 0.661 3.696<br>C1 4.918 1.311 2.540<br>C2 6.624 -0.281 2.161<br>C3 6.014 -0.517 3.527<br>C4 5.926 1.069 0.234<br>C5 5.158 0.461 -0.777<br>C6 5.550 0.651 -2.097<br>C7 6.656 1.438 -2.431<br>C8 7.373 2.050 -1.406<br>C9 7.030 1.879 -0.062<br>C10 3.943 -0.353 -0.435<br>C11 7.017 1.649 -3.869<br>C12 7.840 2.553 1.008<br>C13 4.592 0.773 5.008<br>C14 3.457 0.017 5.323<br>C15 3.116 -0.112 6.672<br>C16 3.877 0.466 7.682<br>C17 4.996 1.222 7.327<br>C18 5.384 1.377 6.002<br>C19 2.627 -0.677 4.288<br>C20 3.539 0.277 9.130<br>C21 6.634 2.126 5.657<br>H3 4.957 0.202 -2.896<br>H4 8.219 2.693 -1.655<br>H5 4.185 -1.274 0.116<br>H6 3.403 -0.641 -1.342<br>H7 3.253 0.207 0.213<br>H8 7.091 0.699 -4.412<br>H9 7.967 2.182 -3.980<br>H10 6.238 2.240 -4.370<br>H11 8.174 3.539 0.664<br>H12 8.756 1.994 1.261<br>H13 7.269 2.723 1.929<br>H14 2.244 -0.714 6.933<br>H15 5.604 1.677 8.111<br>H16 1.761 -0.061 4.015<br>H17 2.260 -1.638 4.666

C33 9.680 6.639 9.961<br>C34 7.052 6.954 8.077<br>C35 7.898 6.654 6.834<br>C36 7.527 5.282 6.269<br>C37 6.037 5.189 5.957<br>C38 5.193 5.536 7.180<br>C39 5.561 6.913 7.727<br>H3 1.618 12.327 12.256<br>H4 0.923 8.174 13.058<br>H5 3.957 13.205 13.529<br>H6 4.092 12.877 11.808<br>H7 5.286 12.173 12.889<br>H8 -0.876 11.089 13.040<br>H9 -0.878 9.507 12.242<br>H10 -0.447 10.973 11.336<br>H11 2.681 6.724 13.497<br>H12 3.612 7.386 14.844<br>H13 4.359 7.221 13.248<br>H14 11.670 8.686 13.892<br>H15 9.477 5.095 14.711<br>H16 8.734 10.800 13.271<br>H17 10.494 10.681 13.226<br>H18 9.688 10.911 14.774<br>H19 12.031 5.314 15.113<br>H20 12.868 6.653 14.300<br>H21 12.062 5.397 13.352<br>H22 6.400 6.027 13.895<br>H23 6.404 6.744 15.520<br>H24 7.199 5.184 15.237<br>H25 7.652 10.763 8.744<br>H26 8.954 9.378 6.340<br>H27 9.830 10.074 7.706<br>H28 8.917 12.305 7.249<br>H29 9.749 11.680 5.827<br>H30 7.565 12.753 5.217<br>H31 7.581 11.041 4.798<br>H32 5.432 11.605 5.913<br>H33 6.298 12.251 7.307<br>H34 5.496 9.921 7.725<br>H35 6.418 9.355 6.328<br>H36 9.893 8.102 8.417<br>H37 9.633 8.965 11.333<br>H38 9.805 10.110 10.011<br>H39 11.972 9.070 9.348<br>H40 12.030 9.611 11.023<br>H41 11.625 7.343 11.846<br>H42 13.005 7.301 10.748<br>H43 11.403 5.396 10.377<br>H44 11.561 6.403 8.939<br>H45 9.188 5.859 9.365<br>H46 9.263 6.555 10.979<br>H47 7.208 6.144 8.810<br>H48 7.714 7.427 6.068<br>H49 8.973 6.684 7.059<br>H50 8.124 5.071 5.373<br>H51 7.795 4.512 7.013<br>H52 5.795 5.894 5.145<br>H53 5.786 4.187 5.583<br>H54 5.355 4.785 7.972<br>H55 4.123 5.507 6.932<br>H56 4.956 7.139 8.611<br>H57 5.322 7.675 6.971<br>H114 4.739 9.032 15.751<br>C115 4.803 11.202 15.907<br>H116 7.319 10.635 15.484	H18 3.165 -0.847 3.346<br>H19 4.421 -0.027 9.705<br>H20 2.761 -0.480 9.274<br>H21 3.177 1.213 9.577<br>H22 6.412 3.011 5.045<br>H23 7.335 1.517 5.066<br>H24 7.157 2.449 6.563<br>H114 7.601 0.227 2.265<br>C115 6.755 -1.538 1.307<br>H116 5.391 -1.429 3.550<br>H117 6.748 -0.587 4.340<br>N118 8.179 -1.964 0.996<br>H119 6.285 -2.401 1.796<br>H120 6.285 -1.390 0.328<br>C121 8.975 -2.129 2.251<br>H122 9.960 -2.526 1.994<br>H123 9.090 -1.162 2.745<br>H124 8.452 -2.826 2.911<br>C125 8.112 -3.282 0.290<br>H126 9.125 -3.593 0.022<br>H127 7.659 -4.022 0.954<br>H128 7.506 -3.168 -0.612<br>C129 8.843 -0.974 0.084<br>H130 9.841 -1.348 -0.160<br>H131 8.240 -0.866 -0.822<br>H132 8.918 -0.005 0.582<br>Ru1 3.713 3.030 2.335<br>Cl1 1.765 1.608 1.932<br>Cl2 5.624 4.372 3.126<br>N68 2.453 5.915 2.085<br>N69 2.112 4.993 4.023<br>C70 2.588 4.767 2.783<br>C71 1.726 6.953 2.836<br>C72 1.761 6.405 4.250<br>C73 2.587 6.117 0.678<br>C74 3.658 6.881 0.199<br>C75 3.710 7.150 -1.169<br>C76 2.723 6.701 -2.044<br>C77 1.660 5.963 -1.527<br>C78 1.567 5.661 -0.173<br>C79 4.729 7.383 1.119<br>C80 2.792 7.003 -3.510<br>C81 0.425 4.840 0.346<br>C82 1.824 4.060 5.069<br>C83 2.651 3.999 6.197<br>C84 2.210 3.253 7.291<br>C85 0.991 2.584 7.284<br>C86 0.196 2.662 6.141<br>C87 0.575 3.414 5.035<br>C88 3.936 4.764 6.291<br>C89 0.518 1.806 8.474<br>C90 -0.363 3.585 3.881<br>C40 4.184 3.538 0.623<br>C41 3.707 3.318 -0.739<br>C42 2.573 2.575 -1.111<br>C43 2.237 2.420 -2.447<br>C44 3.013 3.006 -3.447<br>C45 4.128 3.762 -3.099<br>C46 4.468 3.915 -1.762<br>H100 4.549 7.728 -1.560<br>H101 0.888 5.593 -2.204<br>H102 4.349 8.156 1.800<br>H103 5.550 7.830 0.548<br>H104 5.134 6.579 1.748<br>H105 3.804 7.288 -3.816

H117 7.053 9.082 16.333<br>N118 3.621 11.106 16.861<br>H119 5.599 11.674 16.498<br>H120 4.508 11.905 15.123<br>C121 3.995 10.302 18.066<br>H122 3.147 10.285 18.756<br>H123 4.238 9.281 17.764<br>H124 4.860 10.764 18.549<br>C125 3.282 12.499 17.292<br>H126 2.461 12.458 18.013<br>H127 4.160 12.960 17.752<br>H128 2.975 13.072 16.412<br>C129 2.404 10.513 16.215<br>H130 1.586 10.552 16.939<br>H131 2.157 11.090 15.318<br>H132 2.600 9.479 15.929<br>C120 4.768 8.968 10.551<br>C122 4.061 10.120 9.908<br>C123 4.451 11.415 9.605<br>H125 5.451 11.755 9.861<br>C124 3.544 12.274 8.970<br>H129 3.853 13.290 8.731<br>C126 2.279 11.834 8.613<br>H133 1.595 12.505 8.098<br>C128 1.880 10.522 8.894<br>H134 0.904 10.169 8.571<br>C130 2.763 9.682 9.551<br>C131 2.627 8.269 9.957<br>C132 3.794 7.878 10.534<br>H135 4.002 6.893 10.934<br>C40 1.459 7.414 9.781<br>C41 0.154 7.918 9.844<br>H136 -0.002 8.979 10.022<br>C42 -0.941 7.073 9.721<br>H138 -1.948 7.482 9.780<br>C43 -0.751 5.710 9.520<br>H140 -1.607 5.048 9.413<br>C44 0.541 5.195 9.450<br>H142 0.696 4.132 9.282<br>C45 1.636 6.037 9.581<br>H144 2.647 5.638 9.505	H106 2.121 7.828 -3.780<br>H107 2.488 6.133 -4.106<br>H108 -0.265 4.584 -0.465<br>H109 -0.151 5.372 1.116<br>H110 0.771 3.901 0.801<br>H111 2.836 3.223 8.186<br>H112 -0.775 2.163 6.132<br>H113 4.372 4.976 5.309<br>H115 4.675 4.195 6.871<br>H118 3.787 5.718 6.816<br>H121 -0.572 1.845 8.570<br>H125 0.954 2.184 9.406<br>H129 0.796 0.746 8.390<br>H133 0.017 3.082 2.983<br>H134 -0.504 4.645 3.628<br>H135 -1.346 3.164 4.116<br>H58 5.078 4.187 0.622<br>H59 1.963 2.125 -0.332<br>H60 1.355 1.841 -2.713<br>H61 2.740 2.884 -4.493<br>H62 4.725 4.248 -3.869<br>H63 5.338 4.514 -1.489<br>H136 0.704 7.048 2.437<br>H137 2.222 7.923 2.728<br>H138 2.537 6.881 4.867<br>H139 0.803 6.479 4.777
**1-GrIIdim**	**1-Hov**
N1 5.818 0.833 1.612<br>N2 5.391 0.815 3.744<br>C1 5.007 1.366 2.573<br>C2 6.872 -0.039 2.144<br>C3 6.514 -0.113 3.624<br>C4 5.866 1.078 0.207<br>C5 4.979 0.372 -0.629<br>C6 5.195 0.426 -2.003<br>C7 6.244 1.165 -2.558<br>C8 7.080 1.877 -1.702<br>C9 6.910 1.852 -0.316<br>C10 3.826 -0.404 -0.064<br>C11 6.418 1.222 -4.044<br>C12 7.822 2.660 0.562<br>C13 4.724 0.778 5.015<br>C14 3.665 -0.129 5.174<br>C15 3.178 -0.349 6.463<br>C16 3.736 0.276 7.575<br>C17 4.791 1.167 7.376<br>C18 5.314 1.424 6.112<br>C19 3.114 -0.895 4.011<br>C20 3.274 -0.034 8.967	Ru1 13.040 5.876 5.295<br>Cl1 11.008 5.211 6.387<br>Cl2 15.330 6.424 4.843<br>O1 13.823 3.783 5.835<br>C22 12.741 4.965 3.726<br>H27 12.253 5.376 2.836<br>C23 14.211 3.284 7.146<br>H28 15.136 2.705 6.999<br>C24 14.507 4.506 7.985<br>H29 15.269 5.136 7.512<br>H30 14.870 4.194 8.971<br>H31 13.593 5.099 8.135<br>C25 13.099 2.432 7.716<br>H32 12.872 1.566 7.085<br>H33 12.188 3.035 7.809<br>H34 13.386 2.064 8.707<br>C26 13.754 2.980 4.752<br>C27 14.206 1.668 4.687<br>H35 14.655 1.183 5.548<br>C28 14.080 0.978 3.485<br>H36 14.437 -0.048 3.431<br>C29 13.508 1.567 2.357

C21 6.516 2.306 5.959	H37 13.419 1.003 1.433
H3 4.509 -0.102 -2.666	C30 13.056 2.873 2.431
H4 7.879 2.491 -2.123	H38 12.608 3.363 1.566
H5 4.145 -1.209 0.615	C31 13.174 3.602 3.624
H6 3.236 -0.858 -0.867	N1 12.166 8.319 3.690
H7 3.158 0.238 0.530	N2 12.415 8.642 5.838
H8 6.427 0.220 -4.490	C1 12.464 7.684 4.870
H9 7.344 1.732 -4.329	C2 11.681 9.682 3.926
H10 5.581 1.766 -4.501	C3 12.278 9.980 5.293
H11 8.154 3.560 0.034	C4 11.831 7.682 2.460
H12 8.741 2.115 0.838	C5 12.800 7.616 1.449
H13 7.327 2.985 1.486	C6 12.421 7.080 0.215
H14 2.362 -1.060 6.600	C7 11.137 6.586 -0.009
H15 5.242 1.659 8.240	C8 10.214 6.622 1.041
H16 2.532 -0.244 3.344	C9 10.537 7.156 2.285
H17 2.464 -1.707 4.352	C10 14.226 8.004 1.701
H18 3.915 -1.339 3.398	C11 10.760 5.986 -1.329
H19 4.081 -0.494 9.550	C12 9.557 7.084 3.420
H20 2.424 -0.724 8.973	C13 12.672 8.411 7.230
H21 2.975 0.875 9.503	C14 13.977 8.478 7.732
H22 6.462 2.920 5.052	C15 14.169 8.150 9.075
H23 7.442 1.714 5.912	C16 13.108 7.810 9.908
H24 6.619 2.974 6.821	C17 11.809 7.864 9.396
H114 7.841 0.468 2.007	C18 11.565 8.175 8.064
C115 6.877 -1.388 1.423	C19 15.133 8.977 6.918
H116 6.200 -1.122 3.939	C20 13.346 7.389 11.326
H117 7.339 0.196 4.280	C21 10.165 8.278 7.541
N118 8.250 -1.887 1.006	H3 13.161 7.026 -0.585
H119 6.444 -2.179 2.049	H4 9.218 6.202 0.888
H120 6.307 -1.326 0.489	H5 14.334 8.896 2.331
C121 9.179 -1.936 2.177	H6 14.768 8.162 0.763
H122 10.127 -2.378 1.859	H7 14.736 7.210 2.267
H123 9.356 -0.925 2.550	H8 11.414 6.333 -2.135
H124 8.726 -2.549 2.961	H9 9.725 6.224 -1.599
C125 8.088 -3.271 0.461	H10 10.836 4.892 -1.295
H126 9.060 -3.635 0.119	H11 8.594 6.693 3.078
H127 7.702 -3.924 1.247	H12 9.366 8.054 3.902
H128 7.386 -3.239 -0.376	H13 9.925 6.419 4.218
C129 8.821 -1.019 -0.077	H14 15.182 8.170 9.478
H130 9.781 -1.439 -0.386	H15 10.964 7.655 10.053
H131 8.122 -0.997 -0.918	H16 16.046 8.418 7.146
H132 8.963 -0.003 0.297	H17 15.324 10.032 7.157
Ru1 3.734 3.028 2.378	H18 14.971 8.878 5.841
Cl1 1.696 1.717 2.113	H19 12.537 7.718 11.987
Cl2 5.735 4.287 3.088	H20 14.290 7.787 11.714
N68 2.513 5.973 2.123	H21 13.397 6.295 11.400
N69 2.043 5.063 4.008	H22 9.956 7.472 6.828
C70 2.626 4.796 2.806	H23 9.990 9.237 7.031
C71 1.879 6.945 2.890	H24 9.441 8.201 8.357
C72 1.587 6.375 4.075	H114 10.578 9.655 4.012
C73 2.748 6.205 0.727	C115 12.108 10.659 2.834
C74 3.881 6.918 0.325	H116 13.268 10.466 5.200
C75 4.015 7.197 -1.035	H117 11.640 10.612 5.924
C76 3.050 6.814 -1.964	N118 10.974 11.342 2.086
C77 1.919 6.136 -1.514	H119 12.714 11.475 3.247
C78 1.742 5.821 -0.172	H120 12.696 10.147 2.066
C79 4.911 7.369 1.313	C121 10.103 12.114 3.026
C80 3.215 7.129 -3.421	H122 9.355 12.659 2.445
C81 0.526 5.081 0.294	H123 9.601 11.429 3.713
C82 1.702 4.124 5.042	H124 10.725 12.817 3.587
C83 2.545 3.979 6.147	C125 11.590 12.297 1.111
C84 2.100 3.175 7.195	H126 10.795 12.794 0.551
C85 0.864 2.534 7.157	H127 12.178 13.038 1.658
C86 0.052 2.714 6.039	H128 12.234 11.737 0.428
C87 0.436 3.523 4.976	C129 10.150 10.352 1.314

C88 3.846 4.716 6.238<br>C89 0.396 1.672 8.289<br>C90 -0.497 3.772 3.832<br>C40 4.158 3.509 0.646<br>C41 3.616 3.280 -0.689<br>C42 2.444 2.564 -0.991<br>C43 2.032 2.407 -2.306<br>C44 2.774 2.957 -3.352<br>C45 3.935 3.670 -3.073<br>C46 4.348 3.831 -1.757<br>H1 1.696 7.935 2.498<br>H2 1.095 6.751 4.962<br>H100 4.900 7.737 -1.375<br>H101 1.160 5.825 -2.234<br>H102 4.492 8.089 2.028<br>H103 5.749 7.856 0.804<br>H104 5.299 6.529 1.904<br>H105 4.272 7.231 -3.692<br>H106 2.719 8.071 -3.683<br>H107 2.775 6.345 -4.047<br>H108 -0.160 4.900 -0.540<br>H109 -0.017 5.642 1.066<br>H110 0.787 4.108 0.735<br>H111 2.733 3.070 8.078<br>H112 -0.928 2.237 6.009<br>H113 4.492 4.529 5.371<br>H115 4.387 4.430 7.146<br>H118 3.685 5.802 6.275<br>H121 -0.695 1.694 8.385<br>H125 0.827 1.987 9.246<br>H129 0.685 0.625 8.128<br>H133 -0.089 3.356 2.902<br>H134 -0.668 4.845 3.673<br>H135 -1.467 3.300 4.016<br>H58 5.065 4.138 0.599<br>H59 1.864 2.138 -0.176<br>H60 1.120 1.854 -2.519<br>H61 2.443 2.833 -4.381<br>H62 4.515 4.114 -3.880<br>H63 5.250 4.403 -1.538	H130 9.396 10.904 0.748<br>H131 10.806 9.793 0.640<br>H132 9.665 9.655 2.001
**1-Hov-TS**	**1-Hov-a**
Ru1 12.959 5.715 5.183<br>Cl1 11.265 5.283 6.747<br>Cl2 15.218 6.266 4.701<br>O1 11.527 2.731 5.062<br>C22 12.497 4.838 3.676<br>H27 11.857 5.169 2.853<br>C23 10.747 1.635 5.578<br>H28 11.411 0.977 6.160<br>C24 9.718 2.236 6.505<br>H29 10.195 2.776 7.327<br>H30 9.081 1.444 6.915<br>H31 9.085 2.945 5.957<br>C25 10.105 0.874 4.434<br>H32 10.844 0.470 3.734<br>H33 9.437 1.544 3.878<br>H34 9.509 0.039 4.818<br>C26 12.664 2.462 4.396<br>C27 13.342 1.239 4.391<br>H35 12.965 0.390 4.953<br>C28 14.515 1.107 3.659<br>H36 15.034 0.151 3.667<br>C29 15.033 2.171 2.922<br>H37 15.953 2.052 2.356	Ru1 13.033 5.901 5.287<br>Cl1 11.292 5.185 6.716<br>Cl2 15.278 6.360 4.658<br>O1 10.973 3.172 2.411<br>C22 12.490 4.938 3.817<br>H27 11.794 5.295 3.057<br>C23 10.079 2.330 1.670<br>H28 9.926 1.398 2.235<br>C24 8.769 3.080 1.599<br>H29 8.419 3.347 2.602<br>H30 8.003 2.470 1.109<br>H31 8.900 4.002 1.018<br>C25 10.655 2.048 0.297<br>H32 11.613 1.521 0.349<br>H33 10.823 2.998 -0.227<br>H34 9.963 1.442 -0.298<br>C26 12.048 2.640 3.025<br>C27 12.377 1.285 3.012<br>H35 11.778 0.570 2.457<br>C28 13.480 0.831 3.726<br>H36 13.716 -0.231 3.704<br>C29 14.283 1.706 4.452<br>H37 15.151 1.340 4.993

C30 14.370 3.384 2.933<br>H38 14.769 4.241 2.391<br>C31 13.182 3.543 3.659<br>N1 12.165 8.198 3.720<br>N2 12.448 8.526 5.868<br>C1 12.447 7.566 4.909<br>C2 11.722 9.577 3.948<br>C3 12.332 9.864 5.308<br>C4 11.746 7.570 2.509<br>C5 12.654 7.476 1.443<br>C6 12.186 6.971 0.228<br>C7 10.870 6.538 0.067<br>C8 10.008 6.604 1.164<br>C9 10.423 7.105 2.397<br>C10 14.106 7.811 1.612<br>C11 10.403 5.969 -1.238<br>C12 9.504 7.047 3.583<br>C13 12.629 8.391 7.287<br>C14 13.905 8.430 7.850<br>C15 14.000 8.324 9.241<br>C16 12.878 8.220 10.051<br>C17 11.615 8.263 9.453<br>C18 11.465 8.358 8.078<br>C19 15.159 8.634 7.055<br>C20 13.005 8.073 11.537<br>C21 10.100 8.423 7.462<br>H3 12.879 6.892 -0.610<br>H4 8.988 6.232 1.062<br>H5 14.282 8.759 2.136<br>H6 14.618 7.849 0.645<br>H7 14.600 7.057 2.241<br>H8 10.958 6.388 -2.084<br>H9 9.336 6.151 -1.401<br>H10 10.552 4.883 -1.261<br>H11 8.518 6.673 3.292<br>H12 9.352 8.019 4.076<br>H13 9.904 6.374 4.357<br>H14 14.991 8.339 9.696<br>H15 10.725 8.219 10.081<br>H16 15.805 7.750 7.102<br>H17 15.725 9.481 7.461<br>H18 14.971 8.809 5.992<br>H19 12.229 8.639 12.064<br>H20 13.981 8.412 11.896<br>H21 12.892 7.023 11.832<br>H22 9.901 7.518 6.874<br>H23 9.981 9.290 6.795<br>H24 9.330 8.498 8.236<br>H114 10.620 9.579 4.036<br>C115 12.165 10.532 2.845<br>H116 13.331 10.327 5.218<br>H117 11.711 10.506 5.946<br>N118 11.041 11.299 2.167<br>H119 12.851 11.297 3.231<br>H120 12.670 9.989 2.040<br>C121 10.260 12.095 3.165<br>H122 9.519 12.697 2.633<br>H123 9.752 11.424 3.859<br>H124 10.947 12.747 3.712<br>C125 11.667 12.242 1.186<br>H126 10.877 12.794 0.671<br>H127 12.318 12.937 1.721<br>H128 12.250 11.664 0.464<br>C129 10.130 10.373 1.415<br>H130 9.379 10.976 0.899	C30 13.972 3.052 4.459<br>H38 14.625 3.756 4.977<br>C31 12.859 3.552 3.755<br>N1 12.065 8.285 3.744<br>N2 12.402 8.643 5.882<br>C1 12.424 7.676 4.924<br>C2 11.582 9.648 3.980<br>C3 12.230 9.970 5.318<br>C4 11.662 7.627 2.545<br>C5 12.577 7.523 1.488<br>C6 12.132 6.950 0.293<br>C7 10.828 6.482 0.142<br>C8 9.958 6.567 1.232<br>C9 10.356 7.114 2.449<br>C10 14.016 7.912 1.647<br>C11 10.366 5.897 -1.159<br>C12 9.445 7.043 3.641<br>C13 12.726 8.475 7.269<br>C14 14.051 8.580 7.703<br>C15 14.300 8.393 9.064<br>C16 13.275 8.153 9.973<br>C17 11.957 8.137 9.509<br>C18 11.657 8.303 8.165<br>C19 15.176 8.977 6.795<br>C20 13.564 7.921 11.425<br>C21 10.236 8.302 7.689<br>H3 12.835 6.849 -0.534<br>H4 8.947 6.171 1.140<br>H5 14.162 8.785 2.295<br>H6 14.488 8.101 0.678<br>H7 14.568 7.105 2.149<br>H8 9.916 6.662 -1.804<br>H9 9.605 5.123 -1.004<br>H10 11.197 5.451 -1.715<br>H11 8.477 6.614 3.363<br>H12 9.249 8.017 4.115<br>H13 9.878 6.404 4.427<br>H14 15.328 8.454 9.421<br>H15 11.143 7.987 10.219<br>H16 16.077 8.393 7.006<br>H17 15.427 10.035 6.953<br>H18 14.948 8.821 5.736<br>H19 12.822 8.413 12.064<br>H20 14.556 8.290 11.705<br>H21 13.531 6.850 11.661<br>H22 10.023 7.391 7.114<br>H23 10.015 9.164 7.043<br>H24 9.544 8.335 8.536<br>H114 10.483 9.618 4.108<br>C115 11.966 10.608 2.859<br>H116 13.211 10.463 5.183<br>H117 11.611 10.602 5.968<br>N118 10.802 11.304 2.173<br>H119 12.610 11.414 3.230<br>H120 12.500 10.078 2.062<br>C121 9.995 12.088 3.160<br>H122 9.224 12.645 2.621<br>H123 9.522 11.411 3.873<br>H124 10.657 12.782 3.686<br>C125 11.376 12.251 1.164<br>H126 10.557 12.754 0.645<br>H127 12.000 12.987 1.677<br>H128 11.977 11.683 0.450<br>C129 9.926 10.322 1.454<br>H130 9.145 10.879 0.930

H131 10.723 9.799 0.696	H131 10.536 9.755 0.744
H132 9.645 9.684 2.108	H132 9.475 9.630 2.168
**1-Gr_I_P(Cy)$_3$**	**1-Ind_P(Cy)$_3$**
Ru1 3.075 3.368 5.745	Ru1 6.489 9.218 11.387
Cl1 1.423 1.656 5.561	Cl1 7.147 7.055 10.763
Cl2 5.045 4.572 6.408	Cl2 6.872 11.556 11.614
N1 1.642 6.004 5.527	N1 4.852 9.577 13.909
N2 1.382 4.947 7.441	N2 6.945 8.958 14.184
C1 1.949 4.850 6.215	C1 5.991 9.175 13.249
C2 0.675 6.813 6.280	C2 5.041 9.600 15.364
C3 0.850 6.274 7.694	C3 6.542 9.337 15.526
C4 1.681 6.154 4.104	C4 3.492 9.587 13.452
C5 2.643 6.994 3.525	C5 2.909 10.761 12.959
C6 2.578 7.221 2.147	C6 1.540 10.746 12.670
C7 1.614 6.613 1.346	C7 0.750 9.619 12.863
C8 0.689 5.758 1.951	C8 1.367 8.455 13.330
C9 0.700 5.511 3.319	C9 2.723 8.416 13.628
C10 3.771 7.598 4.314	C10 3.667 12.033 12.718
C11 1.599 6.806 -0.138	C11 -0.721 9.637 12.584
C12 -0.283 4.547 3.914	C12 3.329 7.131 14.115
C13 1.421 3.945 8.470	C13 8.254 8.429 13.928
C14 2.482 3.904 9.381	C14 9.347 9.286 13.758
C15 2.476 2.882 10.333	C15 10.584 8.705 13.473
C16 1.441 1.957 10.413	C16 10.750 7.327 13.394
C17 0.359 2.085 9.538	C17 9.648 6.505 13.640
C18 0.322 3.072 8.564	C18 8.393 7.031 13.908
C19 3.551 4.953 9.428	C19 9.254 10.771 13.939
C20 1.468 0.845 11.416	C20 12.076 6.726 13.044
C21 -0.858 3.193 7.648	C21 7.218 6.127 14.129
C40 3.459 3.940 4.014	H3 1.086 11.655 12.271
C41 3.233 3.395 2.697	H4 0.774 7.543 13.431
C42 2.367 2.322 2.407	H5 3.167 12.880 13.208
C43 2.154 1.933 1.095	H6 3.691 12.258 11.644
C44 2.814 2.581 0.052	H7 4.714 11.982 13.028
C45 3.696 3.626 0.318	H8 -1.300 9.688 13.515
C46 3.895 4.035 1.627	H9 -1.031 8.724 12.061
H3 3.331 7.862 1.684	H10 -1.004 10.497 11.970
H4 -0.047 5.247 1.330	H11 2.805 6.277 13.671
H5 3.682 8.693 4.391	H12 3.239 7.010 15.206
H6 4.725 7.411 3.807	H13 4.391 7.040 13.861
H7 3.858 7.169 5.317	H14 11.443 9.358 13.318
H8 2.201 7.665 -0.447	H15 9.770 5.422 13.603
H9 0.580 6.942 -0.518	H16 8.229 11.143 13.869
H10 2.007 5.915 -0.634	H17 9.829 11.296 13.169
H11 -0.987 4.195 3.154	H18 9.677 11.060 14.911
H12 -0.876 4.980 4.734	H19 12.276 5.823 13.631
H13 0.223 3.664 4.334	H20 12.897 7.431 13.207
H14 3.304 2.823 11.039	H21 12.097 6.433 11.987
H15 -0.477 1.390 9.618	H22 6.597 6.089 13.223
H16 3.655 5.501 8.487	H23 6.582 6.459 14.962
H17 4.530 4.508 9.635	H24 7.548 5.106 14.347
H18 3.339 5.666 10.237	H114 4.466 8.773 15.815
H19 0.486 0.699 11.879	C115 4.591 10.944 15.947
H20 2.197 1.031 12.210	H116 7.093 10.236 15.848
H21 1.738 -0.103 10.934	H117 6.765 8.535 16.243
H22 -0.605 2.830 6.644	N118 3.471 10.887 16.974
H23 -1.206 4.231 7.552	H119 5.419 11.448 16.459
H24 -1.696 2.595 8.018	H120 4.238 11.604 15.148
H58 4.086 4.842 3.976	C121 3.891 10.073 18.155
H59 1.864 1.815 3.227	H122 3.101 10.110 18.910
H60 1.476 1.110 0.881	H123 4.051 9.037 17.849
H61 2.648 2.261 -0.975	H124 4.816 10.489 18.563
H62 4.225 4.118 -0.495	C125 3.206 12.292 17.422
H63 4.571 4.862 1.845	H126 2.407 12.282 18.168

H114 -0.344 6.575 5.924<br>C115 0.943 8.311 6.170<br>H116 1.576 6.874 8.272<br>H117 -0.088 6.227 8.263<br>N118 -0.136 9.131 5.481<br>H119 1.049 8.765 7.164<br>H120 1.870 8.496 5.618<br>C121 -1.409 9.072 6.267<br>H122 -2.152 9.712 5.784<br>H123 -1.777 8.044 6.294<br>H124 -1.216 9.427 7.283<br>C125 0.341 10.550 5.438<br>H126 -0.431 11.170 4.977<br>H127 0.537 10.895 6.456<br>H128 1.259 10.594 4.845<br>C129 -0.390 8.681 4.072<br>H130 -1.123 9.360 3.629<br>H131 0.549 8.707 3.510<br>H132 -0.779 7.661 4.069	H127 4.117 12.710 17.857<br>H128 2.900 12.884 16.556<br>C129 2.201 10.336 16.396<br>H130 1.429 10.381 17.169<br>H131 1.913 10.934 15.525<br>H132 2.352 9.303 16.077<br>C31 4.790 9.119 10.645<br>C32 4.220 9.997 9.604<br>C33 4.706 11.147 9.007<br>H33 5.677 11.545 9.294<br>C34 3.917 11.787 8.040<br>H34 4.285 12.689 7.558<br>C35 2.685 11.265 7.683<br>H35 2.090 11.759 6.918<br>C36 2.189 10.098 8.287<br>H36 1.234 9.692 7.964<br>C37 2.949 9.476 9.258<br>C38 2.731 8.249 10.062<br>C39 3.824 8.069 10.859<br>H39 3.984 7.239 11.538<br>C40 1.561 7.381 10.055<br>C41 0.281 7.874 9.766<br>H41 0.152 8.927 9.530<br>C42 -0.832 7.046 9.842<br>H42 -1.818 7.447 9.617<br>C43 -0.688 5.712 10.209<br>H43 -1.560 5.063 10.264<br>C44 0.578 5.208 10.498<br>H44 0.698 4.162 10.770<br>C45 1.691 6.031 10.420<br>H45 2.683 5.624 10.609
**1-GrII_Imes/SIMes**	**2**
N1 6.305 0.828 1.524<br>N2 5.711 0.741 3.632<br>C1 5.360 1.259 2.424<br>C2 7.242 -0.119 2.135<br>C3 7.041 0.151 3.621<br>C4 6.549 1.225 0.173<br>C5 5.908 0.607 -0.909<br>C6 6.305 0.976 -2.198<br>C7 7.283 1.938 -2.429<br>C8 7.871 2.564 -1.326<br>C9 7.514 2.232 -0.025<br>C10 4.784 -0.379 -0.768<br>C11 7.661 2.342 -3.821<br>C12 8.075 2.986 1.145<br>C13 4.979 0.896 4.855<br>C14 4.106 -0.139 5.235<br>C15 3.387 0.018 6.413<br>C16 3.537 1.149 7.218<br>C17 4.466 2.116 6.844<br>C18 5.215 2.006 5.673<br>C19 4.007 -1.407 4.441<br>C20 2.716 1.302 8.460<br>C21 6.283 3.015 5.376<br>H3 5.806 0.508 -3.049<br>H4 8.612 3.347 -1.489<br>H5 4.446 -0.518 0.263<br>H6 5.038 -1.354 -1.214<br>H7 3.902 -0.018 -1.312<br>H8 7.415 1.565 -4.551<br>H9 8.731 2.563 -3.900<br>H10 7.125 3.252 -4.120<br>H11 8.778 3.756 0.814	N1 6.708 1.233 1.784<br>N2 5.872 0.989 3.749<br>C1 5.647 1.613 2.588<br>C2 7.507 0.198 2.362<br>C3 7.161 0.277 3.825<br>C4 6.831 1.507 0.381<br>C5 5.807 1.126 -0.505<br>C6 6.006 1.355 -1.866<br>C7 7.168 1.947 -2.358<br>C8 8.149 2.331 -1.446<br>C9 8.002 2.130 -0.077<br>C10 4.502 0.547 -0.034<br>C11 7.343 2.202 -3.823<br>C12 9.073 2.593 0.867<br>C13 5.009 1.059 4.890<br>C14 4.023 0.078 5.027<br>C15 3.195 0.130 6.142<br>C16 3.333 1.130 7.105<br>C17 4.318 2.100 6.923<br>C18 5.171 2.087 5.822<br>C19 3.841 -0.979 3.976<br>C20 2.447 1.153 8.313<br>C21 6.208 3.152 5.626<br>H3 5.220 1.069 -2.565<br>H4 9.054 2.817 -1.812<br>H5 3.997 1.231 0.656<br>H6 4.612 -0.407 0.506<br>H7 3.842 0.348 -0.882<br>H8 6.729 1.527 -4.427<br>H9 8.388 2.082 -4.129<br>H10 7.048 3.227 -4.076<br>H11 9.632 3.426 0.432

H12 8.605 2.342 1.860<br>H13 7.272 3.485 1.706<br>H14 2.697 -0.768 6.720<br>H15 4.619 2.986 7.484<br>H16 3.785 -1.215 3.385<br>H17 3.214 -2.050 4.835<br>H18 4.946 -1.981 4.503<br>H19 3.131 2.063 9.128<br>H20 2.649 0.359 9.014<br>H21 1.691 1.603 8.213<br>H22 6.545 3.054 4.314<br>H23 7.189 2.783 5.953<br>H24 5.959 4.020 5.662<br>H114 8.265 0.137 1.826<br>C115 6.887 -1.555 1.714<br>H116 7.076 -0.754 4.243<br>H117 7.786 0.862 4.013<br>N118 7.996 -2.315 1.009<br>H119 6.609 -2.171 2.579<br>H120 6.038 -1.547 1.024<br>C121 9.174 -2.477 1.916<br>H122 9.931 -3.081 1.410<br>H123 9.592 -1.498 2.157<br>H124 8.848 -2.979 2.832<br>C125 7.463 -3.672 0.660<br>H126 8.246 -4.242 0.154<br>H127 7.161 -4.184 1.577<br>H128 6.601 -3.552 0.000<br>C129 8.416 -1.634 -0.261<br>H130 9.186 -2.246 -0.738<br>H131 7.548 -1.532 -0.916<br>H132 8.813 -0.639 -0.045<br>Ru1 3.726 2.226 2.190<br>Cl1 2.470 0.252 1.789<br>Cl2 4.067 4.259 3.298<br>C40 4.003 2.830 0.476<br>C41 2.956 3.225 -0.430<br>C42 1.588 3.108 -0.115<br>C43 0.626 3.505 -1.028<br>C44 1.008 4.011 -2.270<br>C45 2.356 4.124 -2.602<br>C46 3.323 3.735 -1.690<br>H58 5.017 2.996 0.095<br>H59 1.286 2.688 0.844<br>H60 -0.428 3.415 -0.778<br>H61 0.248 4.318 -2.985<br>H62 2.648 4.519 -3.573<br>H63 4.382 3.820 -1.934	H12 9.813 1.807 1.081<br>H13 8.658 2.931 1.822<br>H14 2.413 -0.621 6.261<br>H15 4.419 2.900 7.656<br>H16 3.678 -0.530 2.987<br>H17 2.979 -1.615 4.197<br>H18 4.714 -1.652 3.914<br>H19 2.884 0.565 9.130<br>H20 1.462 0.727 8.097<br>H21 2.304 2.172 8.688<br>H22 6.148 3.585 4.620<br>H23 7.228 2.764 5.757<br>H24 6.078 3.960 6.350<br>H114 8.568 0.224 2.101<br>H47 7.076 -0.691 4.335<br>H48 7.910 0.877 4.360<br>N65 6.980 -1.151 1.751<br>H66 6.930 -1.024 0.730<br>H67 6.026 -1.318 2.097<br>H68 7.563 -1.965 1.965
**2A**	**2B**
N1 1.140 5.633 5.172<br>N2 1.052 4.655 7.219<br>C1 1.571 4.469 5.877<br>C2 0.952 6.687 6.160<br>C3 0.379 5.894 7.289<br>C4 1.435 6.121 3.848<br>C5 2.550 6.936 3.592<br>C6 2.668 7.526 2.335<br>C7 1.712 7.352 1.340<br>C8 0.595 6.573 1.636<br>C9 0.432 5.970 2.878<br>C10 3.602 7.242 4.618<br>C11 1.894 7.966 -0.013<br>C12 -0.835 5.229 3.182<br>C13 0.538 3.676 8.151	N1 0.973 5.582 5.186<br>N2 0.957 4.730 7.295<br>C1 1.525 4.510 5.995<br>C2 0.071 6.335 5.952<br>C3 -0.304 5.437 7.081<br>C4 1.435 6.274 4.002<br>C5 2.698 6.892 3.935<br>C6 3.023 7.629 2.796<br>C7 2.131 7.796 1.740<br>C8 0.870 7.219 1.861<br>C9 0.496 6.464 2.967<br>C10 3.733 6.745 5.009<br>C11 2.516 8.534 0.497<br>C12 -0.899 5.911 3.013<br>C13 0.822 3.805 8.400

C14 0.829 3.882 9.516<br>C15 0.243 3.045 10.460<br>C16 -0.640 2.023 10.110<br>C17 -0.937 1.865 8.760<br>C18 -0.370 2.669 7.767<br>C19 1.723 4.986 9.998<br>C20 -1.232 1.128 11.152<br>C21 -0.739 2.370 6.344<br>H3 3.540 8.150 2.132<br>H4 -0.182 6.443 0.881<br>H5 3.393 8.188 5.136<br>H6 4.583 7.363 4.146<br>H7 3.685 6.458 5.373<br>H8 2.343 8.962 0.052<br>H9 0.945 8.053 -0.551<br>H10 2.565 7.351 -0.630<br>H11 -1.378 4.980 2.264<br>H12 -1.520 5.850 3.784<br>H13 -0.652 4.294 3.723<br>H14 0.483 3.202 11.513<br>H15 -1.635 1.084 8.457<br>H16 2.442 5.305 9.236<br>H17 2.289 4.663 10.878<br>H18 1.145 5.866 10.314<br>H19 -2.100 0.581 10.774<br>H20 -1.542 1.692 12.038<br>H21 -0.498 0.385 11.491<br>H22 -0.087 1.583 5.940<br>H23 -0.640 3.225 5.668<br>H24 -1.764 1.991 6.279<br>H46 0.289 7.468 5.768<br>H47 1.884 7.169 6.499<br>H48 0.387 6.357 8.280<br>N49 2.897 2.471 6.485<br>N50 2.797 2.920 4.261<br>C51 2.371 3.423 5.529<br>C52 3.565 1.439 5.791<br>C53 2.980 1.482 4.417<br>C54 3.438 2.711 7.804<br>C55 4.349 3.753 8.069<br>C56 4.945 3.815 9.331<br>C57 4.674 2.885 10.330<br>C58 3.785 1.854 10.030<br>C59 3.170 1.738 8.788<br>C60 4.689 4.840 7.094<br>C61 5.292 3.001 11.688<br>C62 2.272 0.561 8.542<br>C63 2.470 3.332 2.919<br>C64 3.442 4.070 2.225<br>C65 3.236 4.365 0.883<br>C66 2.105 3.922 0.200<br>C67 1.178 3.155 0.899<br>C68 1.339 2.840 2.247<br>C69 4.716 4.492 2.892<br>C70 1.873 4.281 -1.234<br>C71 0.309 1.964 2.896<br>H72 5.645 4.626 9.534<br>H73 3.562 1.106 10.792<br>H74 4.588 4.547 6.044<br>H75 5.707 5.207 7.258<br>H76 4.018 5.698 7.235<br>H77 6.203 3.606 11.670<br>H78 5.538 2.020 12.106<br>H79 4.597 3.482 12.389<br>H80 1.730 0.299 9.456	C14 1.243 4.284 9.653<br>C15 1.012 3.506 10.781<br>C16 0.348 2.283 10.708<br>C17 -0.110 1.864 9.462<br>C18 0.101 2.605 8.299<br>C19 1.889 5.629 9.814<br>C20 0.160 1.446 11.934<br>C21 -0.445 2.081 7.004<br>H3 4.013 8.081 2.733<br>H4 0.147 7.349 1.054<br>H5 3.445 7.180 5.979<br>H6 4.665 7.240 4.718<br>H7 3.947 5.690 5.202<br>H8 3.433 9.114 0.631<br>H9 1.724 9.217 0.170<br>H10 2.687 7.828 -0.327<br>H11 -1.215 5.598 2.012<br>H12 -1.623 6.673 3.336<br>H13 -0.995 5.048 3.679<br>H14 1.352 3.870 11.752<br>H15 -0.658 0.924 9.387<br>H16 2.675 5.808 9.072<br>H17 2.342 5.727 10.805<br>H18 1.153 6.444 9.729<br>H19 -0.555 0.634 11.770<br>H20 -0.193 2.045 12.780<br>H21 1.113 0.993 12.241<br>H22 0.333 1.994 6.237<br>H23 -1.234 2.718 6.583<br>H24 -0.889 1.091 7.149<br>H46 -0.747 6.783 5.380<br>H47 -0.625 5.951 7.995<br>H48 -1.127 4.778 6.757<br>N49 3.054 2.564 6.424<br>N50 2.837 3.222 4.259<br>C51 2.382 3.538 5.584<br>C52 3.791 1.679 5.623<br>C53 3.181 1.802 4.269<br>C54 3.470 2.573 7.810<br>C55 4.273 3.597 8.347<br>C56 4.709 3.482 9.667<br>C57 4.397 2.380 10.458<br>C58 3.647 1.359 9.880<br>C59 3.179 1.424 8.571<br>C60 4.630 4.837 7.584<br>C61 4.811 2.307 11.894<br>C62 2.408 0.254 8.034<br>C63 2.268 3.591 2.980<br>C64 3.161 4.151 2.051<br>C65 2.713 4.409 0.760<br>C66 1.416 4.101 0.357<br>C67 0.574 3.489 1.282<br>C68 0.973 3.215 2.589<br>C69 4.595 4.435 2.396<br>C70 0.933 4.451 -1.015<br>C71 0.000 2.545 3.515<br>H72 5.309 4.288 10.090<br>H73 3.401 0.478 10.475<br>H74 5.210 4.653 6.667<br>H75 5.233 5.510 8.200<br>H76 3.729 5.372 7.267<br>H77 5.646 2.980 12.113<br>H78 5.104 1.292 12.180<br>H79 3.977 2.596 12.547<br>H80 1.782 -0.180 8.819

H81 2.846 -0.334 8.261<br>H82 1.533 0.754 7.758<br>H83 3.987 4.951 0.351<br>H84 0.294 2.784 0.379<br>H85 4.547 4.907 3.891<br>H86 5.238 5.252 2.302<br>H87 5.413 3.642 2.985<br>H88 1.429 3.451 -1.793<br>H89 2.800 4.574 -1.736<br>H90 1.177 5.128 -1.312<br>H91 0.244 2.131 3.973<br>H92 0.524 0.899 2.732<br>H93 -0.683 2.145 2.467<br>H94 3.562 0.474 6.307<br>H95 3.632 1.088 3.627<br>H96 2.044 0.900 4.410<br>N97 -1.153 5.673 6.972<br>H98 -1.475 4.846 7.496<br>H99 -1.238 5.484 5.964<br>H100 -1.737 6.476 7.220<br>N101 5.093 1.808 5.657<br>H102 5.422 2.158 6.569<br>H103 5.168 2.565 4.963<br>H104 5.677 1.019 5.368	H81 3.080 -0.550 7.700<br>H82 1.756 0.521 7.196<br>H83 3.401 4.861 0.043<br>H84 -0.436 3.210 0.977<br>H85 4.703 4.937 3.364<br>H86 5.056 5.076 1.639<br>H87 5.193 3.511 2.424<br>H88 0.504 5.464 -1.026<br>H89 0.151 3.767 -1.360<br>H90 1.746 4.439 -1.748<br>H91 -0.122 3.101 4.451<br>H92 0.303 1.525 3.788<br>H93 -0.983 2.460 3.040<br>H94 3.892 0.670 6.035<br>H95 3.849 1.556 3.435<br>H96 2.304 1.136 4.208<br>H97 5.607 2.432 6.473<br>H98 5.272 3.056 4.951<br>H99 5.933 1.523 5.109<br>N100 5.288 2.201 5.523<br>N101 0.835 7.585 6.565<br>H102 1.368 8.032 5.808<br>H103 1.487 7.225 7.274<br>H104 0.213 8.277 6.992
**2-GrI**	**2-Ind**
Ru1 3.180 3.223 5.590<br>Cl1 1.496 1.535 5.298<br>Cl2 4.897 4.908 6.435<br>P1 4.942 1.482 5.542<br>N1 1.761 5.916 5.577<br>N2 1.404 4.818 7.452<br>C1 1.886 4.672 6.200<br>C2 1.577 6.918 6.575<br>C3 0.889 6.168 7.690<br>C4 1.662 6.143 4.156<br>C5 2.597 6.925 3.455<br>C6 2.429 7.083 2.079<br>C7 1.382 6.485 1.384<br>C8 0.463 5.731 2.110<br>C9 0.575 5.552 3.484<br>C10 3.790 7.579 4.090<br>C11 1.268 6.601 -0.103<br>C12 -0.434 4.711 4.199<br>C13 1.211 3.797 8.452<br>C14 2.098 3.737 9.540<br>C15 1.853 2.794 10.532<br>C16 0.776 1.910 10.463<br>C17 -0.097 2.022 9.387<br>C18 0.080 2.974 8.383<br>C19 3.275 4.658 9.666<br>C20 0.573 0.887 11.539<br>C21 -0.936 3.085 7.289<br>C22 6.657 2.066 5.110<br>C23 7.733 0.976 5.188<br>C24 9.124 1.593 5.057<br>C25 9.253 2.396 3.766<br>C26 8.166 3.461 3.673<br>C27 6.775 2.844 3.797<br>C28 5.126 0.828 7.277<br>C29 5.900 1.747 8.226<br>C30 6.068 1.070 9.585<br>C31 4.724 0.675 10.188<br>C32 3.910 -0.187 9.229<br>C33 3.753 0.493 7.873	Ru1 6.418 8.753 11.320<br>Cl1 6.308 6.343 11.353<br>Cl2 6.993 11.207 11.626<br>P1 7.482 8.541 9.082<br>N1 4.985 9.584 13.903<br>N2 7.086 9.045 14.270<br>C1 6.108 8.988 13.338<br>C2 5.383 10.345 15.046<br>C3 6.609 9.616 15.533<br>C4 3.601 9.338 13.573<br>C5 2.780 10.344 13.048<br>C6 1.439 10.037 12.800<br>C7 0.903 8.782 13.063<br>C8 1.750 7.803 13.582<br>C9 3.090 8.054 13.845<br>C10 3.256 11.731 12.722<br>C11 -0.536 8.462 12.804<br>C12 3.960 6.956 14.371<br>C13 8.407 8.468 14.209<br>C14 9.528 9.311 14.112<br>C15 10.790 8.721 14.107<br>C16 10.966 7.344 14.204<br>C17 9.836 6.546 14.363<br>C18 8.551 7.082 14.396<br>C19 9.432 10.805 14.043<br>C20 12.332 6.730 14.146<br>C21 7.388 6.183 14.677<br>C22 7.352 9.991 7.891<br>C23 8.581 10.241 7.004<br>C24 8.391 11.498 6.155<br>C25 7.129 11.421 5.304<br>C26 5.912 11.169 6.184<br>C27 6.095 9.902 7.013<br>C28 9.313 8.378 9.396<br>C29 9.860 9.551 10.215<br>C30 11.385 9.513 10.228<br>C31 11.903 8.178 10.757<br>C32 11.273 6.984 10.047<br>C33 9.747 7.062 10.050

C34 4.482 -0.010 4.516	C34 7.072 6.965 8.171
C35 5.033 -1.372 4.955	C35 7.897 6.702 6.905
C36 4.399 -2.484 4.119	C36 7.666 5.271 6.422
C37 4.601 -2.261 2.623	C37 6.185 4.997 6.182
C38 4.083 -0.890 2.200	C38 5.362 5.289 7.433
C39 4.746 0.211 3.022	C39 5.580 6.721 7.916
C40 3.521 3.851 3.885	H3 0.801 10.810 12.369
C41 3.155 3.456 2.540	H4 1.353 6.806 13.780
C42 2.138 2.542 2.210	H5 3.157 12.410 13.587
C43 1.904 2.204 0.887	H6 2.633 12.171 11.935
C44 2.687 2.749 -0.130	H7 4.284 11.743 12.337
C45 3.694 3.660 0.176	H8 -1.071 8.269 13.742
C46 3.912 4.024 1.496	H9 -0.630 7.560 12.185
H3 3.157 7.680 1.530	H10 -1.049 9.280 12.287
H4 -0.367 5.255 1.587	H11 3.358 6.094 14.673
H5 3.525 8.530 4.584	H12 4.545 7.275 15.244
H6 4.522 7.853 3.324	H13 4.672 6.614 13.605
H7 4.307 6.913 4.794	H14 11.666 9.365 14.018
H8 1.789 7.484 -0.486	H15 9.956 5.470 14.491
H9 0.222 6.651 -0.425	H16 8.815 11.137 13.201
H10 1.712 5.718 -0.584	H17 10.427 11.244 13.924
H11 -1.292 4.503 3.554	H18 9.013 11.233 14.967
H12 -0.810 5.203 5.106	H19 12.502 6.050 14.989
H13 -0.004 3.745 4.505	H20 13.118 7.492 14.156
H14 2.525 2.747 11.391	H21 12.453 6.140 13.228
H15 -0.963 1.361 9.331	H22 6.802 6.000 13.768
H16 3.889 4.671 8.757	H23 6.716 6.608 15.433
H17 3.918 4.350 10.497	H24 7.737 5.216 15.052
H18 2.963 5.693 9.882	H25 7.237 10.863 8.559
H19 -0.301 0.259 11.344	H26 8.740 9.375 6.341
H20 0.437 1.363 12.518	H27 9.492 10.349 7.606
H21 1.449 0.231 11.625	H28 8.324 12.372 6.823
H22 -0.560 2.621 6.369	H29 9.277 11.654 5.526
H23 -1.189 4.127 7.061	H30 7.003 12.340 4.717
H24 -1.860 2.573 7.573	H31 7.229 10.597 4.578
H25 6.849 2.801 5.912	H32 4.996 11.087 5.583
H26 7.578 0.253 4.370	H33 5.764 12.028 6.860
H27 7.659 0.408 6.126	H34 5.199 9.710 7.618
H28 9.303 2.256 5.920	H35 6.200 9.051 6.324
H29 9.889 0.808 5.103	H36 9.782 8.413 8.398
H30 10.248 2.852 3.695	H37 9.475 9.470 11.248
H31 9.161 1.711 2.907	H38 9.501 10.519 9.837
H32 8.246 4.014 2.727	H39 11.755 9.680 9.202
H33 8.300 4.199 4.481	H40 11.777 10.340 10.837
H34 6.014 3.632 3.759	H41 11.651 8.116 11.825
H35 6.593 2.180 2.938	H42 12.998 8.135 10.687
H36 5.700 -0.111 7.190	H43 11.602 6.047 10.517
H37 5.358 2.702 8.338	H44 11.622 6.944 9.002
H38 6.888 2.000 7.821	H45 9.333 6.190 9.527
H39 6.692 0.170 9.458	H46 9.363 7.012 11.084
H40 6.617 1.732 10.267	H47 7.362 6.216 8.925
H41 4.152 1.592 10.404	H48 7.599 7.404 6.109
H42 4.868 0.160 11.147	H49 8.969 6.865 7.084
H43 2.919 -0.407 9.650	H50 8.247 5.086 5.509
H44 4.412 -1.159 9.088	H51 8.049 4.572 7.184
H45 3.158 -0.129 7.192	H52 5.829 5.639 5.359
H46 3.177 1.429 8.001	H53 6.036 3.960 5.854
H47 3.387 -0.038 4.647	H54 5.651 4.594 8.239
H48 6.128 -1.396 4.834	H55 4.294 5.120 7.240
H49 4.828 -1.560 6.018	H56 5.010 6.898 8.836
H50 4.808 -3.456 4.423	H57 5.194 7.417 7.157
H51 3.319 -2.518 4.338	H114 4.581 10.501 15.772
H52 5.676 -2.325 2.389	H102 7.369 10.256 15.995
H53 4.110 -3.056 2.049	H103 6.339 8.828 16.252
H54 2.992 -0.839 2.351	C104 4.702 9.030 10.638

H55 4.261 -0.717 1.130	C105 4.111 10.191 9.901
H56 4.384 1.195 2.697	C106 4.591 11.445 9.555
H57 5.832 0.190 2.833	H107 5.579 11.765 9.865
H58 4.227 4.699 3.882	C108 3.789 12.304 8.792
H59 1.554 2.093 3.010	H109 4.165 13.292 8.532
H60 1.116 1.494 0.645	C110 2.546 11.897 8.338
H61 2.510 2.461 -1.164	H111 1.947 12.559 7.717
H62 4.306 4.087 -0.616	C112 2.059 10.624 8.661
H63 4.695 4.744 1.740	H113 1.105 10.293 8.261
H114 1.077 7.820 6.212	C114 2.825 9.796 9.462
H115 1.137 6.516 8.700	C115 2.573 8.422 9.946
H116 -0.204 6.206 7.574	C116 3.668 8.008 10.642
N117 2.988 7.342 7.076	H117 3.785 7.045 11.124
H118 3.407 8.009 6.422	C118 1.388 7.619 9.673
H119 3.633 6.495 7.052	C119 0.119 8.193 9.509
H120 2.978 7.753 8.012	H120 -0.006 9.267 9.631
	C121 -0.990 7.398 9.250
	H122 -1.968 7.859 9.131
	C123 -0.852 6.017 9.155
	H124 -1.720 5.395 8.947
	C125 0.400 5.432 9.328
	H126 0.513 4.353 9.250
	C127 1.509 6.224 9.582
	H128 2.495 5.769 9.684
	N129 5.820 11.740 14.524
	H130 4.994 12.281 14.252
	H131 6.373 11.611 13.626
	H132 6.366 12.276 15.203
**2-GrII**	**2-GrIIdim**
N1 5.926 0.970 1.635	N1 5.973 0.983 1.639
N2 5.387 0.839 3.742	N2 5.437 0.855 3.749
C1 5.016 1.433 2.583	C1 5.057 1.439 2.587
C2 6.822 0.010 2.167	C2 6.885 0.041 2.176
C3 6.701 0.202 3.648	C3 6.757 0.231 3.657
C4 5.941 1.115 0.207	C4 5.969 1.115 0.209
C5 5.024 0.405 -0.593	C5 5.030 0.407 -0.569
C6 5.205 0.452 -1.974	C6 5.187 0.439 -1.952
C7 6.243 1.173 -2.563	C7 6.223 1.142 -2.567
C8 7.116 1.878 -1.736	C8 7.117 1.846 -1.764
C9 6.989 1.866 -0.350	C9 7.012 1.850 -0.374
C10 3.830 -0.322 -0.031	C10 3.840 -0.302 0.022
C11 6.392 1.201 -4.053	C11 6.331 1.162 -4.061
C12 7.922 2.661 0.515	C12 7.974 2.641 0.462
C13 4.709 0.746 5.003	C13 4.765 0.771 5.015
C14 3.664 -0.185 5.116	C14 3.731 -0.170 5.145
C15 3.156 -0.451 6.385	C15 3.232 -0.426 6.420
C16 3.688 0.145 7.526	C16 3.759 0.194 7.551
C17 4.729 1.058 7.373	C17 4.786 1.120 7.381
C18 5.264 1.374 6.127	C18 5.314 1.423 6.128
C19 3.158 -0.949 3.930	C19 3.227 -0.951 3.970
C20 3.193 -0.211 8.896	C20 3.274 -0.147 8.927
C21 6.434 2.307 6.025	C21 6.472 2.369 6.014
H3 4.485 -0.060 -2.613	H3 4.454 -0.075 -2.575
H4 7.913 2.472 -2.185	H4 7.915 2.423 -2.231
H5 4.036 -1.365 0.261	H5 4.068 -1.293 0.447
H6 3.049 -0.387 -0.795	H6 3.094 -0.485 -0.758
H7 3.385 0.197 0.828	H7 3.346 0.289 0.805
H8 6.825 0.265 -4.428	H8 6.464 0.151 -4.465
H9 7.042 2.020 -4.377	H9 7.171 1.775 -4.402
H10 5.418 1.325 -4.541	H10 5.412 1.565 -4.505
H11 8.516 3.348 -0.095	H11 8.516 3.361 -0.158
H12 8.638 2.026 1.060	H12 8.734 2.006 0.940
H13 7.377 3.247 1.268	H13 7.460 3.193 1.262
H14 2.346 -1.175 6.485	H14 2.432 -1.159 6.534

H15 5.154 1.534 8.259<br>H16 2.766 -0.290 3.145<br>H17 2.357 -1.635 4.221<br>H18 3.959 -1.581 3.499<br>H19 3.964 -0.745 9.464<br>H20 2.305 -0.851 8.856<br>H21 2.937 0.685 9.475<br>H22 6.410 2.906 5.106<br>H23 7.386 1.758 6.060<br>H24 6.450 2.998 6.874<br>H114 7.832 0.054 1.748<br>H47 6.748 -0.718 4.245<br>H48 7.490 0.887 3.992<br>Ru49 3.716 3.043 2.383<br>Cl50 1.699 1.702 2.154<br>Cl51 5.712 4.319 3.090<br>N52 2.564 5.984 2.110<br>N53 2.115 5.059 4.028<br>C54 2.634 4.831 2.807<br>C55 1.852 7.045 2.846<br>C56 1.796 6.479 4.254<br>C57 2.722 6.177 0.703<br>C58 3.829 6.895 0.230<br>C59 3.904 7.154 -1.138<br>C60 2.907 6.745 -2.020<br>C61 1.807 6.058 -1.511<br>C62 1.690 5.764 -0.157<br>C63 4.909 7.360 1.158<br>C64 3.005 7.038 -3.487<br>C65 0.506 4.997 0.350<br>C66 1.725 4.100 5.016<br>C67 2.524 3.890 6.145<br>C68 2.037 3.046 7.142<br>C69 0.794 2.427 7.040<br>C70 0.011 2.688 5.918<br>C71 0.441 3.539 4.907<br>C72 3.831 4.603 6.323<br>C73 0.291 1.502 8.106<br>C74 -0.468 3.869 3.765<br>C75 4.137 3.504 0.639<br>C76 3.594 3.260 -0.689<br>C77 2.427 2.531 -0.981<br>C78 2.014 2.359 -2.293<br>C79 2.752 2.901 -3.346<br>C80 3.909 3.625 -3.079<br>C81 4.320 3.807 -1.766<br>H84 4.770 7.696 -1.523<br>H85 1.025 5.720 -2.193<br>H86 4.536 8.108 1.871<br>H87 5.727 7.822 0.598<br>H88 5.314 6.533 1.755<br>H89 4.046 7.174 -3.802<br>H90 2.463 7.955 -3.747<br>H91 2.570 6.226 -4.082<br>H92 -0.171 4.746 -0.473<br>H93 -0.072 5.566 1.091<br>H94 0.806 4.057 0.834<br>H95 2.643 2.887 8.036<br>H96 -0.980 2.238 5.841<br>H97 4.423 4.634 5.399<br>H98 4.429 4.118 7.102<br>H99 3.674 5.642 6.644<br>H100 -0.802 1.526 8.177<br>H101 0.701 1.751 9.091<br>H102 0.576 0.463 7.886	H15 5.206 1.617 8.257<br>H16 2.812 -0.303 3.187<br>H17 2.446 -1.654 4.273<br>H18 4.037 -1.564 3.529<br>H19 4.050 -0.674 9.496<br>H20 2.388 -0.790 8.901<br>H21 3.020 0.754 9.499<br>H22 6.462 2.931 5.072<br>H23 7.430 1.834 6.093<br>H24 6.458 3.094 6.834<br>H114 7.895 0.102 1.759<br>H47 6.812 -0.689 4.253<br>H48 7.539 0.922 4.006<br>Ru49 3.756 3.031 2.384<br>Cl50 1.723 1.707 2.218<br>Cl51 5.774 4.301 3.046<br>N52 2.538 5.993 2.119<br>N53 2.032 5.061 3.984<br>C54 2.635 4.809 2.789<br>C55 1.901 6.959 2.891<br>C56 1.589 6.374 4.064<br>C57 2.759 6.230 0.720<br>C58 3.897 6.925 0.302<br>C59 4.019 7.195 -1.061<br>C60 3.038 6.819 -1.978<br>C61 1.901 6.161 -1.511<br>C62 1.736 5.858 -0.164<br>C63 4.939 7.375 1.278<br>C64 3.188 7.125 -3.437<br>C65 0.509 5.145 0.318<br>C66 1.655 4.100 4.987<br>C67 2.490 3.888 6.087<br>C68 2.030 3.036 7.090<br>C69 0.788 2.410 7.011<br>C70 -0.020 2.668 5.907<br>C71 0.379 3.526 4.889<br>C72 3.797 4.609 6.223<br>C73 0.319 1.479 8.088<br>C74 -0.545 3.834 3.751<br>C75 4.155 3.487 0.635<br>C76 3.566 3.257 -0.676<br>C77 2.371 2.559 -0.928<br>C78 1.914 2.393 -2.226<br>C79 2.636 2.910 -3.303<br>C80 3.819 3.605 -3.074<br>C81 4.274 3.781 -1.775<br>H82 1.729 7.956 2.510<br>H83 1.085 6.742 4.947<br>H84 4.906 7.722 -1.414<br>H85 1.128 5.861 -2.219<br>H86 4.529 8.098 1.995<br>H87 5.773 7.858 0.759<br>H88 5.331 6.536 1.866<br>H89 4.242 7.195 -3.728<br>H90 2.716 8.082 -3.693<br>H91 2.712 6.354 -4.054<br>H92 -0.185 4.965 -0.510<br>H93 -0.022 5.724 1.085<br>H94 0.757 4.172 0.768<br>H95 2.657 2.877 7.968<br>H96 -1.006 2.205 5.847<br>H97 4.449 4.467 5.350<br>H98 4.331 4.269 7.116<br>H99 3.642 5.691 6.320<br>H100 -0.774 1.461 8.156

H103 -0.114 3.402 2.838<br>H104 -0.531 4.951 3.588<br>H105 -1.481 3.504 3.960<br>H106 5.042 4.134 0.586<br>H107 1.852 2.108 -0.160<br>H108 1.103 1.800 -2.500<br>H109 2.420 2.762 -4.374<br>H110 4.489 4.058 -3.893<br>H111 5.219 4.387 -1.554<br>N112 6.289 -1.404 1.744<br>H113 6.153 -1.383 0.723<br>H115 5.367 -1.550 2.180<br>H116 6.914 -2.175 1.993<br>H117 2.551 6.924 4.918<br>H118 0.815 6.578 4.732<br>H119 0.856 7.194 2.403<br>H120 2.397 7.992 2.776	H101 0.716 1.757 9.070<br>H102 0.646 0.451 7.883<br>H103 -0.140 3.441 2.810<br>H104 -0.697 4.914 3.627<br>H105 -1.523 3.373 3.915<br>H106 5.073 4.096 0.559<br>H107 1.812 2.154 -0.087<br>H108 0.984 1.857 -2.403<br>H109 2.269 2.775 -4.319<br>H110 4.383 4.022 -3.907<br>H111 5.193 4.339 -1.592<br>N112 6.379 -1.386 1.756<br>H113 6.235 -1.369 0.736<br>H115 5.465 -1.552 2.199<br>H116 7.023 -2.143 2.001
**2-Hov**	**2-Hov-TS**
Ru1 13.081 5.835 5.304<br>Cl1 11.346 4.982 6.656<br>Cl2 15.238 6.796 4.585<br>O1 14.218 3.869 5.643<br>C22 12.669 4.914 3.762<br>H27 11.982 5.280 2.991<br>C23 14.816 3.383 6.882<br>H28 15.771 2.915 6.603<br>C24 15.079 4.613 7.719<br>H29 15.708 5.331 7.180<br>H30 15.592 4.325 8.642<br>H31 14.131 5.097 7.998<br>C25 13.883 2.400 7.552<br>H32 13.672 1.529 6.924<br>H33 12.933 2.895 7.785<br>H34 14.334 2.042 8.484<br>C26 14.068 3.068 4.562<br>C27 14.659 1.824 4.392<br>H35 15.293 1.390 5.158<br>C28 14.426 1.132 3.206<br>H36 14.891 0.158 3.071<br>C29 13.617 1.654 2.197<br>H37 13.451 1.089 1.284<br>C30 13.028 2.893 2.373<br>H38 12.392 3.330 1.604<br>C31 13.244 3.622 3.555<br>N1 12.252 8.199 3.647<br>N2 12.390 8.587 5.818<br>C1 12.375 7.585 4.899<br>C2 12.607 9.570 3.775<br>C3 12.233 9.901 5.201<br>C4 11.686 7.617 2.456<br>C5 12.465 7.364 1.316<br>C6 11.846 6.784 0.210<br>C7 10.496 6.443 0.207<br>C8 9.755 6.694 1.360<br>C9 10.321 7.281 2.488<br>C10 13.937 7.646 1.237<br>C11 9.852 5.839 -1.002<br>C12 9.487 7.486 3.714<br>C13 12.410 8.418 7.249<br>C14 13.616 8.576 7.951<br>C15 13.604 8.357 9.327<br>C16 12.439 8.019 10.010<br>C17 11.247 7.954 9.291<br>C18 11.203 8.159 7.916	Ru1 13.262 5.832 5.043<br>Cl1 12.088 5.016 6.867<br>Cl2 15.211 7.043 4.202<br>O1 12.581 2.591 5.006<br>C22 12.707 4.893 3.603<br>H27 11.826 5.084 2.980<br>C23 12.244 1.333 5.633<br>H28 13.171 0.883 6.022<br>C24 11.330 1.656 6.790<br>H29 11.828 2.296 7.523<br>H30 11.016 0.728 7.280<br>H31 10.436 2.180 6.430<br>C25 11.582 0.417 4.623<br>H32 12.218 0.223 3.752<br>H33 10.648 0.870 4.271<br>H34 11.341 -0.546 5.085<br>C26 13.584 2.625 4.117<br>C27 14.525 1.613 3.896<br>H35 14.490 0.686 4.461<br>C28 15.516 1.792 2.941<br>H36 16.240 0.996 2.781<br>C29 15.600 2.963 2.189<br>H37 16.382 3.086 1.447<br>C30 14.681 3.973 2.412<br>H38 14.735 4.906 1.853<br>C31 13.662 3.814 3.364<br>N1 12.050 8.142 3.652<br>N2 12.351 8.503 5.814<br>C1 12.368 7.530 4.871<br>C2 12.243 9.545 3.783<br>C3 11.977 9.799 5.248<br>C4 11.443 7.518 2.502<br>C5 12.122 7.400 1.280<br>C6 11.461 6.788 0.215<br>C7 10.169 6.284 0.331<br>C8 9.525 6.413 1.561<br>C9 10.134 7.026 2.651<br>C10 13.526 7.881 1.053<br>C11 9.496 5.600 -0.818<br>C12 9.406 7.107 3.958<br>C13 12.506 8.362 7.242<br>C14 13.750 8.622 7.831<br>C15 13.869 8.443 9.207<br>C16 12.792 8.042 9.994<br>C17 11.553 7.872 9.381<br>C18 11.381 8.027 8.010

C19 14.889 9.010 7.289<br>C20 12.457 7.747 11.482<br>C21 9.889 8.088 7.202<br>H3 12.447 6.577 -0.676<br>H4 8.699 6.422 1.386<br>H5 14.464 7.370 2.160<br>H6 14.141 8.700 0.980<br>H7 14.392 7.067 0.429<br>H8 10.578 5.305 -1.624<br>H9 9.395 6.615 -1.630<br>H10 9.056 5.140 -0.725<br>H11 8.427 7.331 3.494<br>H12 9.598 8.498 4.124<br>H13 9.769 6.779 4.507<br>H14 14.536 8.468 9.882<br>H15 10.318 7.737 9.818<br>H16 15.671 9.174 8.035<br>H17 14.764 9.963 6.752<br>H18 15.262 8.269 6.572<br>H19 11.573 8.161 11.978<br>H20 13.348 8.165 11.961<br>H21 12.456 6.667 11.674<br>H22 9.771 7.109 6.721<br>H23 9.788 8.859 6.429<br>H24 9.063 8.215 7.907<br>H114 12.185 10.209 2.995<br>H73 12.877 10.648 5.683<br>H74 11.194 10.260 5.258<br>N75 14.160 9.657 3.644<br>H76 14.433 9.542 2.663<br>H77 14.593 8.830 4.142<br>H78 14.551 10.531 4.001	C19 14.935 9.100 7.047<br>C20 12.956 7.800 11.463<br>C21 10.033 7.790 7.404<br>H3 11.983 6.693 -0.737<br>H4 8.512 6.026 1.675<br>H5 13.556 8.956 0.803<br>H6 13.963 7.381 0.184<br>H7 14.183 7.669 1.905<br>H8 9.946 5.878 -1.775<br>H9 8.428 5.840 -0.857<br>H10 9.576 4.510 -0.722<br>H11 8.360 6.812 3.839<br>H12 9.420 8.122 4.376<br>H13 9.855 6.440 4.707<br>H14 14.834 8.628 9.678<br>H15 10.691 7.594 9.988<br>H16 15.353 8.318 6.404<br>H17 15.728 9.437 7.720<br>H18 14.683 9.956 6.401<br>H19 12.061 8.094 12.021<br>H20 13.811 8.346 11.872<br>H21 13.124 6.734 11.659<br>H22 10.003 6.802 6.926<br>H23 9.766 8.537 6.647<br>H24 9.257 7.809 8.174<br>H114 11.666 10.138 3.067<br>H73 12.572 10.605 5.693<br>H74 10.914 10.024 5.419<br>N75 13.752 9.831 3.502<br>H76 13.935 9.771 2.495<br>H77 14.331 9.058 3.928<br>H78 14.066 10.742 3.846
**2-Hov-a**	**2-GrI_P(Cy)$_3$**
Ru1 13.059 5.879 5.432<br>Cl1 11.613 4.992 7.026<br>Cl2 15.180 6.722 4.547<br>O1 11.121 3.108 2.437<br>C22 12.458 4.879 3.996<br>H27 11.543 5.151 3.461<br>C23 10.309 2.288 1.578<br>H28 10.394 1.242 1.911<br>C24 8.894 2.771 1.797<br>H29 8.580 2.609 2.832<br>H30 8.200 2.245 1.134<br>H31 8.837 3.848 1.588<br>C25 10.772 2.441 0.144<br>H32 11.798 2.086 -0.003<br>H33 10.739 3.502 -0.135<br>H34 10.117 1.884 -0.535<br>C26 12.344 2.718 2.813<br>C27 12.927 1.495 2.473<br>H35 12.405 0.789 1.836<br>C28 14.184 1.169 2.963<br>H36 14.619 0.211 2.689<br>C29 14.896 2.042 3.788<br>H37 15.882 1.773 4.153<br>C30 14.334 3.257 4.118<br>H38 14.898 3.964 4.728<br>C31 13.056 3.631 3.645<br>N1 12.134 8.089 3.676<br>N2 12.454 8.646 5.799<br>C1 12.366 7.580 4.961<br>C2 12.498 9.462 3.671<br>C3 12.250 9.907 5.095	Ru1 3.466 3.668 5.936<br>Cl1 2.897 1.420 5.958<br>Cl2 4.660 5.670 6.703<br>N1 1.533 5.902 5.308<br>N2 1.258 4.849 7.238<br>C1 1.908 4.782 6.050<br>C2 0.968 6.857 6.200<br>C3 0.390 6.019 7.318<br>C4 1.513 6.088 3.874<br>C5 2.455 6.887 3.213<br>C6 2.332 7.039 1.830<br>C7 1.311 6.436 1.105<br>C8 0.376 5.667 1.799<br>C9 0.453 5.484 3.172<br>C10 3.589 7.592 3.897<br>C11 1.214 6.575 -0.382<br>C12 -0.577 4.653 3.872<br>C13 1.274 3.828 8.256<br>C14 2.033 4.021 9.422<br>C15 2.052 2.994 10.361<br>C16 1.331 1.815 10.181<br>C17 0.534 1.692 9.045<br>C18 0.485 2.683 8.071<br>C19 2.780 5.292 9.700<br>C20 1.407 0.704 11.182<br>C21 -0.376 2.489 6.862<br>C40 3.864 3.851 4.137<br>C41 3.197 3.432 2.937<br>C42 2.051 2.611 2.931<br>C43 1.503 2.183 1.734<br>C44 2.076 2.569 0.523

C4 11.475 7.447 2.565<br>C5 12.181 7.035 1.427<br>C6 11.459 6.461 0.378<br>C7 10.080 6.292 0.429<br>C8 9.413 6.683 1.591<br>C9 10.084 7.260 2.664<br>C10 13.668 7.163 1.275<br>C11 9.318 5.726 -0.730<br>C12 9.332 7.636 3.903<br>C13 12.592 8.580 7.233<br>C14 13.841 8.830 7.823<br>C15 13.939 8.716 9.207<br>C16 12.843 8.386 10.001<br>C17 11.605 8.218 9.385<br>C18 11.450 8.317 8.006<br>C19 15.044 9.249 7.030<br>C20 12.989 8.218 11.482<br>C21 10.096 8.132 7.397<br>H3 12.002 6.136 -0.510<br>H4 8.334 6.542 1.660<br>H5 14.207 7.004 2.216<br>H6 13.953 8.135 0.833<br>H7 14.042 6.415 0.569<br>H8 8.671 6.489 -1.180<br>H9 8.664 4.901 -0.419<br>H10 9.985 5.353 -1.514<br>H11 8.256 7.510 3.762<br>H12 9.509 8.681 4.191<br>H13 9.630 7.010 4.755<br>H14 14.904 8.898 9.680<br>H15 10.729 8.000 9.996<br>H16 15.884 9.463 7.697<br>H17 14.858 10.170 6.457<br>H18 15.369 8.472 6.329<br>H19 12.103 8.577 12.017<br>H20 13.863 8.752 11.866<br>H21 13.113 7.159 11.739<br>H22 9.995 7.115 6.996<br>H23 9.900 8.841 6.583<br>H24 9.315 8.268 8.151<br>H114 12.010 10.044 2.884<br>H73 12.939 10.680 5.457<br>H74 11.223 10.284 5.213<br>N75 14.035 9.521 3.392<br>H76 14.217 9.276 2.413<br>H77 14.513 8.767 3.951<br>H78 14.457 10.432 3.593	C45 3.207 3.384 0.507<br>C46 3.767 3.806 1.701<br>H3 3.068 7.648 1.306<br>H4 -0.433 5.187 1.247<br>H5 3.306 8.615 4.200<br>H6 4.423 7.730 3.203<br>H7 3.983 7.044 4.759<br>H8 1.920 7.315 -0.772<br>H9 0.205 6.870 -0.690<br>H10 1.428 5.615 -0.870<br>H11 -1.309 4.261 3.161<br>H12 -1.129 5.231 4.626<br>H13 -0.126 3.795 4.387<br>H14 2.644 3.123 11.267<br>H15 -0.064 0.791 8.907<br>H16 3.555 5.491 8.952<br>H17 3.270 5.241 10.676<br>H18 2.106 6.162 9.735<br>H19 0.443 0.195 11.292<br>H20 1.720 1.065 12.166<br>H21 2.136 -0.051 10.862<br>H22 0.227 2.117 6.022<br>H23 -0.872 3.414 6.542<br>H24 -1.154 1.746 7.058<br>H58 4.872 4.277 3.979<br>H59 1.633 2.282 3.880<br>H60 0.630 1.534 1.741<br>H61 1.644 2.225 -0.414<br>H62 3.659 3.674 -0.438<br>H63 4.659 4.431 1.699<br>H114 0.283 7.557 5.713<br>H64 0.442 6.485 8.311<br>H65 -0.661 5.763 7.116<br>N65 2.155 7.699 6.759<br>H66 2.518 8.313 6.022<br>H67 2.956 7.051 6.989<br>H68 1.910 8.266 7.575
**2-Ind_P(Cy)$_3$**	**2-GrII_Imes/SIMes**
Ru1 6.410 9.232 11.483<br>Cl1 6.999 7.104 10.760<br>Cl2 6.682 11.635 11.928<br>N1 4.821 9.610 14.012<br>N2 6.954 9.056 14.253<br>C1 5.955 9.150 13.338<br>C2 5.237 10.177 15.249<br>C3 6.505 9.427 15.591<br>C4 3.424 9.437 13.685<br>C5 2.661 10.466 13.122<br>C6 1.315 10.209 12.852<br>C7 0.717 8.988 13.139<br>C8 1.504 7.988 13.713<br>C9 2.849 8.189 13.994<br>C10 3.202 11.824 12.783<br>C11 -0.728 8.731 12.847	N1 6.286 0.643 1.476<br>N2 5.677 0.644 3.608<br>C1 5.372 1.171 2.396<br>C2 6.873 -0.517 2.050<br>C3 6.844 -0.233 3.536<br>C4 6.699 1.244 0.232<br>C5 6.339 0.698 -1.009<br>C6 6.723 1.380 -2.164<br>C7 7.435 2.575 -2.119<br>C8 7.790 3.081 -0.869<br>C9 7.440 2.437 0.312<br>C10 5.544 -0.565 -1.163<br>C11 7.788 3.317 -3.372<br>C12 7.805 3.048 1.629<br>C13 5.048 0.977 4.862<br>C14 4.100 0.100 5.415

C12 3.666 7.075 14.572<br>C13 8.252 8.485 13.999<br>C14 9.375 9.320 13.887<br>C15 10.597 8.723 13.587<br>C16 10.727 7.346 13.422<br>C17 9.603 6.546 13.618<br>C18 8.358 7.088 13.914<br>C19 9.310 10.801 14.111<br>C20 12.040 6.735 13.043<br>C21 7.181 6.186 14.118<br>H3 0.718 10.997 12.390<br>H4 1.057 7.018 13.931<br>H5 3.062 12.536 13.614<br>H6 2.644 12.254 11.944<br>H7 4.254 11.806 12.481<br>H8 -1.307 8.644 13.775<br>H9 -0.854 7.791 12.295<br>H10 -1.170 9.535 12.250<br>H11 3.042 6.204 14.787<br>H12 4.159 7.364 15.510<br>H13 4.458 6.753 13.882<br>H14 11.478 9.358 13.485<br>H15 9.697 5.463 13.531<br>H16 8.685 11.305 13.364<br>H17 10.311 11.240 14.055<br>H18 8.915 11.050 15.107<br>H19 12.191 5.769 13.536<br>H20 12.881 7.388 13.298<br>H21 12.080 6.555 11.962<br>H22 6.546 6.178 13.222<br>H23 6.566 6.488 14.974<br>H24 7.511 5.158 14.293<br>H114 4.453 10.182 16.011<br>H50 7.261 10.026 16.113<br>H51 6.283 8.544 16.208<br>C52 4.708 9.190 10.726<br>C53 4.265 10.096 9.646<br>C54 4.867 11.191 9.056<br>H55 5.830 11.557 9.405<br>C56 4.207 11.827 7.992<br>H57 4.666 12.689 7.515<br>C58 2.989 11.355 7.537<br>H59 2.498 11.845 6.700<br>C60 2.375 10.241 8.137<br>H61 1.436 9.863 7.741<br>C62 3.004 9.635 9.204<br>C63 2.653 8.441 10.011<br>C64 3.679 8.198 10.885<br>H65 3.727 7.376 11.589<br>C66 1.432 7.663 9.882<br>C67 0.220 8.271 9.523<br>H68 0.185 9.347 9.368<br>C69 -0.945 7.523 9.424<br>H70 -1.878 8.010 9.152<br>C71 -0.919 6.155 9.680<br>H72 -1.829 5.567 9.592<br>C73 0.276 5.540 10.050<br>H74 0.300 4.471 10.245<br>C75 1.440 6.286 10.152<br>H76 2.380 5.800 10.409<br>N77 5.591 11.668 14.956<br>H78 4.734 12.190 14.746<br>H79 6.162 11.714 14.068<br>H80 6.087 12.133 15.721	C15 3.490 0.472 6.610<br>C16 3.811 1.659 7.265<br>C17 4.807 2.466 6.721<br>C18 5.444 2.147 5.526<br>C19 3.758 -1.222 4.793<br>C20 3.107 2.057 8.525<br>C21 6.500 3.060 4.985<br>H3 6.440 0.966 -3.132<br>H4 8.354 4.012 -0.812<br>H5 4.705 -0.617 -0.459<br>H6 6.182 -1.462 -1.070<br>H7 5.116 -0.627 -2.168<br>H8 7.749 2.668 -4.252<br>H9 8.790 3.753 -3.310<br>H10 7.089 4.146 -3.543<br>H11 8.512 3.871 1.493<br>H12 8.268 2.321 2.308<br>H13 6.922 3.455 2.140<br>H14 2.746 -0.194 7.048<br>H15 5.097 3.379 7.240<br>H16 3.296 -1.110 3.806<br>H17 3.055 -1.770 5.426<br>H18 4.646 -1.864 4.685<br>H19 3.774 2.598 9.205<br>H20 2.703 1.190 9.056<br>H21 2.266 2.724 8.299<br>H22 6.071 3.722 4.221<br>H23 7.338 2.518 4.533<br>H24 6.904 3.694 5.780<br>H114 7.843 -0.779 1.619<br>H47 6.713 -1.118 4.170<br>H48 7.763 0.283 3.853<br>Ru49 3.668 1.937 2.043<br>Cl50 2.989 -0.329 1.444<br>Cl51 3.581 3.927 3.241<br>C52 3.950 2.656 0.370<br>C53 2.856 3.047 -0.474<br>C54 1.513 2.808 -0.116<br>C55 0.486 3.228 -0.941<br>C56 0.780 3.882 -2.138<br>C57 2.101 4.116 -2.513<br>C58 3.134 3.703 -1.688<br>H59 4.954 2.894 0.002<br>H60 1.276 2.284 0.812<br>H61 -0.547 3.047 -0.659<br>H62 -0.030 4.210 -2.785<br>H63 2.320 4.626 -3.448<br>H64 4.173 3.883 -1.964<br>N65 5.906 -1.711 1.759<br>H66 5.971 -1.974 0.770<br>H67 4.912 -1.385 1.885<br>H68 6.085 -2.535 2.339

3A	3B
N1 1.046 5.491 4.940<br>N2 0.940 4.769 7.113<br>C1 1.555 4.487 5.819<br>C2 0.402 6.492 5.727<br>N5 -0.187 5.706 6.835<br>C4 1.648 6.137 3.790<br>C5 2.861 6.832 3.878<br>C6 3.294 7.550 2.763<br>C7 2.546 7.623 1.594<br>C8 1.300 6.995 1.573<br>C9 0.824 6.267 2.656<br>C10 3.716 6.859 5.109<br>C11 3.057 8.345 0.387<br>C12 -0.557 5.686 2.608<br>C13 0.657 3.903 8.234<br>C14 1.107 4.347 9.501<br>C15 0.735 3.632 10.634<br>C16 -0.079 2.503 10.569<br>C17 -0.501 2.081 9.311<br>C18 -0.146 2.746 8.139<br>C19 1.961 5.564 9.717<br>C20 -0.446 1.755 11.810<br>C21 -0.634 2.155 6.849<br>H3 4.247 8.078 2.821<br>H4 0.677 7.079 0.681<br>H5 3.167 6.608 6.022<br>H6 4.159 7.851 5.246<br>H7 4.555 6.153 5.035<br>H8 3.846 9.059 0.642<br>H9 2.258 8.887 -0.129<br>H10 3.480 7.635 -0.337<br>H11 -0.908 5.597 1.575<br>H12 -1.284 6.327 3.131<br>H13 -0.599 4.693 3.064<br>H14 1.091 3.976 11.606<br>H15 -1.130 1.193 9.231<br>H16 2.331 5.995 8.784<br>H17 2.824 5.305 10.342<br>H18 1.411 6.339 10.265<br>H19 -1.256 1.042 11.636<br>H20 -0.753 2.434 12.612<br>H21 0.418 1.191 12.186<br>H22 -0.154 2.603 5.980<br>H23 -1.721 2.253 6.736<br>H24 -0.430 1.079 6.825<br>H46 -0.387 7.015 5.172<br>H47 1.088 7.230 6.178<br>N48 3.040 2.480 6.324<br>N49 2.893 3.202 4.157<br>C50 2.403 3.476 5.471<br>N51 4.169 1.919 5.530<br>C52 3.563 1.941 4.179<br>C53 3.319 2.487 7.739<br>C54 4.118 3.466 8.370<br>C55 4.465 3.280 9.707<br>C56 4.039 2.182 10.447<br>C57 3.230 1.243 9.808<br>C58 2.868 1.362 8.471<br>C59 4.604 4.721 7.708<br>C60 4.390 2.027 11.892<br>C61 2.020 0.267 7.893<br>C62 2.276 3.358 2.854<br>C63 3.079 3.944 1.858	N1 1.029 5.657 5.292<br>N2 1.159 4.768 7.396<br>C1 1.638 4.580 6.061<br>N4 0.551 6.657 6.275<br>C3 0.119 5.748 7.361<br>C4 1.401 6.243 4.030<br>C5 2.644 6.869 3.795<br>C6 2.842 7.514 2.576<br>C7 1.866 7.557 1.585<br>C8 0.653 6.918 1.837<br>C9 0.395 6.261 3.033<br>C10 3.803 6.867 4.753<br>C11 2.116 8.215 0.266<br>C12 -0.947 5.603 3.181<br>C13 0.845 3.848 8.476<br>C14 1.258 4.232 9.763<br>C15 0.855 3.459 10.849<br>C16 0.025 2.351 10.704<br>C17 -0.442 2.053 9.424<br>C18 -0.062 2.790 8.306<br>C19 2.051 5.477 10.027<br>C20 -0.341 1.500 11.879<br>C21 -0.619 2.431 6.965<br>H3 3.806 7.990 2.392<br>H4 -0.121 6.922 1.067<br>H5 3.946 7.841 5.238<br>H6 4.735 6.670 4.211<br>H7 3.697 6.109 5.530<br>H8 3.062 8.763 0.253<br>H9 1.312 8.913 0.009<br>H10 2.150 7.463 -0.533<br>H11 -1.141 4.961 2.315<br>H12 -1.752 6.348 3.198<br>H13 -1.026 4.984 4.079<br>H14 1.194 3.744 11.846<br>H15 -1.138 1.224 9.292<br>H16 2.639 5.789 9.160<br>H17 2.732 5.335 10.873<br>H18 1.391 6.313 10.302<br>H19 -1.331 1.048 11.761<br>H20 -0.335 2.072 12.812<br>H21 0.376 0.677 11.998<br>H22 0.166 2.077 6.290<br>H23 -1.105 3.281 6.466<br>H24 -1.367 1.638 7.053<br>H46 0.037 6.302 8.304<br>H47 -0.871 5.349 7.079<br>N48 3.125 2.581 6.345<br>N49 2.907 3.398 4.217<br>C50 2.476 3.625 5.563<br>N51 4.218 2.043 5.502<br>C52 3.577 2.135 4.169<br>C53 3.416 2.517 7.754<br>C54 4.212 3.465 8.432<br>C55 4.513 3.239 9.774<br>C56 4.053 2.125 10.467<br>C57 3.258 1.211 9.777<br>C58 2.927 1.378 8.439<br>C59 4.738 4.738 7.830<br>C60 4.350 1.926 11.919<br>C61 2.053 0.330 7.811<br>C62 2.264 3.592 2.928<br>C63 3.055 4.155 1.913

C64 2.583 4.006 0.562	C64 2.524 4.243 0.628
C65 1.337 3.477 0.220	C65 1.258 3.758 0.315
C66 0.611 2.825 1.210	C66 0.535 3.125 1.325
C67 1.065 2.735 2.527	C67 1.018 3.019 2.627
C68 4.460 4.451 2.149	C68 4.468 4.603 2.137
C69 0.803 3.619 -1.170	C69 0.685 3.929 -1.057
C70 0.236 1.965 3.510	C70 0.196 2.318 3.661
H71 5.091 4.035 10.187	H71 5.119 3.978 10.300
H72 2.871 0.379 10.370	H72 2.867 0.340 10.307
H73 5.690 4.714 7.555	H73 5.814 4.688 7.624
H74 4.403 5.587 8.348	H74 4.613 5.566 8.536
H75 4.120 4.898 6.748	H75 4.225 5.002 6.904
H76 5.198 2.700 12.192	H76 4.996 2.713 12.318
H77 4.694 1.000 12.124	H77 4.840 0.961 12.096
H78 3.520 2.250 12.523	H78 3.420 1.919 12.502
H79 1.147 0.098 8.533	H79 1.176 0.152 8.443
H80 2.567 -0.684 7.868	H80 2.579 -0.631 7.736
H81 1.665 0.491 6.883	H81 1.695 0.609 6.816
H82 3.190 4.480 -0.211	H82 3.130 4.700 -0.156
H83 -0.341 2.357 0.954	H83 -0.438 2.690 1.092
H84 4.516 4.965 3.112	H84 4.666 4.844 3.184
H85 4.790 5.150 1.374	H85 4.701 5.483 1.529
H86 5.194 3.631 2.163	H86 5.180 3.823 1.833
H87 0.027 2.880 -1.386	H87 -0.037 3.143 -1.298
H88 1.595 3.515 -1.920	H88 1.465 3.925 -1.825
H89 0.358 4.613 -1.312	H89 0.157 4.889 -1.137
H90 -0.599 2.569 3.894	H90 -0.147 3.014 4.433
H91 0.805 1.615 4.377	H91 0.750 1.522 4.178
H92 -0.213 1.090 3.029	H92 -0.686 1.856 3.210
H93 4.343 1.870 3.412	H93 4.339 2.097 3.382
H94 2.893 1.068 4.107	H94 2.900 1.268 4.070
H95 -2.184 5.859 6.185	H95 1.919 8.231 5.930
H96 -1.836 4.430 7.197	H96 2.427 7.044 7.186
C97 -1.466 5.060 6.389	H97 1.141 8.239 7.539
H98 -1.273 4.487 5.483	C98 4.530 0.624 5.840
C99 -0.478 6.583 8.004	H99 4.959 0.582 6.843
H100 -0.885 5.978 8.816	H100 3.614 0.037 5.789
H101 0.441 7.078 8.314	H101 5.255 0.267 5.105
H102 -1.216 7.320 7.680	H102 6.186 2.376 4.822
H103 6.149 2.229 4.887	H103 5.902 2.788 6.538
H104 5.213 3.728 5.168	C104 5.491 2.835 5.530
C105 5.431 2.728 5.542	H105 5.274 3.862 5.235
H106 5.816 2.752 6.561	C106 -0.605 7.442 5.754
C107 4.491 0.524 5.939	H107 -0.269 8.069 4.926
H108 4.906 0.531 6.949	H108 -1.383 6.755 5.425
H109 3.584 -0.077 5.898	H109 -0.967 8.066 6.575
H110 5.232 0.142 5.233	C110 1.597 7.613 6.767
**3-GrI**	**3-Ind**
Ru1 3.181 3.243 5.585	Ru1 6.541 8.737 11.226
Cl1 1.338 1.665 5.285	Cl1 5.952 6.382 11.708
Cl2 5.014 4.673 6.363	Cl2 7.792 10.773 10.889
P1 4.782 1.370 5.478	P1 7.603 8.003 9.110
N1 1.458 5.792 5.504	N1 4.819 8.844 13.805
N2 1.377 4.829 7.463	N2 6.972 8.792 14.221
C1 1.879 4.705 6.213	C1 6.056 8.958 13.240
N8 0.485 6.607 6.332	N8 4.985 8.337 15.252
C3 0.840 6.143 7.706	C3 6.384 8.749 15.538
C4 1.585 6.128 4.122	C4 3.549 9.389 13.444
C5 2.592 7.046 3.756	C5 3.378 10.788 13.554
C6 2.693 7.407 2.415	C6 2.131 11.325 13.277
C7 1.854 6.867 1.443	C7 1.051 10.520 12.903
C8 0.908 5.918 1.832	C8 1.254 9.150 12.792
C9 0.755 5.518 3.154	C9 2.493 8.553 13.039
C10 3.593 7.604 4.726	C10 4.514 11.692 13.923

C11 2.009 7.223 -0.002<br>C12 -0.222 4.418 3.458<br>C13 1.547 3.955 8.596<br>C14 2.580 4.198 9.509<br>C15 2.644 3.383 10.640<br>C16 1.713 2.378 10.881<br>C17 0.679 2.191 9.960<br>C18 0.559 2.979 8.823<br>C19 3.557 5.325 9.361<br>C20 1.792 1.516 12.105<br>C21 -0.621 2.828 7.913<br>C22 6.551 1.856 5.162<br>C23 7.560 0.706 5.261<br>C24 8.985 1.256 5.252<br>C25 9.241 2.113 4.017<br>C26 8.216 3.238 3.905<br>C27 6.792 2.687 3.899<br>C28 4.852 0.598 7.174<br>C29 5.607 1.420 8.224<br>C30 5.754 0.595 9.501<br>C31 4.398 0.153 10.039<br>C32 3.575 -0.578 8.983<br>C33 3.457 0.244 7.703<br>C34 4.286 -0.031 4.337<br>C35 4.658 -1.450 4.785<br>C36 4.047 -2.482 3.836<br>C37 4.464 -2.243 2.389<br>C38 4.122 -0.823 1.956<br>C39 4.761 0.195 2.896<br>C40 3.550 3.859 3.883<br>C41 3.221 3.445 2.532<br>C42 2.272 2.465 2.189<br>C43 2.046 2.147 0.859<br>C44 2.780 2.768 -0.151<br>C45 3.738 3.726 0.171<br>C46 3.944 4.072 1.498<br>H3 3.471 8.111 2.120<br>H4 0.296 5.436 1.068<br>H5 3.419 8.671 4.920<br>H6 4.601 7.527 4.301<br>H7 3.627 7.056 5.675<br>H8 2.667 8.084 -0.144<br>H9 1.042 7.447 -0.466<br>H10 2.438 6.375 -0.553<br>H11 -0.205 3.689 2.640<br>H12 -1.255 4.784 3.523<br>H13 0.021 3.859 4.368<br>H14 3.451 3.547 11.356<br>H15 -0.068 1.419 10.147<br>H16 3.673 5.661 8.325<br>H17 4.555 5.015 9.689<br>H18 3.262 6.173 9.995<br>H19 0.920 1.672 12.752<br>H20 2.691 1.725 12.692<br>H21 1.804 0.452 11.836<br>H22 -0.315 2.642 6.875<br>H23 -1.257 3.727 7.938<br>H24 -1.248 1.988 8.228<br>H25 6.730 2.547 6.002<br>H26 7.428 0.022 4.406<br>H27 7.396 0.107 6.168<br>H28 9.134 1.866 6.159<br>H29 9.706 0.431 5.309<br>H30 10.260 2.520 4.038<br>H31 9.176 1.477 3.119	C11 -0.274 11.141 12.590<br>C12 2.609 7.075 12.820<br>C13 8.405 8.896 14.122<br>C14 8.991 10.171 14.133<br>C15 10.377 10.245 14.061<br>C16 11.174 9.101 14.017<br>C17 10.556 7.855 14.080<br>C18 9.169 7.722 14.134<br>C19 8.175 11.424 14.219<br>C20 12.662 9.225 13.897<br>C21 8.548 6.362 14.222<br>C22 7.723 9.310 7.794<br>C23 8.486 8.887 6.533<br>C24 8.737 10.104 5.643<br>C25 7.434 10.817 5.293<br>C26 6.660 11.203 6.550<br>C27 6.397 9.983 7.428<br>C28 9.384 7.561 9.442<br>C29 10.344 8.731 9.677<br>C30 11.776 8.202 9.733<br>C31 11.939 7.150 10.825<br>C32 10.922 6.021 10.691<br>C33 9.500 6.567 10.602<br>C34 6.889 6.426 8.387<br>C35 7.855 5.479 7.662<br>C36 7.145 4.165 7.331<br>C37 5.881 4.387 6.508<br>C38 4.943 5.371 7.200<br>C39 5.665 6.681 7.500<br>H3 1.998 12.406 13.329<br>H4 0.431 8.513 12.465<br>H5 4.848 11.560 14.963<br>H6 4.225 12.741 13.811<br>H7 5.388 11.520 13.282<br>H8 -0.698 11.644 13.468<br>H9 -1.002 10.400 12.242<br>H10 -0.162 11.907 11.811<br>H11 2.051 6.799 11.918<br>H12 2.150 6.509 13.644<br>H13 3.642 6.743 12.678<br>H14 10.851 11.227 14.045<br>H15 11.169 6.953 14.079<br>H16 7.635 11.591 13.278<br>H17 8.820 12.291 14.389<br>H18 7.445 11.395 15.038<br>H19 13.058 9.978 14.588<br>H20 12.939 9.544 12.884<br>H21 13.167 8.275 14.095<br>H22 7.785 6.205 13.448<br>H23 8.073 6.192 15.201<br>H24 9.309 5.584 14.105<br>H25 8.318 10.079 8.314<br>H26 7.895 8.142 5.974<br>H27 9.442 8.406 6.786<br>H28 9.404 10.801 6.178<br>H29 9.270 9.799 4.733<br>H30 7.637 11.701 4.675<br>H31 6.811 10.144 4.680<br>H32 5.710 11.687 6.286<br>H33 7.237 11.941 7.132<br>H34 5.862 10.283 8.337<br>H35 5.746 9.281 6.886<br>H36 9.718 7.051 8.523<br>H37 10.088 9.238 10.622<br>H38 10.259 9.490 8.888

H32 8.392 3.831 2.998<br>H33 8.324 3.927 4.758<br>H34 6.072 3.515 3.855<br>H35 6.644 2.075 2.996<br>H36 5.410 -0.345 7.037<br>H37 5.053 2.353 8.429<br>H38 6.599 1.724 7.867<br>H39 6.375 -0.289 9.282<br>H40 6.297 1.173 10.261<br>H41 3.844 1.051 10.350<br>H42 4.521 -0.472 10.934<br>H43 2.573 -0.813 9.370<br>H44 4.050 -1.545 8.745<br>H45 2.860 -0.290 6.952<br>H46 2.901 1.176 7.911<br>H47 3.185 0.045 4.341<br>H48 5.755 -1.564 4.797<br>H49 4.308 -1.651 5.806<br>H50 4.328 -3.493 4.158<br>H51 2.949 -2.424 3.912<br>H52 5.551 -2.396 2.294<br>H53 3.988 -2.978 1.727<br>H54 3.027 -0.684 1.967<br>H55 4.449 -0.635 0.924<br>H56 4.538 1.215 2.558<br>H57 5.855 0.078 2.849<br>H58 4.228 4.730 3.887<br>H59 1.726 1.960 2.984<br>H60 1.307 1.390 0.605<br>H61 2.611 2.497 -1.191<br>H62 4.323 4.201 -0.614<br>H63 4.685 4.829 1.757<br>H114 1.595 6.830 8.119<br>H115 -0.052 6.124 8.344<br>C116 0.693 8.070 6.168<br>H117 -0.002 8.584 6.837<br>H118 1.725 8.311 6.425<br>H119 0.488 8.340 5.129<br>C120 -0.919 6.240 5.980<br>H121 -1.084 6.493 4.931<br>H122 -1.048 5.168 6.144<br>H123 -1.594 6.816 6.619	H39 12.039 7.766 8.755<br>H40 12.476 9.032 9.898<br>H41 11.785 7.637 11.800<br>H42 12.961 6.750 10.829<br>H43 11.007 5.325 11.538<br>H44 11.136 5.431 9.784<br>H45 8.774 5.746 10.515<br>H46 9.250 7.099 11.537<br>H47 6.533 5.903 9.290<br>H48 8.220 5.949 6.734<br>H49 8.739 5.259 8.274<br>H50 7.835 3.493 6.805<br>H51 6.880 3.663 8.276<br>H52 6.158 4.794 5.522<br>H53 5.374 3.433 6.319<br>H54 4.581 4.935 8.148<br>H55 4.058 5.562 6.578<br>H56 4.979 7.400 7.967<br>H57 5.989 7.133 6.548<br>H101 6.377 9.736 16.027<br>H102 6.868 8.011 16.187<br>C103 4.890 9.224 10.509<br>C104 4.268 10.576 10.389<br>C105 4.749 11.853 10.625<br>H106 5.755 11.997 11.008<br>C107 3.936 12.956 10.329<br>H108 4.313 13.960 10.510<br>C109 2.675 12.779 9.783<br>H110 2.064 13.644 9.533<br>C111 2.185 11.492 9.527<br>H112 1.217 11.366 9.049<br>C113 2.974 10.402 9.847<br>C114 2.750 8.953 9.658<br>C115 3.897 8.293 9.998<br>H116 4.027 7.216 10.025<br>C117 1.497 8.338 9.248<br>C118 0.263 8.927 9.564<br>H119 0.242 9.861 10.119<br>C120 -0.928 8.307 9.213<br>H121 -1.876 8.775 9.470<br>C122 -0.908 7.093 8.533<br>H123 -1.841 6.612 8.249<br>C124 0.309 6.496 8.215<br>H125 0.328 5.550 7.679<br>C126 1.500 7.109 8.572<br>H127 2.452 6.651 8.305<br>H128 2.997 8.653 15.879<br>H129 4.110 10.014 16.202<br>H130 4.214 8.519 17.189<br>C131 4.866 6.844 15.224<br>H132 3.827 6.588 15.015<br>H133 5.505 6.459 14.422<br>H134 5.164 6.460 16.204<br>C135 4.005 8.928 16.196
**3-GrII**	**3-GrIIdim**
N1 5.948 1.142 1.619<br>N2 5.143 0.654 3.598<br>C1 4.959 1.435 2.513<br>N4 6.973 0.200 2.294<br>C3 6.156 -0.357 3.398<br>C4 5.982 1.173 0.195<br>C5 5.117 0.306 -0.509<br>C6 5.257 0.222 -1.886<br>C7 6.218 0.967 -2.575	N1 5.897 1.098 1.673<br>N2 5.108 0.706 3.681<br>C1 4.923 1.448 2.565<br>N4 6.910 0.173 2.369<br>C3 6.091 -0.337 3.497<br>C4 5.918 1.106 0.247<br>C5 5.023 0.251 -0.434<br>C6 5.124 0.165 -1.815<br>C7 6.081 0.892 -2.529

C8 7.021 1.845 -1.855<br>C9 6.916 1.985 -0.471<br>C10 4.042 -0.471 0.190<br>C11 6.328 0.852 -4.063<br>C12 7.751 3.031 0.208<br>C13 4.439 0.547 4.853<br>C14 3.362 -0.344 4.952<br>C15 2.869 -0.620 6.228<br>C16 3.449 -0.084 7.375<br>C17 4.517 0.800 7.230<br>C18 5.038 1.125 5.984<br>C19 2.778 -1.029 3.756<br>C20 2.996 -0.490 8.744<br>C21 6.241 2.012 5.881<br>H3 4.587 -0.430 -2.446<br>H4 7.736 2.472 -2.389<br>H5 4.435 -1.162 0.951<br>H6 3.473 -1.070 -0.527<br>H7 3.334 0.196 0.706<br>H8 6.476 -0.189 -4.373<br>H9 7.155 1.448 -4.460<br>H10 5.401 1.198 -4.540<br>H11 7.761 3.939 -0.407<br>H12 8.803 2.727 0.302<br>H13 7.362 3.313 1.193<br>H14 2.032 -1.313 6.327<br>H15 4.978 1.233 8.120<br>H16 2.315 -0.304 3.074<br>H17 2.015 -1.751 4.058<br>H18 3.537 -1.582 3.182<br>H19 3.728 -1.163 9.208<br>H20 2.035 -1.016 8.721<br>H21 2.896 0.377 9.408<br>H22 6.121 2.794 5.120<br>H23 7.148 1.438 5.633<br>H24 6.442 2.500 6.840<br>H46 5.724 -1.319 3.081<br>H47 6.773 -0.502 4.291<br>Ru48 3.732 3.131 2.380<br>Cl49 1.676 1.870 2.150<br>Cl50 5.790 4.316 3.095<br>N51 2.590 6.051 2.129<br>N52 2.235 5.168 4.086<br>C53 2.698 4.915 2.849<br>C54 1.922 6.594 4.292<br>C55 2.715 6.212 0.715<br>C56 3.819 6.909 0.205<br>C57 3.874 7.129 -1.170<br>C58 2.864 6.694 -2.026<br>C59 1.769 6.026 -1.480<br>C60 1.668 5.777 -0.115<br>C61 4.918 7.387 1.103<br>C62 2.943 6.935 -3.502<br>C63 0.489 5.031 0.430<br>C64 1.886 4.234 5.114<br>C65 2.735 4.044 6.209<br>C66 2.279 3.246 7.259<br>C67 1.014 2.668 7.247<br>C68 0.186 2.899 6.150<br>C69 0.588 3.694 5.084<br>C70 4.058 4.743 6.311<br>C71 0.521 1.831 8.386<br>C72 -0.367 4.002 3.972<br>C73 4.150 3.615 0.639<br>C74 3.651 3.301 -0.691	C8 6.919 1.753 -1.829<br>C9 6.851 1.898 -0.443<br>C10 3.952 -0.506 0.292<br>C11 6.153 0.777 -4.019<br>C12 7.722 2.931 0.210<br>C13 4.423 0.656 4.952<br>C14 3.328 -0.207 5.101<br>C15 2.868 -0.451 6.395<br>C16 3.486 0.098 7.515<br>C17 4.560 0.966 7.323<br>C18 5.057 1.251 6.057<br>C19 2.687 -0.896 3.937<br>C20 3.066 -0.275 8.905<br>C21 6.272 2.117 5.913<br>H3 4.428 -0.476 -2.357<br>H4 7.633 2.366 -2.381<br>H5 4.349 -1.168 1.076<br>H6 3.384 -1.132 -0.402<br>H7 3.243 0.177 0.787<br>H8 6.298 -0.263 -4.333<br>H9 6.969 1.376 -4.436<br>H10 5.214 1.120 -4.473<br>H11 7.755 3.827 -0.421<br>H12 8.765 2.598 0.304<br>H13 7.348 3.242 1.193<br>H14 2.024 -1.129 6.529<br>H15 5.047 1.413 8.191<br>H16 2.149 -0.179 3.304<br>H17 1.977 -1.653 4.281<br>H18 3.422 -1.400 3.295<br>H19 3.805 -0.946 9.362<br>H20 2.101 -0.790 8.920<br>H21 2.995 0.606 9.553<br>H22 6.167 2.856 5.108<br>H23 7.173 1.519 5.707<br>H24 6.470 2.657 6.843<br>H46 5.633 -1.296 3.207<br>H47 6.716 -0.475 4.386<br>Ru48 3.729 3.139 2.379<br>Cl49 1.653 1.914 2.121<br>Cl50 5.801 4.314 3.084<br>N51 2.537 6.087 2.109<br>N52 2.131 5.214 4.028<br>C53 2.669 4.926 2.811<br>C54 1.940 7.078 2.881<br>C55 1.691 6.530 4.087<br>C56 2.737 6.286 0.703<br>C57 3.872 6.970 0.258<br>C58 3.985 7.203 -1.112<br>C59 3.002 6.794 -2.012<br>C60 1.872 6.141 -1.520<br>C61 1.712 5.878 -0.164<br>C62 4.928 7.431 1.215<br>C63 3.143 7.054 -3.481<br>C64 0.497 5.164 0.343<br>C65 1.803 4.288 5.080<br>C66 2.674 4.132 6.161<br>C67 2.243 3.341 7.227<br>C68 0.991 2.733 7.231<br>C69 0.150 2.924 6.135<br>C70 0.521 3.716 5.056<br>C71 3.982 4.857 6.225<br>C72 0.522 1.905 8.388<br>C73 -0.445 3.987 3.945<br>C74 4.166 3.616 0.641

C75 2.521 2.512 -0.979<br>C76 2.144 2.274 -2.291<br>C77 2.886 2.802 -3.348<br>C78 4.008 3.581 -3.086<br>C79 4.378 3.836 -1.773<br>H81 4.736 7.655 -1.586<br>H82 0.977 5.669 -2.140<br>H83 4.570 8.170 1.790<br>H84 5.740 7.811 0.518<br>H85 5.315 6.575 1.727<br>H86 3.977 7.087 -3.831<br>H87 2.373 7.827 -3.791<br>H88 2.525 6.090 -4.063<br>H89 -0.221 4.795 -0.370<br>H90 -0.052 5.606 1.193<br>H91 0.790 4.083 0.899<br>H92 2.925 3.101 8.127<br>H93 -0.820 2.477 6.142<br>H94 4.586 4.790 5.352<br>H95 4.704 4.240 7.040<br>H96 3.928 5.776 6.665<br>H97 -0.528 2.048 8.616<br>H98 1.107 1.996 9.298<br>H99 0.579 0.762 8.141<br>H100 -0.055 3.512 3.042<br>H101 -0.431 5.080 3.773<br>H102 -1.373 3.649 4.219<br>H103 5.023 4.289 0.592<br>H104 1.944 2.101 -0.155<br>H105 1.263 1.669 -2.494<br>H106 2.585 2.608 -4.376<br>H107 4.591 4.001 -3.904<br>H108 5.243 4.467 -1.567<br>H109 8.040 -0.382 0.567<br>H110 6.671 -1.446 0.999<br>H111 8.176 -1.482 1.977<br>C112 8.077 1.057 2.824<br>H113 8.641 1.452 1.980<br>H114 7.634 1.883 3.388<br>H115 8.721 0.438 3.457<br>C116 7.499 -0.853 1.390<br>C117 1.898 7.123 2.869<br>H117 2.710 7.058 4.901<br>H118 0.968 6.702 4.820<br>H119 0.879 7.245 2.471<br>H120 2.425 8.075 2.746	C75 3.658 3.332 -0.691<br>C76 2.514 2.568 -0.991<br>C77 2.130 2.360 -2.306<br>C78 2.881 2.891 -3.355<br>C79 4.020 3.642 -3.082<br>C80 4.395 3.868 -1.765<br>H81 1.748 8.062 2.478<br>H82 1.231 6.926 4.982<br>H83 4.868 7.723 -1.486<br>H84 1.099 5.811 -2.215<br>H85 4.537 8.181 1.914<br>H86 5.767 7.883 0.678<br>H87 5.311 6.602 1.826<br>H88 4.193 7.166 -3.771<br>H89 2.622 7.974 -3.773<br>H90 2.710 6.236 -4.069<br>H91 -0.217 4.993 -0.469<br>H92 -0.013 5.736 1.129<br>H93 0.755 4.186 0.777<br>H94 2.898 3.225 8.092<br>H95 -0.843 2.473 6.139<br>H96 4.593 4.704 5.326<br>H97 4.558 4.535 7.099<br>H98 3.827 5.941 6.314<br>H99 -0.525 2.118 8.631<br>H100 1.120 2.087 9.288<br>H101 0.584 0.834 8.155<br>H102 -0.064 3.606 2.991<br>H103 -0.631 5.062 3.827<br>H104 -1.405 3.501 4.143<br>H105 5.053 4.271 0.601<br>H106 1.931 2.151 -0.173<br>H107 1.238 1.775 -2.518<br>H108 2.575 2.720 -4.385<br>H109 4.612 4.061 -3.894<br>H110 5.274 4.477 -1.551<br>H111 7.957 -0.477 0.655<br>H112 6.577 -1.508 1.128<br>H113 8.088 -1.534 2.097<br>C114 8.031 1.028 2.869<br>H115 8.599 1.386 2.011<br>H116 7.604 1.879 3.408<br>H117 8.666 0.418 3.518<br>C118 7.416 -0.915 1.496
**3-Hov**	**3-Hov-TS**
Ru1 12.850 5.811 5.358<br>Cl1 10.558 5.379 5.970<br>Cl2 15.210 6.151 5.420<br>O1 13.298 3.664 5.970<br>C22 12.829 4.971 3.717<br>H27 12.636 5.465 2.758<br>C23 13.375 3.121 7.322<br>H28 14.247 2.450 7.337<br>C24 13.627 4.301 8.232<br>H29 14.521 4.857 7.927<br>H30 13.765 3.945 9.259<br>H31 12.763 4.981 8.229<br>C25 12.093 2.386 7.646<br>H32 11.904 1.553 6.961<br>H33 11.246 3.079 7.583<br>H34 12.145 1.983 8.663<br>C26 13.377 2.881 4.872	Ru1 12.739 5.690 5.597<br>Cl1 10.549 5.455 6.474<br>Cl2 15.018 6.027 6.046<br>O1 11.058 2.949 4.815<br>C22 12.883 4.811 4.023<br>H27 12.827 5.233 3.014<br>C23 9.978 2.183 4.255<br>H28 10.075 1.132 4.567<br>C24 8.709 2.756 4.836<br>H29 8.717 2.690 5.928<br>H30 7.837 2.215 4.453<br>H31 8.615 3.815 4.568<br>C25 10.050 2.285 2.744<br>H32 10.962 1.819 2.353<br>H33 10.061 3.346 2.453<br>H34 9.189 1.799 2.271<br>C26 12.313 2.482 4.656

C27 13.689 1.528 4.864<br>H35 13.886 0.988 5.784<br>C28 13.754 0.868 3.640<br>H36 14.003 -0.191 3.632<br>C29 13.509 1.526 2.435<br>H37 13.566 0.983 1.496<br>C30 13.198 2.873 2.450<br>H38 13.005 3.417 1.525<br>C31 13.128 3.574 3.665<br>N1 11.681 8.099 3.810<br>N2 12.402 8.626 5.827<br>C1 12.349 7.615 4.910<br>N30 11.102 9.450 4.148<br>C3 12.002 9.884 5.265<br>C4 11.777 7.689 2.446<br>C5 12.992 7.917 1.766<br>C6 13.086 7.501 0.443<br>C7 12.030 6.858 -0.205<br>C8 10.861 6.610 0.513<br>C9 10.709 7.001 1.842<br>C10 14.194 8.500 2.445<br>C11 12.165 6.401 -1.624<br>C12 9.463 6.609 2.577<br>C13 13.028 8.535 7.124<br>C14 14.400 8.799 7.244<br>C15 14.995 8.593 8.486<br>C16 14.261 8.168 9.591<br>C17 12.881 8.012 9.454<br>C18 12.236 8.202 8.236<br>C19 15.214 9.355 6.116<br>C20 14.930 7.884 10.900<br>C21 10.743 8.104 8.157<br>H3 14.018 7.671 -0.096<br>H4 10.045 6.073 0.030<br>H5 13.980 9.429 2.990<br>H6 14.979 8.726 1.718<br>H7 14.613 7.789 3.176<br>H8 12.915 6.985 -2.167<br>H9 11.214 6.474 -2.162<br>H10 12.478 5.351 -1.663<br>H11 9.078 5.667 2.175<br>H12 8.656 7.344 2.447<br>H13 9.641 6.457 3.649<br>H14 16.066 8.767 8.588<br>H15 12.286 7.743 10.327<br>H16 16.267 9.078 6.225<br>H17 15.165 10.454 6.113<br>H18 14.891 8.983 5.141<br>H19 14.285 8.145 11.747<br>H20 15.872 8.430 11.003<br>H21 15.161 6.814 10.989<br>H22 10.417 7.271 7.522<br>H23 10.301 9.030 7.760<br>H24 10.317 7.951 9.153<br>H72 12.841 10.455 4.835<br>H73 11.444 10.508 5.972<br>H74 10.482 9.988 2.206<br>H75 12.134 10.516 2.638<br>H76 10.712 11.346 3.354<br>C77 9.706 9.243 4.653<br>H78 9.099 8.863 3.832<br>H79 9.739 8.508 5.462<br>H80 9.321 10.204 5.005<br>C81 11.112 10.392 3.001	C27 12.686 1.153 4.867<br>H35 11.946 0.421 5.178<br>C28 14.011 0.779 4.700<br>H36 14.295 -0.256 4.874<br>C29 14.982 1.711 4.328<br>H37 16.018 1.405 4.211<br>C30 14.620 3.029 4.121<br>H38 15.365 3.775 3.853<br>C31 13.280 3.426 4.269<br>N1 11.436 7.799 4.045<br>N2 12.410 8.607 5.858<br>C1 12.259 7.484 5.109<br>N30 10.836 9.157 4.292<br>C3 11.845 9.764 5.215<br>C4 11.440 7.250 2.725<br>C5 12.584 7.444 1.920<br>C6 12.601 6.867 0.656<br>C7 11.532 6.103 0.179<br>C8 10.428 5.920 1.006<br>C9 10.355 6.473 2.284<br>C10 13.805 8.156 2.419<br>C11 11.602 5.466 -1.173<br>C12 9.154 6.185 3.131<br>C13 13.184 8.793 7.066<br>C14 14.527 9.178 6.953<br>C15 15.254 9.334 8.127<br>C16 14.683 9.129 9.383<br>C17 13.329 8.813 9.451<br>C18 12.550 8.653 8.308<br>C19 15.188 9.413 5.628<br>C20 15.509 9.250 10.626<br>C21 11.080 8.394 8.435<br>H3 13.481 7.002 0.027<br>H4 9.596 5.309 0.655<br>H5 13.588 9.125 2.890<br>H6 14.508 8.343 1.603<br>H7 14.327 7.550 3.174<br>H8 11.996 6.161 -1.923<br>H9 10.621 5.117 -1.508<br>H10 12.275 4.600 -1.156<br>H11 8.697 5.241 2.820<br>H12 8.373 6.950 3.010<br>H13 9.412 6.099 4.194<br>H14 16.305 9.617 8.060<br>H15 12.856 8.696 10.426<br>H16 16.228 9.718 5.770<br>H17 14.700 10.209 5.047<br>H18 15.195 8.496 5.029<br>H19 14.901 9.537 11.490<br>H20 16.311 9.986 10.508<br>H21 15.984 8.290 10.866<br>H22 10.782 7.444 7.977<br>H23 10.488 9.195 7.968<br>H24 10.787 8.362 9.488<br>C72 10.647 9.948 3.049<br>H73 12.579 10.326 4.617<br>H74 11.347 10.432 5.926<br>H75 11.605 10.062 2.539<br>H76 9.940 9.426 2.402<br>H77 10.247 10.923 3.338<br>C78 9.529 8.961 5.003<br>H79 8.835 8.478 4.316<br>H80 9.701 8.312 5.867<br>H81 9.153 9.942 5.307

3-Hov-a	3-GrI_P(Cy)$_3$
Ru1 12.486 5.818 5.511	Ru1 2.296 3.269 5.330
Cl1 10.300 5.625 6.422	Cl1 0.073 2.545 4.919
Cl2 14.846 5.741 5.438	Cl2 4.471 3.081 6.186
O1 11.023 3.244 2.036	N1 1.196 6.019 5.472
C22 12.144 4.871 3.959	N2 1.414 5.014 7.430
H27 11.835 5.303 3.005	C1 1.679 4.887 6.098
C23 10.521 2.467 0.931	N7 0.382 6.799 6.460
H28 9.995 1.589 1.334	C3 0.976 6.340 7.759
C24 9.523 3.342 0.214	C4 1.618 6.626 4.251
H29 8.742 3.689 0.899	C5 2.899 7.213 4.207
H30 9.050 2.784 -0.600	C6 3.326 7.762 3.002
H31 10.030 4.214 -0.215	C7 2.533 7.726 1.855
C25 11.672 2.055 0.036	C8 1.287 7.103 1.927
H32 12.407 1.437 0.562	C9 0.805 6.537 3.104
H33 12.186 2.953 -0.330	C10 3.848 7.162 5.366
H34 11.306 1.489 -0.828	C11 3.017 8.304 0.562
C26 11.625 2.616 3.062	C12 -0.502 5.803 3.077
C27 11.679 1.228 3.214	C13 1.772 4.074 8.466
H35 11.258 0.567 2.464	C14 3.042 4.164 9.053
C28 12.267 0.670 4.340	C15 3.372 3.224 10.024
H36 12.290 -0.413 4.436	C16 2.478 2.234 10.426
C29 12.832 1.466 5.337	C17 1.202 2.221 9.865
H37 13.303 1.016 6.205	C18 0.817 3.137 8.890
C30 12.804 2.835 5.185	C19 4.012 5.249 8.700
H38 13.301 3.456 5.934	C20 2.874 1.206 11.440
C31 12.207 3.447 4.059	C21 -0.590 3.136 8.372
N1 11.586 8.141 3.908	C40 2.755 3.792 3.625
N2 12.442 8.662 5.876	C41 3.096 2.802 2.644
C1 12.205 7.622 5.022	C42 2.908 1.424 2.876
N30 11.242 9.579 4.170	C43 3.303 0.497 1.929
C3 12.238 9.928 5.236	C44 3.879 0.928 0.733
C4 11.520 7.642 2.574	C45 4.059 2.286 0.481
C5 12.701 7.638 1.800	C46 3.670 3.219 1.428
C6 12.614 7.190 0.490	H3 4.317 8.213 2.952
C7 11.404 6.760 -0.064	H4 0.677 7.032 1.025
C8 10.274 6.729 0.747	H5 4.701 7.826 5.200
C9 10.307 7.132 2.082	H6 4.245 6.143 5.494
C10 14.034 8.014 2.375	H7 3.394 7.446 6.324
C11 11.351 6.312 -1.492	H8 3.983 8.804 0.675
C12 9.097 6.903 2.939	H9 2.301 9.030 0.161
C13 13.092 8.581 7.162	H10 3.131 7.520 -0.197
C14 14.490 8.674 7.229	H11 -0.665 5.374 2.084
C15 15.085 8.555 8.482	H12 -1.354 6.472 3.262
C16 14.334 8.379 9.642	H13 -0.531 4.976 3.796
C17 12.943 8.373 9.540	H14 4.362 3.267 10.479
C18 12.294 8.487 8.315	H15 0.478 1.481 10.204
C19 15.337 8.968 6.029	H16 4.069 5.413 7.620
C20 14.998 8.209 10.974	H17 5.020 4.987 9.034
C21 10.799 8.573 8.262	H18 3.748 6.198 9.192
H3 13.516 7.173 -0.122	H19 2.025 0.906 12.062
H4 9.335 6.351 0.343	H20 3.670 1.571 12.097
H5 14.029 8.981 2.897	H21 3.249 0.301 10.945
H6 14.793 8.074 1.591	H22 -0.634 2.931 7.295
H7 14.371 7.263 3.107	H23 -1.087 4.099 8.558
H8 11.803 7.058 -2.157	H24 -1.183 2.370 8.879
H9 10.324 6.136 -1.828	H58 2.882 4.836 3.324
H10 11.916 5.382 -1.633	H59 2.429 1.080 3.795
H11 8.662 5.929 2.687	H60 3.157 -0.564 2.113
H12 8.308 7.646 2.762	H61 4.185 0.195 -0.010
H13 9.337 6.874 4.008	H62 4.506 2.611 -0.455
H14 16.172 8.607 8.551	H63 3.809 4.285 1.248
H15 12.342 8.290 10.446	H64 1.802 7.019 8.023
H16 16.357 8.603 6.178	H65 0.212 6.349 8.544

H17 15.406 10.053 5.859<br>H18 14.962 8.487 5.121<br>H19 14.407 8.662 11.777<br>H20 15.998 8.653 10.988<br>H21 15.109 7.145 11.216<br>H22 10.358 7.744 7.696<br>H23 10.470 9.519 7.806<br>H24 10.379 8.545 9.272<br>H72 13.144 10.321 4.747<br>H73 11.815 10.680 5.912<br>H74 10.656 10.112 2.214<br>H75 12.390 10.366 2.574<br>H76 11.158 11.468 3.276<br>C77 9.846 9.639 4.712<br>H78 9.163 9.299 3.933<br>H79 9.786 8.979 5.581<br>H80 9.629 10.675 4.989<br>C81 11.375 10.442 2.967	H66 0.080 8.529 5.291<br>H67 1.534 8.573 6.333<br>H68 -0.098 8.744 7.063<br>C67 -1.047 6.354 6.351<br>H69 -1.419 6.642 5.368<br>H70 -1.078 5.267 6.460<br>H71 -1.621 6.848 7.141<br>C71 0.487 8.269 6.271
**3-Ind_P(Cy)$_3$**	**3-GrII_Imes/SIMes**
Ru1 6.415 9.135 11.427<br>Cl1 6.967 6.975 10.725<br>Cl2 6.774 11.485 11.718<br>N1 4.857 9.505 13.957<br>N2 6.971 8.895 14.215<br>C1 5.986 9.064 13.289<br>N7 5.280 9.935 15.331<br>C3 6.488 9.080 15.556<br>C4 3.467 9.313 13.682<br>C5 2.691 10.370 13.180<br>C6 1.339 10.123 12.935<br>C7 0.754 8.884 13.175<br>C8 1.563 7.846 13.646<br>C9 2.914 8.029 13.896<br>C10 3.248 11.723 12.852<br>C11 -0.698 8.634 12.915<br>C12 3.755 6.854 14.294<br>C13 8.291 8.364 13.974<br>C14 9.382 9.243 13.899<br>C15 10.636 8.695 13.637<br>C16 10.823 7.324 13.476<br>C17 9.724 6.480 13.629<br>C18 8.449 6.972 13.886<br>C19 9.248 10.716 14.134<br>C20 12.173 6.764 13.152<br>C21 7.305 6.020 14.047<br>H3 0.729 10.928 12.525<br>H4 1.127 6.856 13.786<br>H5 3.055 12.445 13.658<br>H6 2.750 12.120 11.961<br>H7 4.323 11.701 12.642<br>H8 -1.245 8.497 13.857<br>H9 -0.836 7.721 12.323<br>H10 -1.163 9.464 12.374<br>H11 3.157 5.939 14.304<br>H12 4.201 6.949 15.294<br>H13 4.586 6.696 13.593<br>H14 11.494 9.363 13.566<br>H15 9.863 5.402 13.545<br>H16 8.600 11.194 13.389<br>H17 10.226 11.203 14.086<br>H18 8.835 10.935 15.129<br>H19 12.339 5.802 13.648<br>H20 12.976 7.447 13.445<br>H21 12.266 6.591 12.073	N1 6.316 0.651 1.478<br>N2 5.718 0.691 3.608<br>C1 5.372 1.132 2.364<br>N4 7.188 -0.334 2.199<br>C3 7.034 0.120 3.620<br>C4 6.723 1.181 0.216<br>C5 6.352 0.526 -0.972<br>C6 6.732 1.111 -2.180<br>C7 7.434 2.312 -2.233<br>C8 7.754 2.954 -1.033<br>C9 7.404 2.418 0.200<br>C10 5.516 -0.718 -1.001<br>C11 7.796 2.940 -3.543<br>C12 7.651 3.212 1.447<br>C13 5.017 0.963 4.840<br>C14 4.078 0.030 5.308<br>C15 3.388 0.341 6.476<br>C16 3.625 1.519 7.183<br>C17 4.617 2.383 6.722<br>C18 5.335 2.125 5.559<br>C19 3.851 -1.288 4.632<br>C20 2.832 1.845 8.410<br>C21 6.429 3.055 5.130<br>H3 6.444 0.621 -3.110<br>H4 8.275 3.911 -1.063<br>H5 4.840 -0.784 -0.141<br>H6 6.131 -1.628 -1.050<br>H7 4.894 -0.725 -1.901<br>H8 7.718 2.228 -4.370<br>H9 8.815 3.339 -3.526<br>H10 7.128 3.781 -3.765<br>H11 8.343 4.036 1.254<br>H12 8.068 2.620 2.271<br>H13 6.711 3.651 1.817<br>H14 2.649 -0.366 6.852<br>H15 4.838 3.291 7.283<br>H16 3.463 -1.171 3.612<br>H17 3.126 -1.885 5.193<br>H18 4.778 -1.878 4.582<br>H19 3.385 2.504 9.087<br>H20 2.552 0.941 8.961<br>H21 1.902 2.362 8.142<br>H22 6.357 3.302 4.067<br>H23 7.424 2.629 5.332<br>H24 6.369 3.998 5.681

H22 6.702 5.989 13.130<br>H23 6.646 6.286 14.884<br>H24 7.672 5.006 14.232<br>H49 7.207 9.610 16.191<br>H50 6.171 8.143 16.040<br>C51 4.709 9.129 10.667<br>C52 4.270 10.036 9.585<br>C53 4.873 11.125 8.983<br>H54 5.848 11.474 9.313<br>C55 4.199 11.777 7.936<br>H56 4.662 12.633 7.454<br>C57 2.964 11.327 7.506<br>H58 2.461 11.826 6.681<br>C59 2.348 10.218 8.116<br>H60 1.393 9.856 7.744<br>C61 2.992 9.598 9.165<br>C62 2.635 8.408 9.974<br>C63 3.665 8.154 10.837<br>H64 3.713 7.321 11.527<br>C65 1.407 7.636 9.862<br>C66 0.189 8.254 9.546<br>H67 0.155 9.333 9.404<br>C68 -0.981 7.510 9.470<br>H69 -1.921 8.003 9.231<br>C70 -0.954 6.139 9.708<br>H71 -1.869 5.555 9.638<br>C72 0.249 5.514 10.034<br>H73 0.275 4.442 10.213<br>C74 1.418 6.255 10.112<br>H75 2.363 5.762 10.333<br>H76 4.752 11.956 15.044<br>H77 6.378 11.524 14.450<br>H78 6.074 11.680 16.230<br>C79 4.234 9.728 16.367<br>H80 3.364 10.338 16.116<br>H81 3.955 8.675 16.405<br>H82 4.657 10.047 17.323<br>C83 5.656 11.388 15.262	H46 7.115 -0.738 4.298<br>H47 7.826 0.854 3.838<br>Ru48 3.707 1.959 1.998<br>Cl49 2.715 -0.129 1.463<br>Cl50 3.876 3.999 3.141<br>C51 3.954 2.610 0.294<br>C52 2.841 3.153 -0.430<br>C53 1.516 3.045 0.044<br>C54 0.476 3.628 -0.655<br>C55 0.735 4.318 -1.840<br>C56 2.035 4.423 -2.330<br>C57 3.082 3.845 -1.632<br>H58 4.933 2.698 -0.187<br>H59 1.301 2.482 0.954<br>H60 -0.542 3.543 -0.286<br>H61 -0.086 4.772 -2.389<br>H62 2.226 4.959 -3.256<br>H63 4.106 3.922 -1.999<br>H64 6.664 -1.968 0.967<br>H65 5.556 -1.673 2.339<br>H66 7.178 -2.405 2.631<br>C67 8.601 -0.308 1.740<br>H68 8.636 -0.606 0.690<br>H69 9.005 0.699 1.857<br>H70 9.158 -1.019 2.355<br>C71 6.601 -1.704 2.022
**P(Cy)$_3$**	
P1 4.896 1.427 5.637<br>C22 6.707 1.841 5.362<br>C23 7.757 0.736 5.476<br>C24 9.168 1.319 5.432<br>C25 9.383 2.137 4.163<br>C26 8.331 3.234 4.030<br>C27 6.919 2.654 4.080<br>C28 4.943 0.711 7.371<br>C29 5.448 1.736 8.390<br>C30 5.397 1.187 9.813<br>C31 3.995 0.715 10.181<br>C32 3.491 -0.312 9.176<br>C33 3.535 0.245 7.756<br>C34 4.497 -0.119 4.633<br>C35 5.098 -1.470 5.032<br>C36 4.488 -2.599 4.202<br>C37 4.672 -2.352 2.708<br>C38 4.094 -1.000 2.302<br>C39 4.702 0.125 3.135<br>H25 6.886 2.549 6.192<br>H26 7.638 0.034 4.634<br>H27 7.615 0.150 6.395<br>H28 9.316 1.966 6.313<br>H29 9.915 0.518 5.506<br>H30 10.393 2.567 4.146	

H31 9.315 1.467 3.290<br>H32 8.479 3.799 3.100<br>H33 8.453 3.954 4.855<br>H34 6.167 3.451 4.012<br>H35 6.768 2.010 3.199<br>H36 5.619 -0.161 7.404<br>H37 4.828 2.647 8.321<br>H38 6.478 2.041 8.155<br>H39 6.096 0.338 9.893<br>H40 5.750 1.946 10.524<br>H41 3.312 1.580 10.184<br>H42 3.981 0.303 11.199<br>H43 2.472 -0.634 9.426<br>H44 4.124 -1.214 9.227<br>H45 3.165 -0.510 7.047<br>H46 2.847 1.104 7.677<br>H47 3.404 -0.199 4.784<br>H48 6.187 -1.455 4.874<br>H49 4.944 -1.674 6.100<br>H50 4.928 -3.562 4.491<br>H51 3.411 -2.667 4.428<br>H52 5.749 -2.367 2.472<br>H53 4.214 -3.160 2.123<br>H54 3.002 -1.010 2.452<br>H55 4.260 -0.815 1.232<br>H56 4.281 1.098 2.844<br>H57 5.783 0.174 2.921	
**GrI_NC**	**Ind_NC**
Ru1 2.002 1.506 3.469<br>Cl1 2.882 2.333 5.494<br>C1 3.131 2.239 2.235<br>Cl2 0.177 0.793 2.152<br>P2 2.904 -0.547 3.627<br>C2 2.923 3.619 1.841<br>H8 2.509 7.374 0.935<br>C3 3.974 4.330 1.240<br>C4 3.826 5.672 0.920<br>C5 2.625 6.322 1.188<br>C6 1.568 5.627 1.772<br>C7 1.710 4.288 2.093<br>C26 2.878 -1.509 2.035<br>C27 3.404 -0.771 0.801<br>C28 3.100 -1.577 -0.459<br>C29 3.672 -2.988 -0.376<br>C30 3.170 -3.711 0.868<br>C31 3.487 -2.912 2.130<br>C32 4.598 -0.535 4.415<br>C33 5.671 0.059 3.497<br>C34 6.979 0.267 4.255<br>C35 7.465 -1.032 4.883<br>C36 6.394 -1.627 5.790<br>C37 5.085 -1.852 5.035<br>C38 1.895 -1.606 4.787<br>C39 1.740 -0.933 6.156<br>C40 1.051 -1.870 7.143<br>C41 -0.305 -2.320 6.613<br>C42 -0.163 -2.970 5.242<br>C43 0.529 -2.041 4.247<br>H1 4.019 1.752 1.812<br>H2 4.914 3.817 1.039<br>H3 4.649 6.213 0.458<br>H4 0.625 6.133 1.968<br>H5 0.865 3.739 2.514<br>H39 1.790 -1.609 1.879	Ru1 6.509 8.847 10.913<br>Cl1 6.387 6.692 11.875<br>Cl2 7.327 11.062 10.809<br>P1 7.564 8.259 9.022<br>C22 7.467 9.544 7.679<br>C23 8.186 9.186 6.373<br>C24 8.384 10.451 5.541<br>C25 7.041 11.112 5.246<br>C26 6.266 11.402 6.527<br>C27 6.092 10.150 7.384<br>C28 9.399 8.152 9.415<br>C29 10.187 9.445 9.169<br>C30 11.665 9.248 9.505<br>C31 11.861 8.778 10.940<br>C32 11.068 7.504 11.200<br>C33 9.593 7.707 10.874<br>C34 7.009 6.557 8.436<br>C35 8.044 5.441 8.618<br>C36 7.370 4.083 8.430<br>C37 6.686 3.973 7.071<br>C38 5.715 5.127 6.842<br>C39 6.420 6.470 7.025<br>H25 8.033 10.352 8.171<br>H26 7.572 8.484 5.794<br>H27 9.147 8.682 6.558<br>H28 9.028 11.154 6.095<br>H29 8.910 10.212 4.608<br>H30 7.185 12.033 4.667<br>H31 6.445 10.435 4.610<br>H32 5.284 11.834 6.294<br>H33 6.804 12.157 7.123<br>H34 5.582 10.409 8.319<br>H35 5.452 9.425 6.854<br>H36 9.809 7.369 8.753<br>H37 9.767 10.249 9.793<br>H38 10.101 9.774 8.126

H40 4.492 -0.613 0.886<br>H41 2.927 0.213 0.728<br>H42 2.007 -1.628 -0.584<br>H43 3.490 -1.052 -1.340<br>H44 4.773 -2.930 -0.337<br>H45 3.423 -3.559 -1.280<br>H46 3.609 -4.715 0.939<br>H47 2.079 -3.849 0.799<br>H48 4.444 0.189 5.233<br>H49 4.582 -2.828 2.236<br>H50 3.123 -3.449 3.017<br>H51 5.331 1.019 3.091<br>H52 5.848 -0.618 2.643<br>H53 6.811 1.019 5.043<br>H54 7.739 0.683 3.582<br>H55 8.395 -0.868 5.442<br>H56 7.703 -1.754 4.084<br>H57 6.207 -0.941 6.632<br>H58 6.737 -2.574 6.227<br>H59 4.338 -2.265 5.725<br>H60 5.245 -2.606 4.250<br>H61 2.499 -2.520 4.924<br>H62 1.142 -0.015 6.040<br>H63 2.709 -0.604 6.553<br>H64 1.692 -2.753 7.309<br>H65 0.946 -1.373 8.115<br>H66 -0.788 -3.009 7.319<br>H67 -0.966 -1.442 6.529<br>H68 0.426 -3.898 5.340<br>H69 -1.144 -3.265 4.849<br>H70 0.630 -2.552 3.280<br>H71 -0.094 -1.153 4.060	H39 12.095 8.501 8.816<br>H40 12.208 10.185 9.325<br>H41 11.512 9.564 11.629<br>H42 12.927 8.624 11.153<br>H43 11.174 7.181 12.243<br>H44 11.465 6.686 10.575<br>H45 9.014 6.806 11.105<br>H46 9.191 8.497 11.536<br>H47 6.202 6.345 9.155<br>H48 8.852 5.562 7.875<br>H49 8.505 5.482 9.612<br>H50 8.107 3.279 8.555<br>H51 6.626 3.954 9.233<br>H52 7.454 3.993 6.279<br>H53 6.169 3.010 6.977<br>H54 4.878 5.058 7.556<br>H55 5.274 5.068 5.838<br>H56 5.725 7.296 6.819<br>H57 7.230 6.548 6.284<br>C120 4.793 8.963 10.252<br>C122 3.788 9.781 10.962<br>C123 3.921 10.719 11.971<br>H125 4.898 10.946 12.397<br>C124 2.776 11.395 12.409<br>H129 2.858 12.127 13.209<br>C126 1.544 11.158 11.816<br>H133 0.669 11.709 12.152<br>C128 1.412 10.230 10.775<br>H134 0.449 10.089 10.290<br>C130 2.533 9.528 10.366<br>C131 2.739 8.537 9.291<br>C132 4.075 8.260 9.213<br>H135 4.509 7.515 8.557<br>C40 1.705 7.923 8.468<br>C41 0.414 7.691 8.963<br>H136 0.177 7.964 9.988<br>C42 -0.541 7.063 8.175<br>H138 -1.535 6.879 8.578<br>C43 -0.230 6.661 6.881<br>H140 -0.982 6.173 6.265<br>C44 1.049 6.885 6.376<br>H142 1.298 6.576 5.363<br>C45 2.005 7.510 7.161<br>H144 2.998 7.707 6.758
**GrII_NC_nnas**	**GrII_NC_nas**
Ru1 2.278 3.436 5.269<br>Cl1 0.212 2.406 4.719<br>Cl2 4.341 3.544 6.411<br>N1 1.053 6.233 5.154<br>N2 0.977 5.309 7.105<br>C1 1.440 5.109 5.830<br>C2 0.368 7.107 5.993<br>C3 0.320 6.528 7.209<br>C4 1.434 6.602 3.825<br>C5 2.675 7.220 3.649<br>C6 3.059 7.542 2.348<br>C7 2.243 7.256 1.255<br>C8 1.005 6.651 1.480<br>C9 0.575 6.314 2.760<br>C10 3.602 7.441 4.806<br>C11 2.685 7.573 -0.143<br>C12 -0.735 5.626 2.992<br>C13 1.286 4.492 8.249<br>C14 2.441 4.806 8.980	Ru1 13.995 4.332 8.298<br>Cl2 13.721 5.355 6.177<br>Cl3 15.218 3.186 9.969<br>N4 12.301 6.152 10.021<br>C5 13.450 5.845 9.376<br>C6 8.969 5.032 8.912<br>H7 8.258 5.238 8.110<br>H8 10.593 1.440 8.357<br>C9 10.523 6.732 7.886<br>H10 11.326 6.345 7.240<br>H11 10.890 7.678 8.307<br>H12 9.658 6.956 7.253<br>C13 8.664 4.056 9.861<br>C14 15.704 6.852 9.240<br>C15 12.502 3.275 8.282<br>C16 17.464 7.350 7.696<br>H17 17.795 7.772 6.746<br>C18 10.167 5.740 8.951<br>C19 12.503 2.026 7.556

C15 2.746 4.010 10.078<br>C16 1.932 2.946 10.461<br>C17 0.758 2.713 9.750<br>C18 0.400 3.484 8.644<br>C19 3.316 5.963 8.609<br>C20 2.316 2.077 11.620<br>C21 -0.915 3.274 7.959<br>C40 2.858 3.842 3.582<br>C41 3.397 2.810 2.726<br>C42 3.396 1.452 3.097<br>C43 3.942 0.495 2.261<br>C44 4.492 0.871 1.036<br>C45 4.494 2.208 0.649<br>C46 3.951 3.171 1.486<br>H1 -0.005 8.052 5.623<br>H2 -0.107 6.851 8.148<br>H3 4.029 8.012 2.185<br>H4 0.360 6.423 0.630<br>H5 3.095 7.911 5.659<br>H6 4.445 8.076 4.516<br>H7 4.009 6.484 5.166<br>H8 3.637 8.113 -0.154<br>H9 1.943 8.185 -0.669<br>H10 2.814 6.655 -0.732<br>H11 -1.298 5.535 2.059<br>H12 -1.356 6.171 3.714<br>H13 -0.583 4.618 3.404<br>H14 3.648 4.228 10.651<br>H15 0.085 1.918 10.072<br>H16 3.847 5.758 7.671<br>H17 4.067 6.140 9.384<br>H18 2.741 6.888 8.475<br>H19 1.441 1.599 12.075<br>H20 2.836 2.650 12.396<br>H21 2.995 1.278 11.299<br>H22 -0.790 2.938 6.923<br>H23 -1.496 4.204 7.926<br>H24 -1.508 2.525 8.492<br>H58 2.881 4.857 3.174<br>H59 2.947 1.145 4.044<br>H60 3.936 -0.551 2.558<br>H61 4.917 0.115 0.379<br>H62 4.921 2.497 -0.309<br>H63 3.948 4.222 1.197	C20 9.584 3.797 10.878<br>H21 9.358 3.031 11.621<br>C22 16.221 5.906 11.539<br>H23 16.265 6.762 12.228<br>H24 16.896 5.136 11.925<br>H25 15.208 5.492 11.570<br>C26 12.428 7.362 10.844<br>H27 12.504 7.088 11.908<br>H28 11.545 7.999 10.718<br>C29 11.407 -0.080 7.059<br>H30 10.566 -0.757 7.188<br>C31 10.798 4.478 10.957<br>C32 16.111 7.421 8.026<br>C33 18.397 6.764 8.547<br>C34 16.629 6.329 10.159<br>C35 11.823 4.122 11.991<br>H36 11.446 3.346 12.665<br>H37 12.110 4.985 12.605<br>H38 12.744 3.745 11.521<br>C39 13.569 1.649 6.717<br>H40 14.408 2.329 6.561<br>C44 19.839 6.651 8.150<br>H45 20.116 7.410 7.410<br>H46 20.045 5.669 7.705<br>H47 20.502 6.758 9.015<br>C48 12.474 -0.441 6.241<br>H49 12.465 -1.401 5.729<br>N50 14.323 6.860 9.603<br>C51 11.067 5.445 9.983<br>C52 7.390 3.272 9.762<br>H53 6.567 3.887 9.380<br>H54 7.089 2.864 10.732<br>H55 7.504 2.426 9.072<br>C56 15.163 8.174 7.143<br>H57 14.127 7.843 7.263<br>H58 15.422 8.042 6.088<br>H59 15.216 9.249 7.367<br>C60 13.553 0.426 6.069<br>H61 14.381 0.143 5.424<br>C62 11.420 1.145 7.710<br>C63 17.967 6.282 9.782<br>H64 18.694 5.857 10.474<br>C70 13.711 7.984 10.310<br>H71 13.518 8.812 9.608<br>H72 14.377 8.350 11.099<br>H73 11.589 3.499 8.840
**IMes**	**SIMes**
N1 1.036 5.824 5.179<br>N2 0.980 5.014 7.129<br>C1 1.484 4.740 5.885<br>C2 0.296 6.723 5.944<br>C3 0.258 6.203 7.190<br>C4 1.304 6.009 3.789<br>C5 2.564 6.470 3.400<br>C6 2.800 6.658 2.040<br>C7 1.821 6.392 1.083<br>C8 0.580 5.924 1.511<br>C9 0.302 5.720 2.861<br>C10 3.625 6.732 4.424<br>C11 2.109 6.571 -0.378<br>C12 -1.024 5.186 3.310<br>C13 1.182 4.159 8.254<br>C14 2.113 4.525 9.228<br>C15 2.277 3.683 10.327	N1 11.616 8.487 5.656<br>N2 11.614 7.974 3.576<br>C3 11.810 7.453 4.807<br>C4 11.424 9.797 4.997<br>H5 12.344 10.397 5.080<br>H6 10.610 10.365 5.462<br>C7 11.128 9.371 3.569<br>H8 10.052 9.393 3.334<br>H9 11.648 9.974 2.815<br>C10 11.926 8.422 7.042<br>C11 10.893 8.527 7.980<br>C12 11.216 8.480 9.334<br>H13 10.415 8.547 10.072<br>C14 12.532 8.332 9.767<br>C15 13.537 8.220 8.808<br>H16 14.571 8.090 9.132<br>C17 13.259 8.265 7.444

C16 1.550 2.503 10.460	C18 9.467 8.656 7.536
C17 0.638 2.166 9.459	H19 8.779 8.391 8.345
C18 0.440 2.977 8.346	H20 9.261 8.007 6.676
C19 2.924 5.776 9.080	H21 9.223 9.683 7.228
C20 1.760 1.595 11.634	C22 12.863 8.309 11.230
C21 -0.524 2.600 7.264	H23 13.754 7.705 11.430
H1 -0.123 7.630 5.530	H24 12.036 7.902 11.822
H2 -0.204 6.555 8.103	H25 13.065 9.319 11.608
H3 3.777 7.021 1.719	C26 14.351 8.121 6.428
H4 -0.192 5.703 0.773	H27 15.331 8.057 6.910
H5 3.286 7.450 5.182	H28 14.370 8.969 5.730
H6 4.535 7.125 3.959	H29 14.195 7.223 5.818
H7 3.874 5.808 4.961	C30 11.514 7.179 2.401
H8 2.852 7.358 -0.548	C31 10.435 6.296 2.256
H9 1.204 6.831 -0.937	C32 10.344 5.548 1.087
H10 2.509 5.648 -0.819	H33 9.512 4.853 0.967
H11 -1.612 4.828 2.459	C34 11.290 5.661 0.068
H12 -1.620 5.947 3.831	C35 12.356 6.538 0.247
H13 -0.890 4.356 4.015	H36 13.117 6.621 -0.530
H14 3.001 3.955 11.096	C37 12.487 7.304 1.405
H15 0.065 1.242 9.549	C38 9.421 6.145 3.350
H16 3.328 5.862 8.063	H39 8.627 5.451 3.057
H17 3.760 5.786 9.787	H40 8.955 7.105 3.608
H18 2.326 6.679 9.258	H41 9.895 5.776 4.268
H19 0.812 1.180 11.995	C42 11.148 4.862 -1.193
H20 2.240 2.118 12.468	H43 10.800 3.843 -0.985
H21 2.401 0.744 11.369	H44 12.098 4.791 -1.734
H22 0.005 2.458 6.313	H45 10.417 5.317 -1.874
H23 -1.266 3.391 7.092	C46 13.659 8.220 1.590
H24 -1.056 1.676 7.513	H47 14.473 7.956 0.908
	H48 14.036 8.175 2.618
	H49 13.397 9.268 1.390
